# RUNX1-ETO and RUNX1-EVI1 Differentially Reprogram the Chromatin Landscape in t(8;21) and t(3;21) AML

**DOI:** 10.1016/j.celrep.2017.05.005

**Published:** 2017-05-23

**Authors:** Justin Loke, Salam A. Assi, Maria Rosaria Imperato, Anetta Ptasinska, Pierre Cauchy, Yura Grabovska, Natalia Martinez Soria, Manoj Raghavan, H. Ruud Delwel, Peter N. Cockerill, Olaf Heidenreich, Constanze Bonifer

**Affiliations:** 1 Institute for Cancer and Genomic Sciences, College of Medicine and Dentistry, University of Birmingham, B15 2TT Birmingham, UK; 2 Northern Institute for Cancer Research, University of Newcastle, Newcastle upon Tyne NE2 4HH, UK; 3 Department of Hematology, Erasmus University Medical Center, Dr. Molewaterplein 50, 3015 GE Rotterdam, the Netherlands

**Keywords:** RUNX1-EVI1, RUNX1-ETO, epigenetic landscape, chromatin, transcriptional network, acute myeloid leukemia

## Abstract

Acute myeloid leukemia (AML) is a heterogeneous disease caused by mutations in transcriptional regulator genes, but how different mutant regulators shape the chromatin landscape is unclear. Here, we compared the transcriptional networks of two types of AML with chromosomal translocations of the *RUNX1* locus that fuse the RUNX1 DNA-binding domain to different regulators, the t(8;21) expressing RUNX1-ETO and the t(3;21) expressing RUNX1-EVI1. Despite containing the same DNA-binding domain, the two fusion proteins display distinct binding patterns, show differences in gene expression and chromatin landscape, and are dependent on different transcription factors. RUNX1-EVI1 directs a stem cell-like transcriptional network reliant on GATA2, whereas that of RUNX1-ETO-expressing cells is more mature and depends on RUNX1. However, both types of AML are dependent on the continuous expression of the fusion proteins. Our data provide a molecular explanation for the differences in clinical prognosis for these types of AML.

## Highlights

• t(8;21) and t(3;21) AML are regulated by unique gene regulatory networks
• RUNX1-ETO and RUNX1-EVI1 have different binding patterns and binding partners
• Depletion of each fusion protein initiates C/EBPα-dependent myeloid differentiation
• t(8;21) AML survival depends on RUNX1, whereas t(3;21) AML survival requires GATA2

## Graphical Abstract

**Figure:**
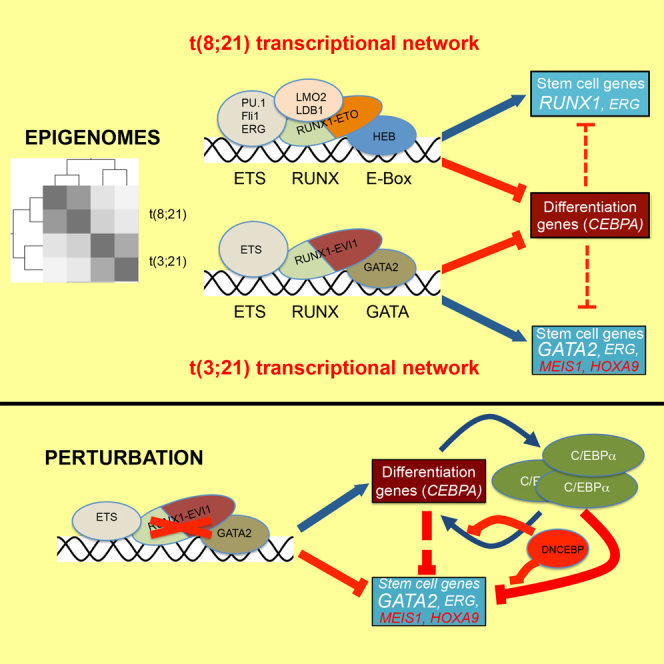

## Teaser

Loke et al. compare the regulatory signature of two related types of acute myeloid leukemia, t(8;21) expressing RUNX1-ETO and t(3;21) expressing RUNX1-EVI1. Each fusion protein displays a distinct binding pattern and cooperates with different transcription factors to impact the epigenome, but both downregulate the myeloid differentiation regulator C/EBPα.

## Introduction

Acute myeloid leukemia (AML) is the most common acute leukemia in adults. Despite improvements in supportive care, outcome typically remains poor for AML patients older than 60 years who are unfit for intensive chemotherapy (Dennis et al., 2015). AML is highly heterogeneous and has been subdivided according to different categories of disease-causing mutations associated with different therapeutic responses. Subclasses are primarily defined by mutations in transcription factors, epigenetic regulators, and signaling molecules that affect cell growth and transcription factor activity (Cancer Genome Atlas Research Network et al., 2013, Papaemmanuil et al., 2016). Consequently, myeloid differentiation is impaired at different developmental stages, and different sets of genes are activated or repressed in distinct subsets of AML. Currently, the molecular details of how specific mutant transcriptional regulator proteins affect different sets of genes, and how such deregulated transcriptional networks impact myeloid differentiation, are unknown.

**Figure 1:**
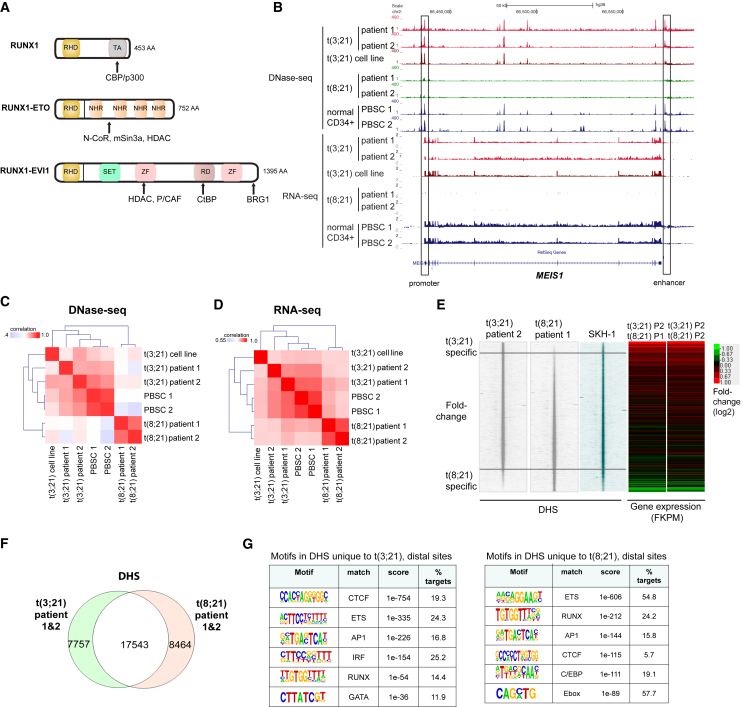
t(3;21) and t(8;21) Are Epigenetically Distinct Types of AML (A) Structure of RUNX1, RUNX1-ETO, and RUNX1-EVI1 with their interacting partners. AA, amino acids; RHD, Runt homology domain; TA, transactivation domain; NHR, nervy homology region; SET, Su(var)3-9 and “Enhancer of zeste”; ZF, zinc finger domain; RD, proline-rich repressive domain; CBP, CREB-binding protein; HDAC, histone deacetylase; CtBP, C-terminal-binding protein; N-CoR, nuclear receptor co-repressor. (B) UCSC genome browser screenshot of DNase-seq and corresponding RNA-seq in two patients with t(3;21) AML, two patients with t(8;21) AML, t(3;21) cell line, and normal CD34^+^ PBSCs at the *MEIS1* locus. An enhancer (Xiang et al., 2014) denoted at +140 kb is accessible in t(3;21) AML and normal CD34^+^ PBSCs, but not in t(8;21) AML. (C) Clustering based on the strength of correlation between samples of DNase-seq data from cells of two patients with t(3;21), two patients with t(8;21), two independent CD34^+^ PBSCs, and the t(3;21) SKH-1 cell line. (D) Correlation clustering of RNA-seq data (as in C) from two t(3;21) patients and the SKH-1 cell line with two t(8;21) patients and two normal CD34^+^ PBSCs. (E) DNase-seq profiles spanning 4-kb windows for t(3;21) patient 2, t(8;21) patient 1, and SKH-1 cells. Peaks are ranked from top to bottom in order of increasing relative DNA sequence tag count for peaks identified in t(8;21) patient 1 relative to t(3;21) patient 2. The heatmaps to the right depict the relative expression of genes nearest to each DHS calculated as the ratio of FPKM values for t(3;21) patient 2 (P2) divided by values for t(8;21) patient 1 (P1) or patient 2 (P2). (F) Venn diagram showing the overlap of DNase-seq peaks between t(3;21) patients (both patients combined) and t(8;21) patients (both patients combined). (G) De novo motif discovery in distal DHSs unique to t(3;21) as compared to t(8;21) patients and distal DHSs unique to t(8;21) compared to t(3;21) patients (as shown in F). See also Figure S1.

Mutations involving the hematopoietic master regulator RUNX1 are among the most commonly found abnormalities in AML. RUNX1 is the DNA-binding component of core binding factor (CBF), binding as a dimer with CBFβ, which is encoded by another recurrently rearranged gene in AML. The most common category of RUNX1 rearrangement is the product of the t(8;21) chromosomal translocation, RUNX1-ETO, which comprises the RUNX1 DNA-binding domain linked to the almost complete ETO protein (also known as RUNX1T1), which functions as a repressor by recruiting histone deacetylases (Bae et al., 1993, Erickson et al., 1992) (Figure 1A). The t(8;21) translocation involves 12% of newly diagnosed younger patients with AML (Grimwade et al., 2010). RUNX1-ETO leads to a block in myeloid differentiation (Cabezas-Wallscheid et al., 2013, Okuda et al., 1998, Regha et al., 2015), and its expression is required for leukemic propagation (Dunne et al., 2006, Heidenreich et al., 2003, Martinez et al., 2004, Ptasinska et al., 2012).

The product of another *RUNX1* translocation, t(3;21)(q26;q22), is RUNX1-EVI1, whereby the RUNT domain is fused to the entire *EVI1* gene (Figure 1A) (Mitani et al., 1994, Nucifora et al., 1994). *EVI1* (also known as *MECOM* or *PRDM3*) encodes a dual domain zinc-finger transcription factor with direct DNA-binding activity together with a histone methyl transferase (SET) domain (Morishita et al., 1995) (Figure 1A) and is an essential regulator of self-renewal in hematopoietic stem cells (Goyama et al., 2008). The t(3;21) translocation is rarely found in patients with de novo AML (Lugthart et al., 2010) and is more commonly found in those with therapy-related myelodysplastic syndrome (MDS)/AML (Rubin et al., 1990) or as a secondary event in the transformation of chronic myelogenous leukemia (CML) from chronic phase to blast crisis (Nukina et al., 2014).

Although RUNX1-ETO and RUNX1-EVI1 carry the same DNA-binding domain and bind to the same motifs in vitro (Meyers et al., 1993, Tanaka et al., 1995), the two classes of AML have distinct clinical characteristics. The t(8;21) translocation generally has a better clinical outcome than the t(3;21) translocation (Byrd et al., 2002, Grimwade et al., 2010, Slovak et al., 2000), and the 5-year event-free survival for t(3;21) patients is only 14% (Lugthart et al., 2010). However, animal models with RUNX1-ETO and RUNX1-EVI1 expression do show similarities. Mice carrying *RUNX1-EVI1* knocked into the *RUNX1* locus display a phenotype similar to the *RUNX1-ETO* knockin (Maki et al., 2005, Okuda et al., 1998, Yergeau et al., 1997), as they die at embryonic day 13.5 (E13.5) with a failure of adult hematopoiesis. RUNX1-ETO and RUNX1-EVI1 also both require additional secondary mutations before they can cause AML in mice (Cuenco et al., 2000, Cuenco and Ren, 2001, Yuan et al., 2001), but RUNX1-EVI1 promotes a more aggressive leukemia with a reduced latency (Cuenco et al., 2000, Maki et al., 2006, Schessl et al., 2005, Schwieger et al., 2002). The molecular mechanisms underlying these similarities and differences in tumor pathology and clinical response are unclear. To address these issues, we compared the gene expression profiles as well as the chromatin landscape and transcription factor occupancy patterns of patients carrying the t(8;21) and t(3;21) translocations using global DNase I hypersensitive site (DHS) mapping, digital DNase I footprinting, and chromatin immunoprecipitation sequencing (ChIP-seq). These studies revealed that RUNX1-ETO and RUNX1-EVI1 associate with distinct subsets of regulatory elements that bind different classes of transcription factors and deregulate different sets of genes. As previously observed for RUNX1-ETO, depletion of RUNX1-EVI1 in t(3;21) cells initiates myeloid differentiation, which is linked to the upregulation of genes known to be vital for myeloid differentiation. Importantly, initiation of differentiation in either type of AML requires the presence of the master regulator of terminal myeloid differentiation, C/EBPα. Hence, despite having the same DNA-binding domain, our data show that the two different RUNX1 fusion proteins maintain the block in differentiation via unique gene regulatory networks.

## Results

### t(3;21) and t(8;21) AML Display Different Epigenetic Landscapes and Gene Expression Profiles

In order to obtain a first indication of the similarities and differences in the cistromes regulating gene expression patterns in t(8;21) and t(3;21) AML we mapped the accessible chromatin landscape by identifying all DHSs in purified CD34^+^ leukemic blast cells of two t(3;21) and two t(8;21) AML patients, two sets of normal CD34^+^ progenitor cells purified as mobilized peripheral blood stem cells (PBSCs) from peripheral blood, and a t(3;21) cell line derived from a CML patient in blast crisis (SKH-1; Mitani et al., 1994)). We performed DNase I sequencing (DNase-seq) to identify all DHSs within chromatin as described previously (Ptasinska et al., 2012), and analyzed gene expression profiles using RNA sequencing (RNA-seq). These comparisons uncovered profound differences in gene expression profiles and DHS patterns between t(8;21) and t(3;21) AML, in particular with HOXA-associated genes such as *HOXA9* and its partner gene, *MEIS1*, which are highly expressed in t(3;21) malignancies, but not in t(8;21) AML (Figures 1B, S1A, and S1B). The SKH-1 cell line proved to be a surprisingly good model of primary t(3;21), as on average 90% of its DHSs overlapped with each of the two primary AMLs (Figures S1B, S1D, and S1E), despite the fact that all three cell types have a very different mutational background (Table S2). Correlation clustering analyses showed that DHS and gene expression profiles of t(3;21) and t(8;21) patients clustered separately as two distinct groups (Figures 1C and 1D) and showed differential gene expression and DHS patterns (Figures 1E, 1F, and S1D–S1F). Interestingly, for both DNase-seq and RNA-seq data, t(3;21) cells clustered closer to normal CD34^+^ cells (PBSCs) than t(8;21) cells (Figures 1 C and 1D), suggesting a status close to early progenitor and stem cells for this type of AML. Furthermore, although RUNX, ETS, AP-1, and CTCF motifs were shared between the DNA motifs present within distal DHSs specific for the two patient classes, t(3;21) patients exhibited a specific enrichment for GATA motifs, whereas DHSs specific for t(8;21) were enriched in motifs for CEBP and E-box-binding factors, as observed previously (Figure 1G) (Ptasinska et al., 2014). In contrast, 90% of DHSs that are common to both t(3;21) and t(8;21) cells were also shared with normal CD34^+^ PBSCs. Consistent with this finding, DHSs common to both types of AML regulate housekeeping functions (Figure S1C, right panel).

### RUNX1-ETO and RUNX1-EVI1 Are Recruited to an Overlapping but Distinct Set of Binding Sites

The differential enrichment for GATA, CEBP, and E-box motifs prompted us to examine whether the binding patterns of RUNX1-ETO, RUNX1-EVI1, and RUNX1 differ between patient groups or whether the shared RHD DNA-binding domain would lead to similar binding pattern. This question is of significant interest, because RUNX1 and RUNX1-ETO have only one DNA-binding domain, whereas RUNX1-EVI1 has two additional potential DNA-binding domains derived from EVI1 (the zinc-finger domains; Figure 1A), which can contribute additional DNA specificity. It has been previously shown by in vitro studies that EVI1 binds to the GATA-like sequence GA(C/T)AAGA(T/C)AAGATAA (Delwel et al., 1993) and TGACAAGATAA (Perkins et al., 1991), which resemble one of the t(3;21)-specific motifs (Figure 1G). To investigate the in vivo specificity of the fusion proteins compared to RUNX1, we first generated RUNX1 and RUNX1-EVI1 ChIP-seq data from SKH-1 t(3;21) cells. We then compared these data with previously published RUNX1-ETO and RUNX1 ChIP data from the t(8;21) Kasumi-1 cell line (Ptasinska et al., 2012), as well as previously published RUNX1-binding data from primary CD34^+^ cells (Cauchy et al., 2015). Both t(8;21) and t(3;21) AML co-express their respective fusion proteins together with wild-type RUNX1, but no expression of either EVI1 (Figure S2A) or ETO was detected, as reported previously (Mitani et al., 1994, Ptasinska et al., 2014). ETO and EVI1 antibodies therefore detected the fusion proteins, whereas the C-terminal RUNX1 antibody detected wild-type RUNX1 (Figure S2A, red).

**Figure 2:**
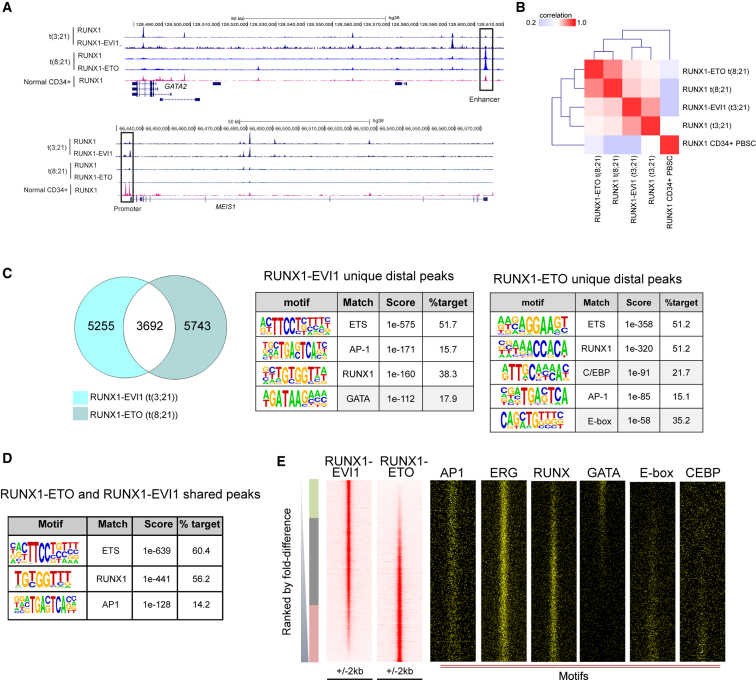
RUNX1-ETO and RUNX1-EVI1 Bind to Different Sites in Each Type of AML (A) UCSC genome browser screen shot of aligned reads at *GATA2* and *MEIS1* from ChIP-seq experiments showing binding of RUNX1 (C-terminal antibody) and RUNX1-EVI1 from t(3;21) SKH-1 cells, RUNX1 and RUNX1-ETO from t(8;21) Kasumi-1 cells, and RUNX1 from normal CD34^+^ PBSCs. The boxed element indicates the *GATA2* enhancer. (B) Matrix-based depiction of the correlation between ChIP-seq experiments followed by hierarchical clustering. (C) Venn diagram of peak overlap between ChIP-seq for RUNX1-EVI1 in t(3;21) SKH-1 versus RUNX1-ETO in t(8;21) Kasumi-1. Tables depict de novo motif analyses of distal sites bound uniquely by each fusion protein. Gray highlights motifs found uniquely in either RUNX1-EVI1- or RUNX1-ETO-bound sites. (D) Motif enrichment analysis in RUNX1-ETO and RUNX1-EVI1 shared peaks. (E) RUNX1-EVI1 ChIP peaks were ranked according to tag count, and RUNX1-ETO peaks were plotted alongside, together with motifs for the indicated transcription factors. See also Figure S2.

**Figure 3:**
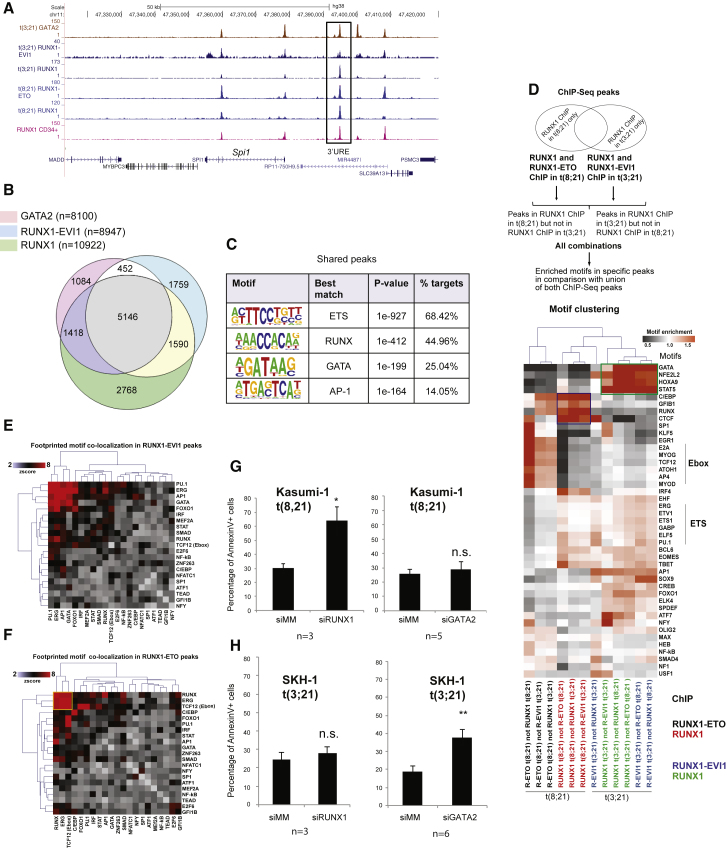
RUNX1 Fusion Proteins Form Part of a Gene Regulatory Network Unique to Each Leukemia (A) UCSC genome browser screen shot of *Spi1* showing ChIP-seq data of RUNX1 and RUNX1-EVI1 from t(3;21) SKH-1 cells, RUNX1 and RUNX1-ETO from t(8;21) Kasumi-1 cells, and RUNX1 from normal CD34^+^ PBSCs. (B) GATA2, RUNX1-EVI1, and RUNX1 ChIP-seq in t(3;21) SKH-1 cells. Venn diagram depicting the overlap between GATA2, RUNX1-EVI1, and RUNX1 peaks and the numbers of peaks in each group. White: overlap of GATA2- and RUNX1-EVI1-bound sites; purple: overlap of GATA2 and RUNX1 bound sites; yellow: overlap of RUNX1- and RUNX1-EVI1-bound sites; gray: number of sites bound by all three transcription factors. (C) Transcription-factor-binding motifs enriched in the shared peaks from (B). (D) Hierarchical clustering of enriched motifs discovered in a pairwise comparison between RUNX1 and RUNX1 fusion ChIP-seq peaks between t(3;21) and t(8;21) cells identifying unique peaks for each type of AML. Enrichment score was calculated by the level of motif enrichment in the unique peaks as compared to union of peaks in the pair of experiments. The heatmap depicts the degree of motif enrichment. Two specific sets of enriched motifs unique to each ChIP seq experiment are highlighted: the blue box highlights specifically enriched motifs in RUNX1-bound sites, the green box highlights enriched motifs specific for t(3;21) but not other peaks. (E and F) Bootstrapping analysis of footprinted motifs at RUNX1-EVI1- or RUNX1-ETO-binding sites in patient cells. RUNX1-EVI1-binding sites from the t(3;21) SKH-1 cell line (E) and RUNX1-ETO-binding sites from the t(8;21) Kasumi-1 cell line (F) mapped onto footprints generated from DNase I data of either t(3;21) patient 2 or t(8;21) patient 1, respectively. The heatmap shows the significance of co-localizing footprinted motifs at RUNX1 fusion protein-binding sites for each AML as compared to sampling by chance alone. (G and H) Percentage of Annexin-V-positive cells after 5 days of treatment with a control siRNA (siMM) or with siRNAs specific for RUNX1 and GATA2, respectively, in Kasumi-1 cells (G) and SKH1 cells (H). Each experiment was done at least in triplicate as indicated, and error bars represent SEM. ^∗^p < 0.05 and ^∗∗^p < 0.01 by unpaired t test. n.s. not significant. See also Figure S3.

A variety of tools were used in combination to analyze the ChIP datasets to demonstrate that despite similar total numbers of binding sites and genomic distribution, the two fusion proteins and RUNX1 each bind to overlapping but largely distinct sets of binding sites (Figures 2A–2D, S2C, S2G, and S3A). In Figures 2E and S2H, we ranked the RUNX1-ETO and the RUNX1-EVI1 or the respective RUNX1 ChIP peaks according to fold difference along the same genomic coordinates (Figures 2E, S2G, and S2H) and then plotted the motifs, again along the same coordinates. These analyses show unequivocally that the two fusion proteins as well as RUNX1 show a distinct binding pattern in each cell type and that GATA motifs partition with RUNX1-EVI1, whereas E-box and C/EBP motifs partition with RUNX1-ETO. The same holds true for RUNX1 binding patterns (Figure S2H). Here, we also plotted our previously reported RUNX1-binding peaks from normal CD34^+^ cells alongside (Ptasinska et al., 2014), supporting the idea that the cistrome of t(3;21) cells is related to that of CD34^+^ cells. At important myeloid regulator genes, such as the CSF-1 receptor gene (*CSF1R)* and the PU.1 gene (*SPI1*), the two fusion proteins target the same regulatory elements (Figures 3A and S3B) (Himes et al., 2001, Li et al., 2001). At many other sites the fusion protein binding sites co-localize specifically with alternate sets of other binding motifs (Figure 2C), such as GATA motifs in t(3;21) and C/EBP and E-Box sites in t(8;21). We did not detect the longer GATA-like motifs in the RUNX1-EVI1 ChIP-seq peaks identified in the in vitro studies (Figure S2E).

ChIP experiments in t(8;21) cells have shown that RUNX1-ETO co-associates with a number of hematopoietic regulators such as the E-box-binding protein HEB; the ETS factors ERG, FLI1, and PU.1; and the LMO2/LDB1 complex (Martens et al., 2012, Ptasinska et al., 2014, Sun et al., 2013). To test whether the GATA motifs in RUNX1-EVI1 peaks were bound by GATA factors, we examined the expression of GATA-family members in t(3;21) and t(8;21) patients and found that *GATA2*, but not *GATA1*, was expressed at a higher level in t(3;21) than in t(8;21) (Figure S3C). Other GATA factors were not expressed at all (Table S1). ChIP experiments demonstrated that RUNX1-EVI1, RUNX1, and GATA2 co-associated within a large population of sequences (Figures 3A, 3B, and S3D), which were characterized by ETS, RUNX, AP-1, and GATA motifs (Figures 3C and S3E). To identify other enriched motifs at the binding sites of RUNX1 and the two fusion proteins, we performed a more refined analysis examining the enrichment of multiple motifs at binding sites specific for each factor (see analysis scheme above Figure 3D) and cell type and then clustered the enrichment p-values (Figure 3D). Such an analysis highlights whether a set of motifs shows a higher enrichment in one cell type as compared to another, indicating the binding of different transcription factors around specific binding sites for each fusion protein and highlighting the relative importance of a transcription factor family in each cell type. For t(8;21) cells, this analysis showed that RUNX1 and RUNX1-ETO sites cluster separately with a strong enrichment of RUNX, C/EBP, and GFI1B motifs for both RUNX1-ETO and RUNX1 peaks (boxed in blue), a selective enrichment of E-box motifs for RUNX1-ETO peaks, and a specific enrichment for ETS family motifs for RUNX1 peaks. In contrast, RUNX1-EVI1 and RUNX1 peaks clustered together in t(3;21), with a strongly enriched motif signature for GATA, STAT, HOXA9, and ETS motifs (boxed in green).

To validate our ChIP data in primary cells, we performed digital DNase I footprinting and identified regions protected from nuclease digestion indicative of transcription factor binding using the Wellington algorithm (Piper et al., 2013). We then filtered footprints against our cell line ChIP data for RUNX1 (in t(8;21) and t(3;21)), RUNX1-EVI1, and RUNX1-ETO. Finally, we performed a bootstrapping analysis, which highlights the significance of occupied motif co-clustering within windows of 50 bp, and plotted enriched motifs in a co-clustering matrix (Figures 3E, 3F, S3F, and S3G). These analyses showed that RUNX1-EVI1-binding sites clustered with occupied PU.1/ERG (ETS), AP-1, and GATA motifs, suggesting that they may exist as a complex. This is in line with the fact that EVI1 has been shown to directly interact with the AP-1 family member FOS in several cell lines (Bard-Chapeau et al., 2012) and to co-localize with AP-1 motifs (Glass et al., 2013). In contrast, RUNX1-bound sites in t(3;21) only co-localized with occupied ERG (ETS) motifs in each cell type. However, the picture for RUNX1-ETO binding was different. Occupied RUNX1-ETO-bound sites clustered together with RUNX, ERG, and E-box motifs (Figure 3D), highlighting the nature of the RUNX1-ETO complex.

### Survival of t(3;21) Cells Depends on the Continuous Expression of GATA2, but Not RUNX1

Our binding data suggested that RUNX1-EVI1 and RUNX1-ETO associate with different transcription factor complexes. Furthermore, such differential binding is also found with the wild-type RUNX1 protein expressed from the non-translocated allele in each AML type, indicating that RUNX1 fulfills different roles in programming the chromatin landscape in each cellular context. It was previously shown that the survival of t(8;21) cells is dependent on the expression of wild-type RUNX1, whereby RUNX1 regulated a complementary set of genes balancing the effects of RUNX1-ETO (Ben-Ami et al., 2013). We therefore tested whether this was also true for t(3;21) cells. To this end, we treated Kasumi-1 and SKH-1 cells with small interfering RNAs (siRNAs) specific for RUNX1 as well as control siRNAs and measured their survival using staining for the apoptosis marker Annexin V and propidium iodide (PI), which indicates dead cells. Figures 3G, 3H (left), and S3H–S3J demonstrate that after 5 days of knockdown, t(8;21) cells showed in increased cell death as compared to control cells, while SKH-1 cells showed no difference and thus do not require wild-type RUNX1.

Previous studies have shown that the members of the RUNX1-ETO complex (namely LMO2 and ERG) are required for the leukemogenicity of RUNX1-ETO and their survival (Sun et al., 2013). To gain first insights into whether GATA2 was preferentially required for the survival of t(3;21) cells, we depleted GATA2 in both t(3;21) and t(8;21) cells by siRNA treatment (Figures 3G and 3H, right, and Figures S3H–S3J) and measured their survival using staining for Annexin V and PI. These experiments show a strong increase in the number of apoptotic and dead cells in t(3;21) cells, but not in t(8;21) cells, indicating that GATA2 plays a more important role in the survival of t(3;21) cells than in t(8;21) cells.

Taken together, our data demonstrate that despite sharing the same DNA-binding domain, the two fusion proteins predominantly bind to different genomic sites, co-bind with different partners, and operate within a different chromatin landscape. Moreover, the transcriptional networks regulating the survival of both types of AML depend on different non-mutated transcription factors, with t(8;21) cell depending on RUNX1 and t(3;21) cell being dependent on GATA2.

### RUNX1-EVI1 Is Required to Maintain the Undifferentiated Phenotype and Survival of t(3;21) AML

**Figure 4:**
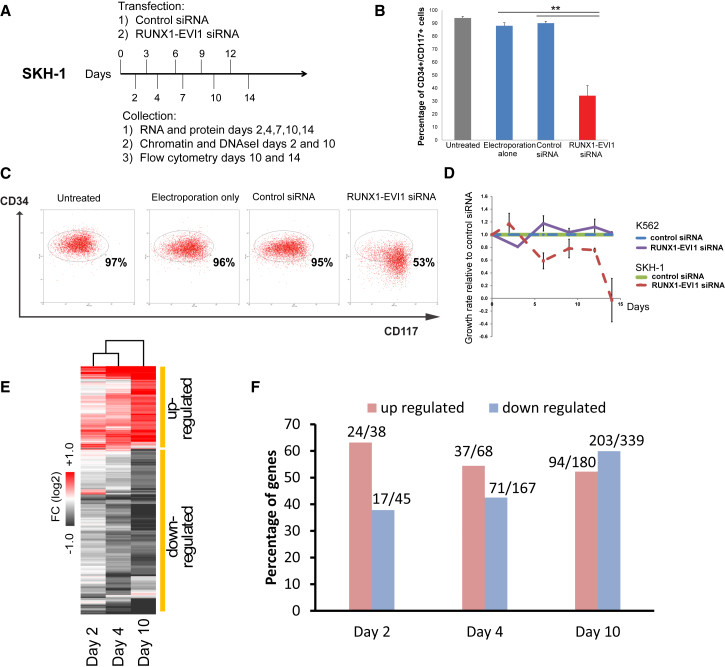
Knockdown of RUNX1-EVI1 Results in Loss of the Stem Cell Gene Program (A) Experimental scheme for the siRNA transfection. (B and C) RUNX1-EVI1 siRNA treatment in SKH-1 cells results in reduction in CD34 surface expression. SKH-1 cells after 14 days of either RUNX1-EVI1 or control siRNA transfection were stained with CD34-PE and CD117-APC. (B) Percentage of CD34^+^CD117^+^ cells. (C) Representative flow cytometry plot. Mean of six independent experiments is shown. Error bars represent SEM. ^∗^p < 0.05 by paired t test. (D) Growth rates of SKH-1 (dashed lines) and K562 cells (solid lines) treated with RUNX1-EVI1 siRNA relative to treatment with control siRNA. The graph shows mean and SEM values from at least three independent experiments. (E) Hierarchical clustering of gene expression changes as determined by RNA-seq at different time points of treatment. Unsupervised clustering of expression values of genes changing expression 1.5-fold after RUNX1-EVI1 siRNA transfection as compared to control siRNA. Average of two independent replicates. The heatmap color is related to the degree of differential expression (fold change [FC]) between RUNX1-EVI1 siRNA and control siRNA treatment. (F) Percentage and number, respectively (on top of bars), of differentially expressed genes as measured by RNA-seq that are RUNX1-EVI1 ChIP-seq targets. Differentially expressed genes are those with an at least 1.5-fold change in gene expression between RUNX1-EVI1 siRNA as compared to control siRNA treatment. See also Figure S4.

**Figure 5:**
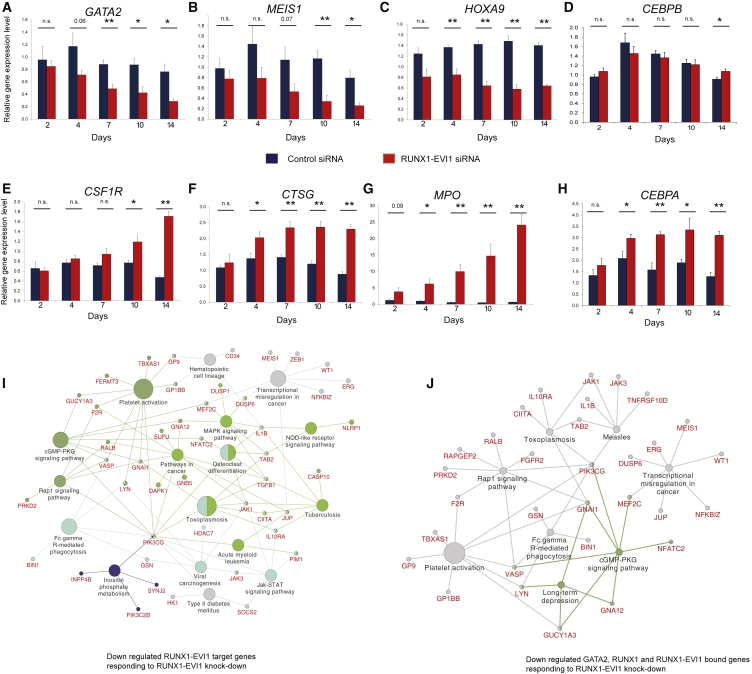
Knockdown of RUNX1-EVI1 Results in Loss of the Expression of Stem Cell Genes and the Upregulation of Myeloid Genes (A–H) RT-PCR analysis of mRNA levels of the indicated genes relative to GAPDH and normalized to untreated cells in SKH-1 cells after RUNX1-EVI1 siRNA as compared to control siRNA transfection. *GATA2* (A), *MEIS1* (B), *HOXA9* (C), *CEBPB* (D), *CSF1R* (E), *CTSG* (F), MPO (G)*,* and *CEBPA* (H). The graph shows mean and SEM of four independent experiments. n.s., not significant; ^∗^p < 0.05; ^∗∗^p < 0.01 (unpaired t test). See also Table S1. (I) KEGG pathways highlighting genes and pathways that are upregulated after RUNX1-EVI1 knockdown. (J) KEGG pathways highlighting pathways associated with genes with shared binding of GATA2, RUNX1, and RUNX1-EVI1 that are upregulated after RUNX1-EVI1 knockdown. See also Figure S5.

The t(8;21) translocation is a driver mutation (Wiemels et al., 2002), and RUNX1-ETO expression is required to maintain the leukemic phenotype in t(8;21) AML (Dunne et al., 2006). In contrast, RUNX1-EVI1 is a secondary mutation found in secondary AML and in CML in blast crisis (Nukina et al., 2014, Paquette et al., 2011, Rubin et al., 1987, Rubin et al., 1990). We therefore used an siRNA knockdown approach to investigate whether RUNX1- was also required to maintain the full leukemic potential of t(3;21) cells. We targeted the siRNA to the junction between RUNX1 and EVI1 to specifically deplete RUNX1-EVI1, but not RUNX1 (Figures S2A and S4A). We transfected SKH-1 with this siRNA and control siRNA, in parallel with control K562 cells, over a period of 2–14 days (Figure 4A). Flow cytometry revealed that SKH-1 cells transfected with RUNX1-EVI1 siRNA, but not control RNA, decreased the expression of the progenitor cell marker CD34 (Figures 4B and 4C). SKH-1 cells treated with siRNA, but not K562 cells, showed a diminished growth rate (Figure 4D) and started to undergo apoptosis (Figures S4B and S4C), indicating that the fusion protein is required for their survival. The analysis of RNA-seq data revealed significant changes in gene expression after knockdown (Figures 4E, 4F, and S4E; Table S1) with genes being progressively up- and downregulated. qPCR analyses confirmed that genes downregulated by siRNA included stem cell genes such as *GATA2* (Figures 5A–5C), whereas upregulated genes included myeloid differentiation markers such as *MPO*, *CSF1R*, *CTSG*, and *CEBPA* (Figures 5E–5H and S4F). The expression of *CEBPB* was unaffected (Figure 5D). Gene set enrichment analysis (GSEA) showed that the cells downregulated a stem cell program after knockdown of RUNX1-EVI1 (Figures S5A and S5B). Kyoto Encyclopedia of Genes and Genomes (KEGG) pathway analysis for RUNX1-EVI1 target genes downregulated after RUNX1-EVI1 knockdown highlighted multiple signaling genes, such as *PIM1*, *DUSP1*, *DUSP6*, *JAK1*, and *JAK3* (Figure 5I). A parallel analysis of upregulated genes identified *CEBPA*, *KIT*, and *MPO* (Figure S5F). A more refined picture was also seen when we analyzed downregulated core genes bound by RUNX1-EVI1, RUNX1, and GATA2 (Figure 5J). This analysis again identified genes encoding for factors important for stem cell function such as ERG, WT1, and MEIS1.

### C/EBPα Is Required for the Response of t(3;21) Cells to RUNX1-EVI1 Knockdown

**Figure 6:**
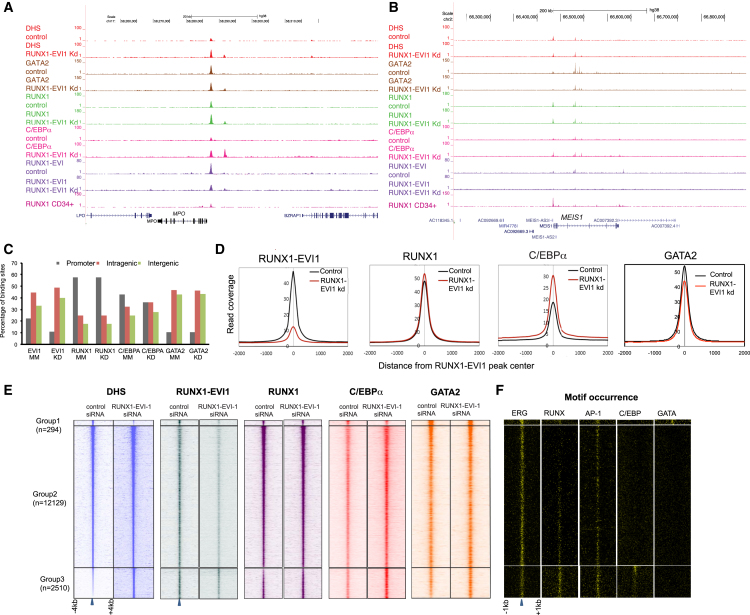
RUNX1-EVI1 Knockdown Results in Genome-wide Reprogramming of the Epigenome (A and B) UCSC genome browser screen shots showing aligned reads at *MPO* (A) and *MEIS1* (B) depicting DNase-seq and RUNX1, RUNX1-EVI1, GATA2, and C/EBPα ChIP-seq data from SKH-1 after either control siRNA or RUNX1-EVI1 siRNA treatment. (C) Genomic distribution of the indicated factors after transfection with either control or RUNX1-EVI1 siRNA. (D) Average profiles of RUNX1-EVI1, RUNX1, C/EBPα, and GATA2 ChIP-seq reads centered on RUNX1-EVI1 peaks within a 4-kb window after transfection with either control or RUNX1-EVI1 siRNA. (E) Profiles of the DNase-seq and ChIP-seq tag density for the indicated factors in ±4-kb windows centered on DHS for SKH-1 treated with either control or RUNX1-EVI1 siRNA. All peaks were ranked according to the fold change in DNase-seq tag counts between control siRNA and RUNX1-EVI1 siRNA-treated SKH-1 cells. (F) Densities of the indicated motifs underlying the same coordinates plotted within ±1-kb windows around the DHS marked with a blue arrow. See also Figure S6.

To identify factors that are involved in driving the differentiation of t(3;21) cells after RUNX1-EVI1 knockdown, we examined the changes in the epigenetic landscape of t(3;21) SKH-1 cells by mapping DHSs in cells treated with a control siRNA or after 10 days of knockdown with a RUNX1-EVI1-specific siRNA (Figure S5C). Examples of these data are depicted in the genome browser screenshots shown in Figures 6A, 6B, and S6A. We then ranked our DHS data according fold difference in sequence tag count (Figure S5D). This analysis revealed three groups of elements: a small group of peaks (group 1) unique for control cells, a large number of shared peaks (group 2), and 2,510 peaks that only appeared after knockdown (group 3). A de novo analysis of DNA motifs in these groups revealed that C/EBP motifs were specifically enriched in the DHSs gained after knockdown (Figure S5E). These results were concordant with the downregulation of *GATA2* and the upregulation of *CEBPA* expression after RUNX1-EVI1 knockdown (Figures 5A and 5H). To examine whether these changes in gene expression and motif composition were reflected in changes of binding of the respective factors, we measured the binding of RUNX1, GATA2, and C/EBPα before and after 10 days of RUNX1-EVI1 knockdown (Figures 6A, 6B, and S6A show screenshots). These experiments show that RUNX1-EVI1 knockdown did not influence the overall global genomic distribution of binding sites for these factors (Figure 6C) and did not influence the binding levels of RUNX1, although there was both a decrease in binding at some sites and increases in binding at others (Figures 6E and S6B). GATA2 binding decreased slightly overall and some binding sites were lost (Figures 6D and S6C), which can be explained by the lower expression of the *GATA2* gene (Figure 5A). These findings also demonstrated that GATA2 binding was not categorically dependent on the presence of RUNX1-EVI1. However, C/EBPα binding levels were increased (Figure 6D and 6E) with a number of new binding sites (Figure S6D). An alignment of DNA motifs and the DHS peaks confirmed that following RUNX1-EVI1 knockdown, C/EBP motif containing DHSs increased (group 3) in parallel with a depletion of GATA motif containing DHSs (group 1)(Figure 6F). In contrast, ERG, RUNX, and AP-1 motifs were relatively evenly distributed (Figure 6F). *CEBPA* upregulation is likely to be caused by the reduction of binding of RUNX1-EVI1 after knockdown with a concomitant increase of the binding of RUNX1 and C/EBPα itself to the *CEBPA* locus (Figure S6E).

**Figure 7:**
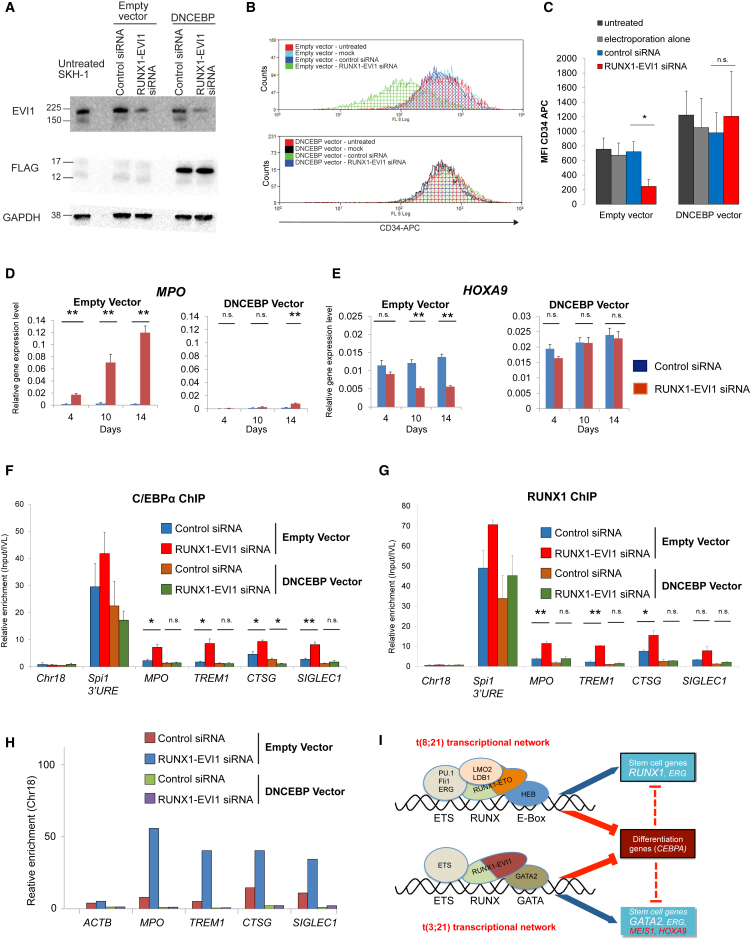
C/EBPα DNA Binding Ability Is Critical for the Effects of RUNX1-EVI1 Knockdown (A) Western blot analysis of whole-cell lysates of untreated t(3;21) SKH-1 cells and empty or DNCEBP vector transduced SKH-1 cells, transfected with either control or RUNX1-EVI1 siRNA after 14 days. Sizes (in kDa) are indicated. The blot was probed with EVI1, FLAG, or GAPDH antibodies (loading control) as indicated. (B and C) Flow cytometry of empty and DNCEBP vector transduced SKH-1 cells, untreated or after 14 days of mock, control, or RUNX1-EVI1 siRNA treatment, stained with CD34-APC. (B) Representative histogram with overlay of different treatment conditions. (C) Graph of median fluorescence intensity (MFI) (median) of CD34-APC staining. Bars represent different treatment conditions. Mean of three independent experiments is shown, and error bars represent SEM. n.s., not significant; ^∗^p < 0.05 (paired t test). (D and E) RT-PCR analysis of mRNA levels of the indicated genes with and without RUNX1-EVI1 knockdown in the presence and absence of DNCEBP. (D) *MPO*. (E) *HOXA9*. mRNA levels relative to GAPDH in either empty vector or DNCEBP vector transduced SKH-1 after either control or RUNX1-EVI1 siRNA transfection (4, 10, or 14 days of treatment). The graph shows mean and SEM of three independent experiments. n.s., not significant; ^∗^p < 0.05; ^∗∗^p < 0.01 (unpaired t test). (F and G) ChIP-qPCR with chromatin from empty or DNCEBP vector transduced SKH-1 10 days after either control or RUNX1-EVI1 siRNA transfection, using amplicons corresponding to the *MPO* and *SIGLEC1* enhancers, *TREM1* and *CTSG* promoters, the *SPI1* 3′ upstream regulatory element (URE) enhancer as a positive control, and chromosome 18 as a negative control. (F) C/EBPα ChIP. (G) RUNX1 ChIP. Enrichment was calculated relative to input and IVL. Mean of three independent experiments is shown, and error bars represent SEM. n.s., not significant; ^∗^p < 0.05; ^∗∗^p < 0.01 (unpaired t test). (H) DNase I accessibility measurement using qPCR validation at *MPO* and *SIGLEC1* enhancer and *TREM1* and *CTSG* promoter. DNase I digestion was performed on empty vector and DNCEBP vector transduced SKH-1 following either control or RUNX1-EVI1 siRNA transfection. The *ACTB* amplicon was used as negative control. Enrichment was calculated relative to chromosome 18, which is a gene-free region that is DNase I inaccessible and used for normalization. A second independent experiment is shown in Figure S7D. (I) Model depicting the binding sites and transcription factors interacting with RUNX1-ETO and RUNX1-EVI1, respectively, and their independent transcriptional networks that maintain the expression of stem cell/precursor genes but also block the expression of *CEBPA.* See also Figure S7.

We next examined whether upregulation of C/EBPα was required for the response to oncogene depletion. To this end, we transduced SKH-1 cells with a lentiviral vector expressing a dominant-negative CEBP peptide (DNCEBP) to block C/EBPα binding (and that of all other C/EBP factors) during knockdown RUNX1-EVI1 by siRNA. The DNCEBP peptide dimerizes with CEBP transcription factors and prevents binding to DNA (Krylov et al., 1995). Expression of the FLAG-epitope-tagged DNCEBP peptide was confirmed by western blotting (Figure 7A). We then treated control and DNCEBP SKH-1 cell lines with RUNX1-EVI1 siRNA (Figures S7A and S7B). While RUNX1-EVI1 knockdown decreased CD34 expression and proliferation, co-expression of the DNCEBP peptide rescued the leukemic phenotype (Figures 7B, 7C, and S7C). Similarly, DNCEBP-expressing cells maintained high expression of *HOXA9* after RUNX1-EVI1 knockdown, slowed the decrease of the key stem cell renewal genes *GATA2* and *MEIS1*, and blocked increased expression of the markers of myeloid differentiation *CTSG*, *MPO*, and *CSF1R* (Figures 7D, 7E, and S7B). ChIP experiments confirmed reduced C/EBPα binding at the corresponding loci after DNCEBP expression (Figure 7F). Another interesting finding was that the expression of DNCEBP also reduced the increase in RUNX1 binding at a set of known C/EBP and RUNX1 target genes (Figures 7F and 7G) with a concomitant reduction in DNase I accessibility at genes strongly activated by RUNX1-EVI1 knockdown, such as *MPO* or *CTSG*, indicating that here, the cooperation of C/EBPα and RUNX1 is required for activation (Figures 7H and S7D).

Taken together, as summarized in Figure 7I, our study highlights how specific oncogenic transcription factors differentially program the epigenetic landscape in two types of AML with RUNX1 translocations but share the feature that they are dependent on the expression of the fusion protein and the suppression of C/EBPα to inhibit differentiation.

## Discussion

The study presented here used global analyses to investigate differences and similarities between two types of CBF AML: the t(8;21) expressing RUNX1-ETO and the t(3;21) expressing RUNX1-EVI1, which both carry the same RUNX1 DNA-binding domain. Our DHS mapping, digital footprinting experiments, and ChIP assays of patient cells and appropriate patient-derived model cell lines unequivocally determined how (1) the epigenetic and transcriptional profiles of the two types of AML differ and (2) show that the RUNT DNA-binding domain of each fusion protein is not the sole determining factor for the selection of fusion protein-binding sites in the genome. Moreover, each type of AML displays a unique, stable transcriptional network that is dependent on the presence of each fusion protein but requires a different set of associated transcription factors.

### t(3;21) and t(8;21) AML Display Alternate Transcriptional Networks

The RUNX1-ETO and RUNX1-EVI1 fusion proteins are both unable to cause leukemia in mice on their own (Cuenco et al., 2000, Okuda et al., 1998), and they show a different history of tumor development in humans. The t(8;21) translocation is a primary mutation that hits an early stem cell (Miyamoto et al., 2000), whereas t(3;21) is often found in CML patients after blast crisis, indicating that during tumor initiation, the two fusion proteins encounter a dramatically different chromatin landscape that dictates where they can bind. Previous studies from our laboratory that used an inducible version of RUNX1-ETO expressed in murine myeloid precursor cells demonstrated that the induction of the fusion protein leads to a rapid downregulation of myeloid genes such as *Spi1*(PU.1) and *Cebpa* and a concomitant increase in the expression of stem cell genes such as *Gata2* and *Erg*, indicating extensive feed-forward loops driving myelopoiesis (Regha et al., 2015). It is likely that the same holds true for RUNX1-EVI1, but a different differentiation stage or previous transformation event may be required for the establishment of a stable transformed transcriptional network incorporating the expression of this powerful oncoprotein.

The unique transcriptional network maintained by RUNX1-EVI1 explains the difference in clinical outcomes of t(3;21) as compared to t(8;21) AML. RUNX1-EVI1 appears to directly regulate a stem cell program establishing an immature phenotype associated with treatment resistance, (Eppert et al., 2011), expressing genes (*MSI2* and *ZEB1*) regulating leukemia aggressiveness (Ito et al., 2010, Stavropoulou et al., 2016). Furthermore, *HOXA9* and *MEIS1* are both expressed in t(3;21), but not in t(8;21), AML. *HOXA9* expression is associated with poor prognosis (Andreeff et al., 2008, Golub et al., 1999), and is linked to a number of mutational subtypes, including mixed lineage leukemia (MLL) and NUP98 translocations (Collins and Hess, 2016). *MEIS1* expression is also associated with poor prognosis as part of a gene expression pattern seen in HSCs and LSCs (Eppert et al., 2011). *HOXA9* and *MEIS1* are often co-expressed in AML (Lawrence et al., 1999) and *Hoxa9* requires the co-expression of *Meis1* to transform murine bone marrow progenitor cells (Kroon et al., 1998). This cooperativity can be explained by the identification of a large number of cis-regulatory elements that are co-bound by both Hoxa9 and Meis1 (Huang et al., 2012).

The different gene regulatory networks maintaining the two types of AML involve alternate sets of transcription factors, and they differentially program the chromatin landscape, thus impacting where the fusion proteins bind. Our data show that RUNX1-ETO-binding sites are enriched for occupied ETS/RUNX/E-box motifs, reflecting the structure of the RUNX1-ETO complex, with the ETS factors ERG and FLI1 in the complex being required for leukemia maintenance and leukemogenesis (Martens et al., 2012, Sun et al., 2013). The expression of *ERG* in AML is generally associated with poor prognosis (Diffner et al., 2013). In contrast, RUNX1-EVI1 co-localizes with bound GATA2 and occupied AP-1 motifs, suggesting association with a different complex. Our data indeed show that high-level GATA2 expression is required for the survival of SKH-1 cells, but not t(8;21) cells, whereas RUNX1 regulates a complementary set of genes and is required for the survival of t(8;21) cells (Ben-Ami et al., 2013), but not SKH-1 cells (this study). High expression of GATA2 is indeed associated with poor prognosis in pediatric AML (Luesink et al., 2012), which may contribute to the fact that the t(3;21) is more aggressive than t(8;21). How each CBF fusion protein complex programs the DHS landscape, causing differential expression of members of the each complex, is exemplified by the regulation of *GATA2*. *GATA2* expression is higher in t(3;21) cells than in t(8;21) cells, which can be explained by a differential activity of its *cis*-regulatory elements. Our data show that a distal *GATA2* enhancer, known to upregulate *GATA2* expression (Gröschel et al., 2014), is accessible and bound by RUNX1 in normal CD34^+^ cells, and by RUNX1 and RUNX1-ETO in t(8;21) patient cells, but neither RUNX1 nor RUNX1-EVI1 binds to this element in t(3;21) cells.

In summary, RUNX1 and both fusion protein complexes bind to AML-type specific *cis*-regulatory modules, which through auto-regulation of genes encoding complex members initiate the formation of stable gene regulatory networks that ultimately define the behavior of each type of AML (Pimanda and Göttgens, 2010).

### C/EBPα Is Required for the Differentiation of t(8;21) and t(3;21) AML Cells after Oncoprotein Knockdown

Despite the differences between t(3;21) and t(8;21) transcriptional networks, C/EBPα is downregulated in both types of AML, suggesting that it is a critical node by which leukemia is maintained. We show that C/EBPα is directly repressed by both RUNX1-EVI1 and RUNX1-ETO through binding to a recently characterized upstream enhancer (Avellino et al., 2016). In both t(8;21) and t(3;21) cells, knockdown of the CBF fusion protein leads to upregulation of *CEBPA*, and our ChIP-seq data directly show that the binding of C/EBPα is affected by the knockdown of both fusion proteins. Conversely, in both AML types, the reduction of C/EBPα-binding activity by either knockdown (Ptasinska et al., 2014) or expression of a dominant-negative version of C/EBP (DNCEBP) blocks myelopoiesis and abolishes the upregulation of genes required for terminal myeloid function (*MPO*, *CSF1R*, and *CTSG*). This result complements previous data showing that overexpression of *CEBPA* can overcome the RUNX1-EVI1-mediated differentiation block (Tokita et al., 2007). An interesting finding from our study is that that the block of C/EBPα binding also abolishes the establishment of specific DHSs at certain genes and the binding of other transcription factors, including RUNX1. C/EBPα interacts with SWI/SNF nucleosome remodeling complexes, and this interaction is important for the development of adipocytes (Pedersen et al., 2001). This ability to initiate a global reprogramming of chromatin structures may be the main driver of C/EBPα-mediated myeloid differentiation, and current experiments focus on the mechanistic details of how this occurs.

Taken together, our study provides an important paradigm for studies aimed at understanding how different leukemic fusion proteins program and interact with the epigenetic landscape in two related but different types of AML. Our data represent a resource that will facilitate global mechanistic studies of the genes, transcription factors, and pathways involved in blocking myeloid differentiation and emphasize that different types of AML, despite being a disease of one specific differentiation pathway, are maintained by highly diverse transcriptional networks. Our study therefore highlights the complexities we have to face in our understanding of AML heterogeneity if we want to use this knowledge to devise AML-specific therapies.

## Experimental Procedures

### Purification of Leukemic Cells and Mobilized Peripheral Stem Cells

Cells were purified as described previously (Cauchy et al., 2015), with minor modifications as outlined in in Supplemental Experimental Procedures.

### siRNA-Mediated Depletion

1 × 10^7^ cells were electroporated using an EPI 3500 (Fischer) at 350 V, 10 ms. siRNA sequences are listed in the Supplemental Experimental Procedures. siRNA was used at 200 nM. After electroporation, the cells remained in their cuvettes for 5 min before being directly added to RPMI-1640 with 10% fetal calf serum (FCS), supplemented with penicillin/streptomycin and glutamine at a concentration of 0.5 × 10^6^ cells/mL, returned to an incubator, and kept at 37°C and 5% CO_2_.

### DHS Mapping, ChIP-Seq, and Digital Footprinting

DHS mapping, ChIP-seq, and digital footprinting using the Wellington algorithm (Piper et al., 2013) was performed as described previously (Cauchy et al., 2015, Ptasinska et al., 2014), with minor modifications as outlined in in Supplemental Experimental Procedures. Details of antibodies and primers for qPCR are listed in Tables S3 and S4. Sequencing read data and list of peak numbers can be found in Table S3.

### Data Analysis

Details of data analyses can be found in Supplemental Experimental Procedures.

### Patient Samples

All human tissue was obtained with the required ethical approval from the National Health Service (NHS) National Research Ethics Committee. Detailed information about patient samples is listed in Table S2.

## Author Contributions

J.L. performed experiments and wrote the paper. M.R.I., A.P., Y.G., and N.M.S. performed experiments. S.A.A. and P.C. analyzed the data. M.R. and P.N.C. helped supervising the study and writing the paper. H.R.D. provided essential research material. O.H. designed and supervised experiments and helped writing the paper. C.B. designed the study, supervised experiments, and wrote the paper.

## Accession Numbers

The accession number for all t(3;21) next generation sequencing data reported in this paper is GEO: GSE87286.

## Supplemental Information

Document S1. Supplemental Experimental Procedures, Figures S1–S6, and Tables S2–S4

Table S1. Gene Expression Levels in Patient Cells and Cell Lines, Related to Figures 1 and 4 (Sheet 1) RNA-Seq data from CD34+ cells purified from patients with either t(3;21) or t(8;21) AML, and from normal PBSCs. Related to Figure 1. RNA-Seq values are expressed as FPKM. (Sheet 2) RNA-Seq data from SKH-1 cells following RUNX1-EVI-1 siRNA transfection as compared to control siRNA transfection. Related to Figure 4. RNA was extracted after 2, 4 and 10 days of treatment. Values are expressed as FPKM values from RNA-Seq in control siRNA (MM) and RUNX1-EVI-1 siRNA (KD) transfected cells. Gene expression fold change (FC) is given following either 2, 4 or 10 days of siRNA transfection.

Document S2. Article plus Supplemental Information
