# Supplementary material for: RUNX1-ETO and RUNX1-EVI1 Differentially Reprogram the Chromatin Landscape in t(8;21) and t(3;21) AML
Source: Cell Rep. 2017 May 23;19(8):1654–68. doi: 10.1016/j.celrep.2017.05.005 (PMC5457485; doi:10.1016/j.celrep.2017.05.005)
Supplement: Document S1. Supplemental Experimental Procedures, Figures S1–S6, and Tables S2–S4 [file mmc1.pdf]

Cell Reports, Volume *19*

Supplemental Information

RUNX1-ETO and RUNX1-EVI1 Differentially Reprogram the Chromatin Landscape in t(8;21) and t(3;21) AML

Justin Loke, Salam A. Assi, Maria Rosaria Imperato, Anetta Ptasinska, Pierre Cauchy, Yura Grabovska, Natalia Martinez Soria, Manoj Raghavan, H. Ruud Delwel, Peter N. Cockerill, Olaf Heidenreich, and Constanze Bonifer

Supplemental information

Contents

Supplemental figures and legends 3
Supplemental Figure 1 (related to Figure 1) 3
Supplemental Figure 2 (related to Figure 2) 5
Supplemental Figure 3 (related to Figure 3) 7
Supplemental Figure 4 (related to Figure 4) 9
Supplemental Figure 5 (related to Figure 5) 11
Supplemental Figure 6 (related to Figure 6) 13
Supplemental Figure 7 (related to Figure 7) 15
Supplemental Experimental Procedures 17
Purification of blood samples from patients with AML 17
Purification of CD34+ mobilized peripheral blood stem cells 17
siRNA mediated depletion of RUNX1-EVI1, RUNX1 and GATA2 17
RNA extraction 18
RNA Seq libraries 18
cDNA synthesis 19
Real-time polymerase chain reaction 19
Dead cell removal and Annexin V/PI staining for flow cytometry 19
DNaseI hypersensitivity site mapping 19
Library production of DNase I material for high throughput sequencing 20
ChIP-qPCR and ChIP-Seq library preparation 20
Double cross-linking 20
Chromatin immunoprecipitation (ChIP) 20
Library production of ChIP material for high throughput sequencing 21
Retroviral production 21
Transfection of HEK293T cells for lentiviral production 21
Virus concentration 22
Lentiviral transduction of SKH-1 22
Antibody staining for flow cytometry 22
Whole cell lysate preparation by RIPA buffer lysis 22
Nuclear extract 22
Western blotting 23
Oligonucleotide sequences and antibodies 23
ChIP and DNase I sequencing data Analysis 25
Alignment 25

Peak calling 25
Clustering of ChIP and DNAseI sequencing data 25
Average tag density profile and heatmap 26
Motif identification and clustering 26
Motif clustering 26
Motif enrichment 26
RNA-Seq data Analysis 27
Supplemental tables 30

Supplemental figures and legends

Figure S1

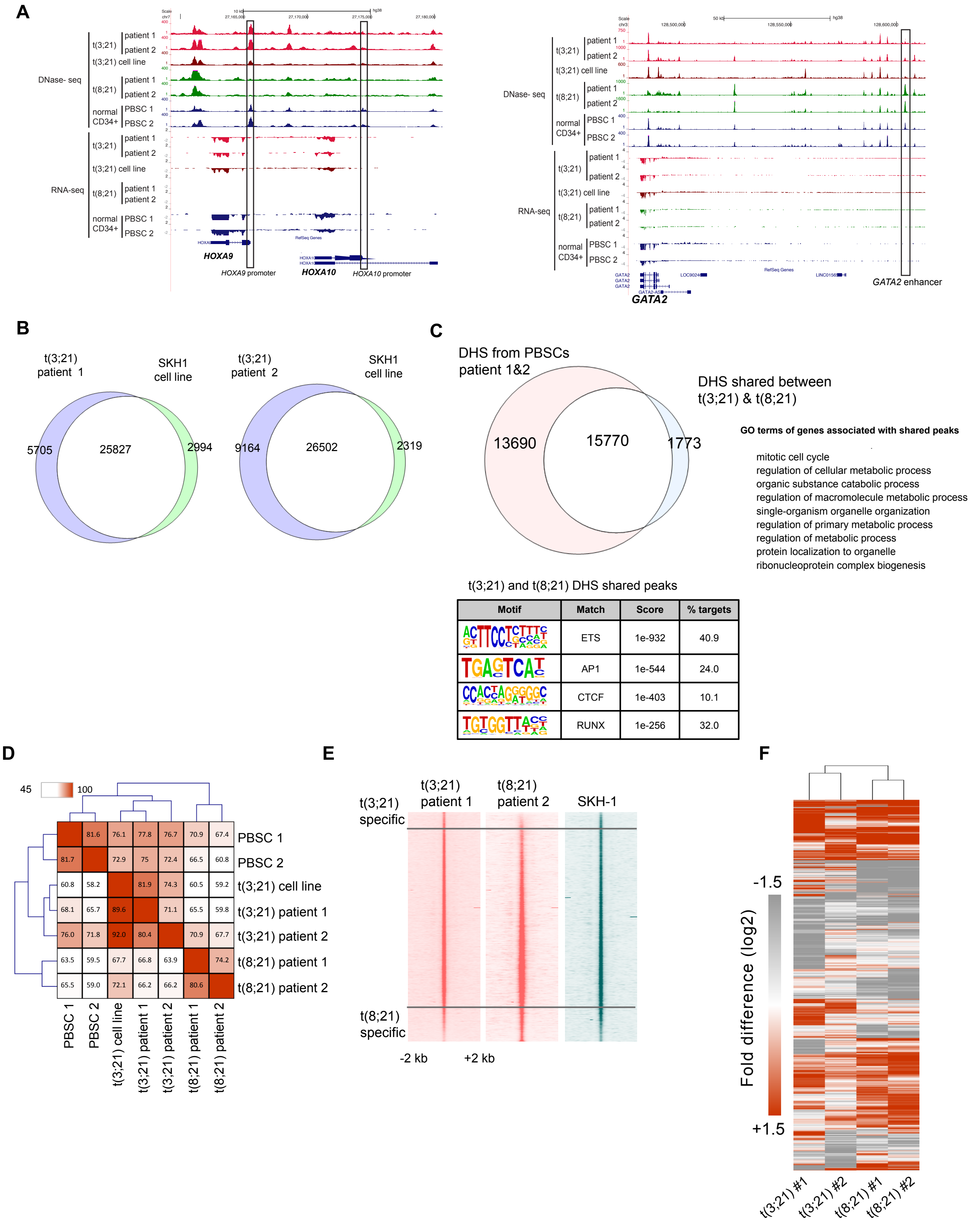

Supplemental Figure 1 (related to Figure 1)

A-B) UCSC genome browser screenshots of DNase-Seq and the corresponding RNA-Seq aligned reads from two patients with t(3;21) AML, two patients with t(8;21) AML, the t(3;21) cell line SKH-1, and normal CD34+ PBSCs. The regions depicted here span the *HOXA9/HOXA10* loci and the *GATA2* locus. The HOXA9 and HOXA10 loci include DHSs present in t(3;21) cells but not in t(8;21), whereas the *GATA2* encompass DHSs present in t(8;21) cells but not t(3;21) cells.

B) Venn diagram showing DHS overlap between t(3;21) patient 1 and SKH-1 cells, and also between t(3;21) patient 2 and SKH-1. Labels represent numbers of DHSs.

C) Venn diagram overlapping the DHS shared between t(3;21) and t(8;21) patient cells with those of CD34+ cells . Right pane: GO terms of genes associated with shared peaks. Bottom panel: enriched binding motifs in shared peaks.

D) Percentage overlap of DHS peaks in a pairwise comparison between each sample for the t(3;21) and t(8;21) patients and two samples of normal CD34+PBSCs. The clustering analysis defined three major clusters corresponding to (i) CD34+ PBSCs, (ii) t(3;21) patient samples and cell line, and (iii) t(8;21) patient samples.

E) DNase-Seq profiles spanning 4 kb windows for t(3;21) patient 1, t(8;21) patient 2, and SKH-1 cells. Peaks are ranked from top to bottom in order of increasing relative DNA sequence tag count for peaks identified in t(8;21) patient 2 relative to t(3;21) patient 1. The horizontal lines indicate the thresholds for peaks defined as either t(3;21) (top) or as t(8;21)-specific (bottom).

F) Hierarchical clustering of each RNA-Seq experiment by gene expression (fold difference in comparison with normal CD34+ PBSCs (expressed as log2 values). Clustering is based on the differential expression of genes in each patient sample, and reveals that patients with t(3;21) leukemia cluster apart from patients with t(8;21) leukemia.

Figure S2

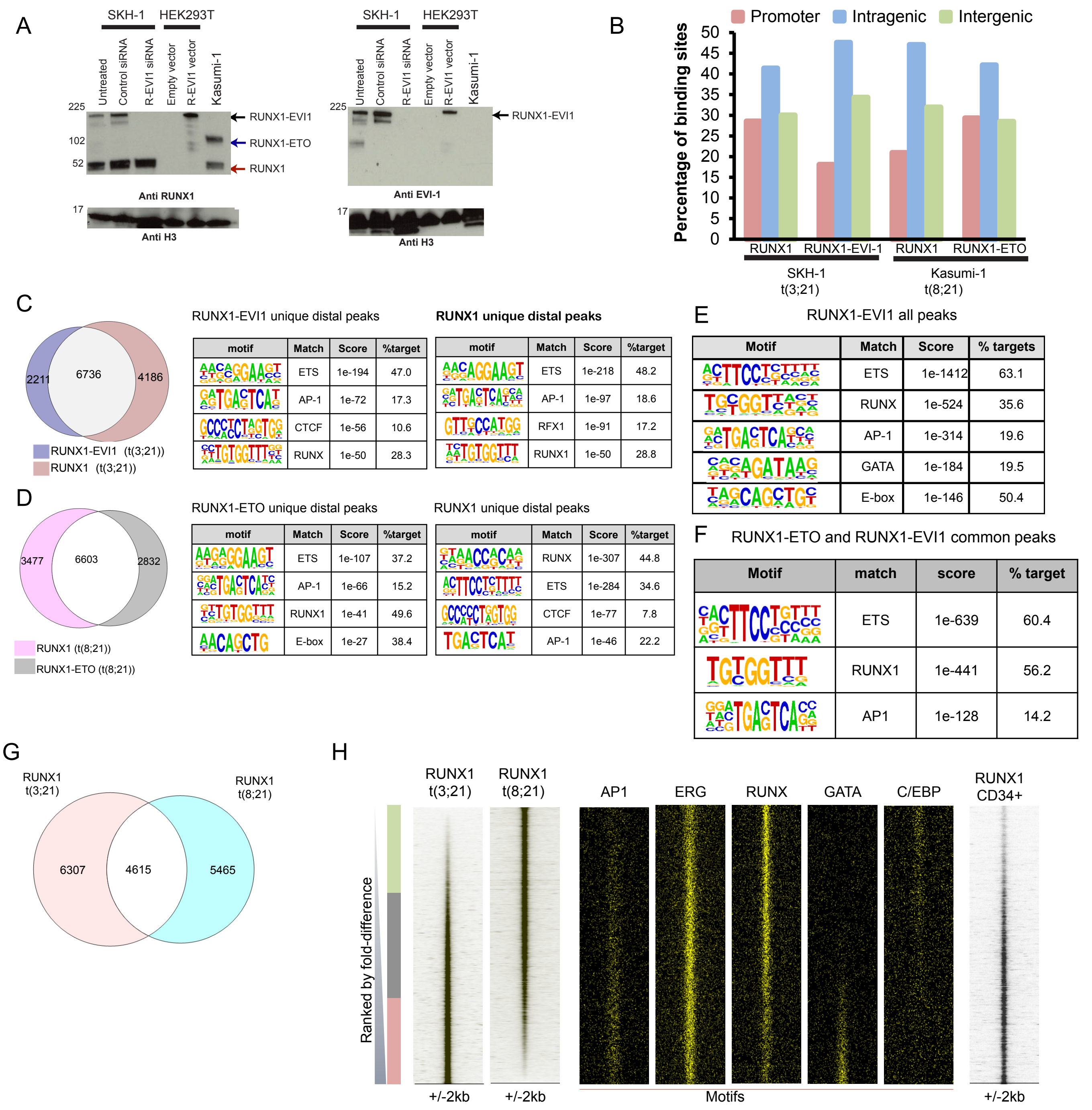

Supplemental Figure 2 (related to Figure 2)

A) Western blot analyses of nuclear extracts from: t(3;21) SKH-1 cells (untreated or transfected with control siRNA or RUNX1-EVI1 siRNA), HEK293T cells (transfected with either empty vector or RUNX1-EVI1 vector as size control), and t(8;21) Kasumi-1 cells. Sizes in kDalton are shown on the left of the blot. Western were blots probed with either an anti-EVI1 or an anti-RUNX1 (N-terminal epitope antibody). Anti-EVI1 and anti-RUNX1 (N-terminal epitope) antibody labels a RUNX1-EVI1 specific band (black arrow). This band is absent in SKH-1 transfected with a RUNX1-EVI1 specific siRNA and is present in HEK293T cells only when transfected with a RUNX1-EVI1 plasmid. In Kasumi-1 nuclear extract, EVI1 antibody does not detect anything, whilst the N-terminal RUNX1 antibody detects RUNX1-ETO (which

is smaller than RUNX1-EVI1) (blue arrow). The 52 kDa band is wild type RUNX1 (red arrow). Anti-H3 antibody was used as a loading control.

B) Percentage of binding sites within promoter, intragenic and intergenic region for RUNX1 and EVI1 (RUNX1-EVI1) ChIP-Seq in t(3;21) SKH-1 and RUNX1 and ETO (RUNX1-ETO) ChIP-Seq in t(8;21) Kasumi-1 cells.

C-D) Venn diagrams of peak overlap between ChIP-seq experiments with tables showing de novo motif analyses in distal peaks unique to each ChIP-Seq data set: C) RUNX1-EVI1 vs RUNX1 in t(3;21) SKH-1; D) RUNX1 vs RUNX1-ETO in t(8;21) Kasumi-1.

E) De novo motif analyses in all RUNX1-EVI1 binding sites using HOMER

G) Overlap between RUNX1 peaks between t(3;21) and t(8;21) cells.

H) RUNX1 peaks in t(3;21) cells were ranked according to tag count and RUNX1 peaks from t(8;21) were plotted alongside, together with the motifs for the indicated transcription factors on the right. To the far right RUNX1 peaks in normal CD34+ peripheral blood stem cells are shown..

Figure S3

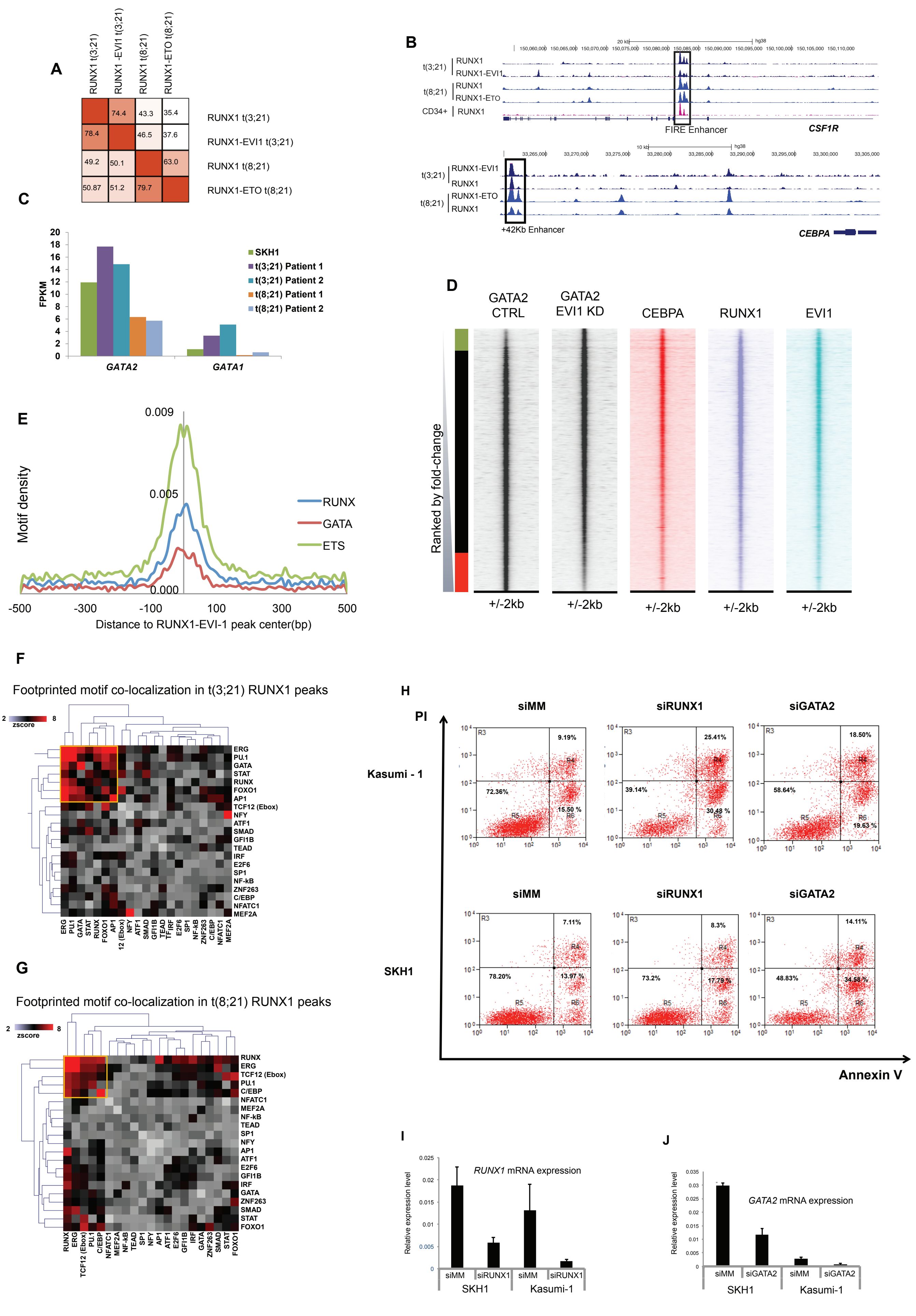

Supplemental Figure 3 (related to Figure 3)

A) Percentage of binding sites within promoter, intragenic and intergenic regions for RUNX1 and EVI1 (RUNX1-EVI1) ChIP-Seq in t(3;21) SKH1 cells and RUNX1 and ETO (RUNX1-ETO) ChIP-Seq in t(8;21) Kasumi-1 cells.

B) UCSC browser screen shot of the *CSF1R* locus. ChIP-Seq experiments were performed measuring RUNX1 and RUNX1-EVI1 binding in t(3;21) SKH-1 cells, RUNX1 and RUNX1-ETO binding in t(8;21) Kasumi-1 cells, and RUNX1 binding in normal CD34+ PBSC. The enhancers at *CSF1R* bind RUNX1 in both t(3;21) and t(8;21) cells, as well as RUNX1-EVI1 or RUNX1-ETO, respectively.

C) Expression of GATA2 and GATA1 based on FPKM values from RNA-Seq in SKH-1 (average of independent replicates), t(3;21) and t(8;21) patients (two patients for each CBF leukemia).

D) ChIP-Seq profiles spanning 4 kb windows for GATA2 ChIP-Seq peaks present in SKH-1 cells after either control siRNA or RUNX1-EVI1 siRNA treatment. Peaks are ranked from top to bottom in order of increasing relative DNA sequence tag count for peaks identified after control siRNA or RUNX1-EVI1 siRNA treatment in SKH-1. The boxes indicate GATA2 binding sites specific to control siRNA treated cells (bottom) or to RUNX1-EVI1 siRNA treated cells (top). To the right are C/EBPα, RUNX1 and RUNX1-EVI1 ChIP-Seq profiles in SKH-1 cells aligned to the same coordinates.

E) Average profiles of the distribution of the indicated binding motifs around the RUNX1-EVI1 binding peak center in a population of sequences bound by RUNX1-EVI1, RUNX1 and GATA2.

F, G) Bootstrap sampling analyses of DNase I footprinted motifs in RUNX1 binding sites from t(3;21) or t(8;21) leukemia. RUNX1 binding sites from ChIP-seq in t(3;21) SKH-1 cells (F) and t(8;21) Kasumi-1 cells (G) mapped onto t(3;21) patient 2 or t(8;21) patient 1, respectively. Motif footprinting probabilities from either t(3;21) patient 2 or t(8;21) patient 1 at these sites. The heatmap shows the probability of footprinted motifs co-localizing within a window of 50 bp, at RUNX1 binding sites, in each leukemia, as compared to sampling by chance alone. Enrichment (red) of GATA, AP-1, ERG and PU.1 occupied motifs is found at RUNX1 bound sites in t(3;21) patient samples (F). In contrast, enrichment (red) of RUNX, ERG and CEBP footprinted motifs at RUNX1 bound sites is found in t(8;21) patient samples (G).

H) Representative FACS panels of 3 independent experiments measuring Annexin V/PI staining on Kasumi-1 and SKH-1 cells after 5 days of knockdown with control siRNA (siMM), RUNX1 and GATA2 specific siRNAs in Kasumi-1 cells (upper panels) or SKH-1 cells (lower panels).

I) *RUNX1* mRNA levels after RUNX1 knockdown in the indicated cell types, relative to *GAPDH* expression. Graph of mean and error bars indicate the SD between three independent experiments.

J) *GATA2* mRNA levels after GATA2 knockdown relative to *GAPDH* expression in the indicated cell types. Graph of mean and error bars indicate the SD between three independent experiments.

Figure S4

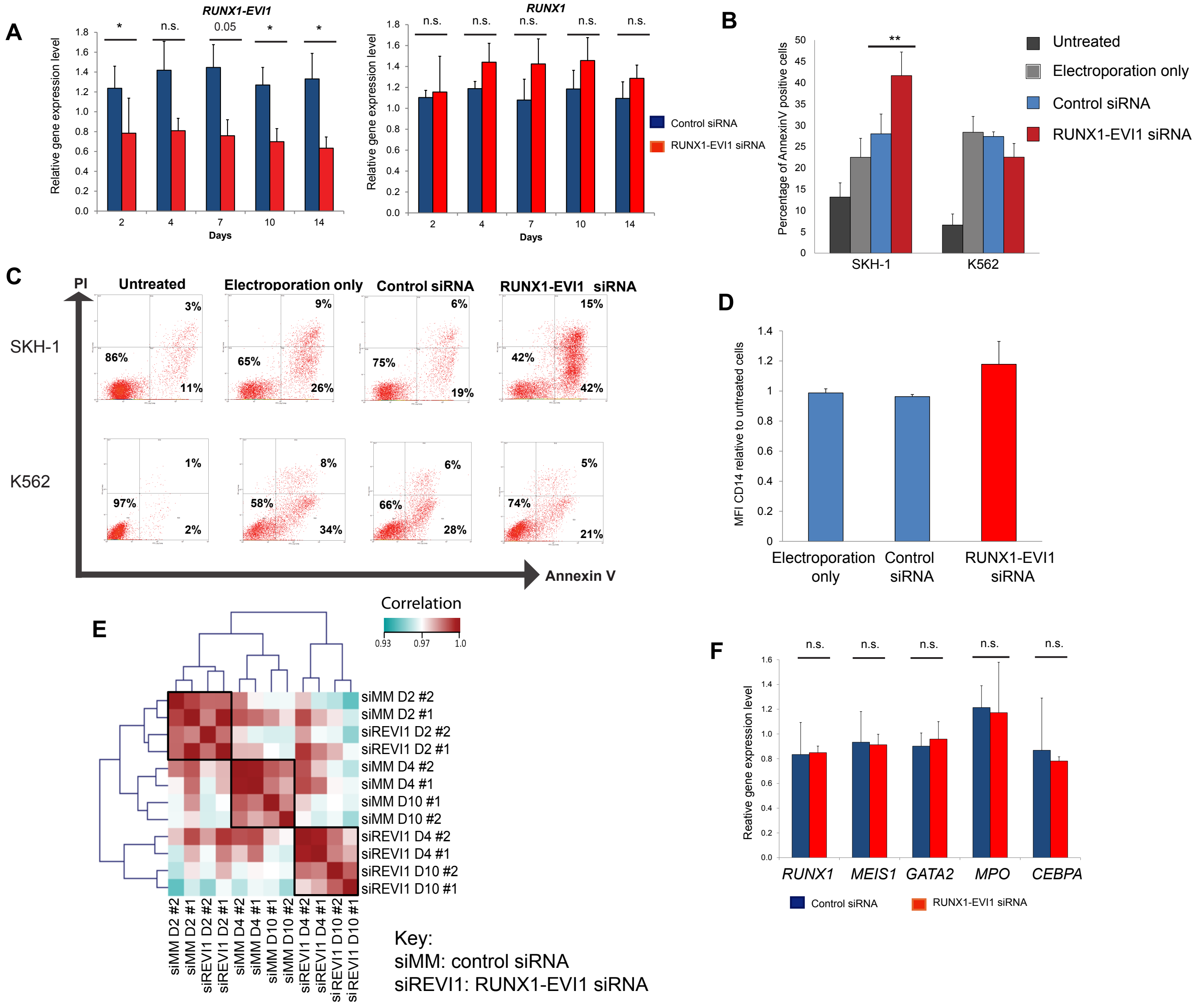

Supplemental Figure 4 (related to Figure 4)

A) *RUNX1-EVI1* mRNA decreases in SKH-1 cells treated with RUNX1-EVI1 siRNA, as compared to control siRNA treatment. *RUNX1* mRNA levels are unaffected by RUNX1-EVI1 siRNA as compared to control siRNA. RT-qPCR showing mRNA levels relative to GAPDH and normalized to untreated cells. *RUNX1-EVI1* or *RUNX1* mRNA levels in SKH-1 transfected with either specific RUNX1-EVI1 siRNA or control siRNA. Graph of mean and SEM of 4 independent experiments. n.s. not significant, * $p<0.05$ by unpaired t-test.

B-C) Treatment with RUNX1-EVI1 siRNA results in increased apoptosis of SKH-1 but not K562 cells. Annexin V FITC and PI staining in SKH-1 and K562 cells either untreated or

after 14 days with electroporation alone or, either, control siRNA or RUNX1-EVI1 siRNA transfection. B) Percentage of Annexin V stained cells. Graph of mean and SEM of at least 3 independent experiments. ** denotes p<0.01 by paired t-test. Representative flow cytometry plots shown in C).

D) CD14 expression on SKH-1 cells is unchanged following RUNX1-EVI1 knockdown. Flow cytometric analysis of t(3;21) SKH-1 cells stained with CD14-FITC: untreated, with either electroporation alone or, control or RUNX1-EVI1 siRNA transfection. MFI (median) of CD14 FITC relative to untreated SKH-1 after14 days of treatment. Graph of mean and SEM of 6 independent experiments.

E) Clustering analysis of RNA-Seq data from of SKH-1 treated with either control siRNA or RUNX1-EVI1 siRNA. RNA was extracted either after 2, 4 or 10 days of treatment as indicated. Hierarchical clustering of Pearson correlation coefficient between two biological replicates (#1 and #2) of each treatment conditions (siMM: control siRNA; siREVI1: RUNX1-EVI1 siRNA) was performed. Independent replicates cluster together but there are also three major clusters: samples from day 2 time point (both control siRNA and RUNX1-EVI1 siRNA treated samples), samples from control siRNA treated samples at day 4 and 10, and samples from RUNX1-EVI1 siRNA treated samples at day 4 and day 10.

F) RT-qPCR measurements of mRNA levels of *MPO*, *RUNX1, CEBPA*, *GATA2* and *MEIS1*, relative to GAPDH and normalized to untreated cells are unchanged in K562 cells after RUNX1-EVI1 siRNA as compared to control siRNA transfection. Graph of mean and SEM of 3 independent experiments. n.s. not significant by unpaired t-test.

Figure S5

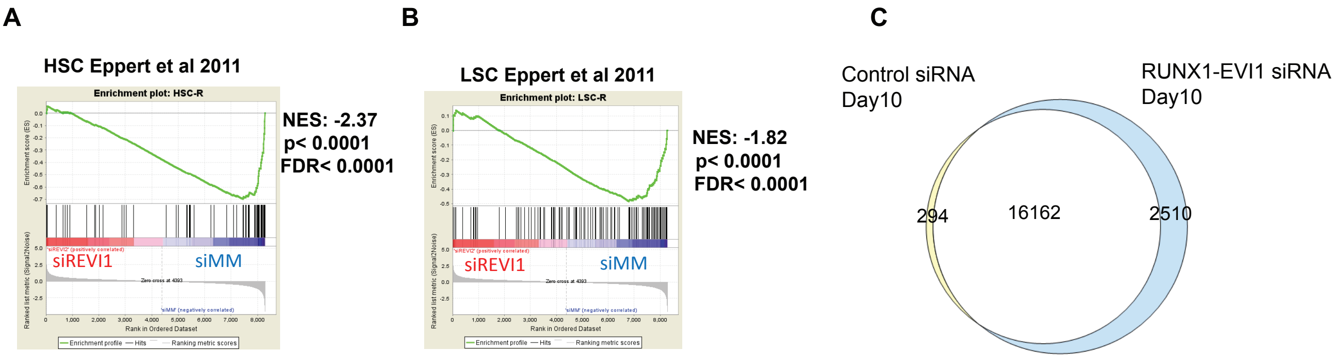

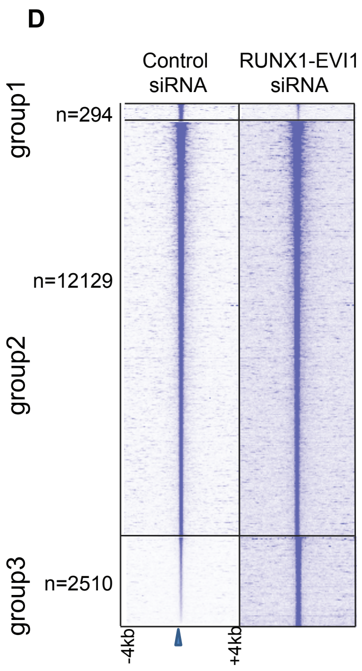

E

De novo motifs (group2)

Motif	match	P-value	%target
	ETS	1e-499	32.8
	AP1	1e-301	20.2
	CTCF	1e-225	13.7
	RUNX	1e-195	32.6
	E-box	1e-46	39.7
	GATA	1e-30	9.0

De novo motifs (group3)

Motif	match	P-value	%target
	ETS	1e-318	42.3
	C/EBP	1e-115	29.5
	RUNX	1e-98	24.5
	AP1	1e-98	18.5
	CTCF	1e-59	9.6

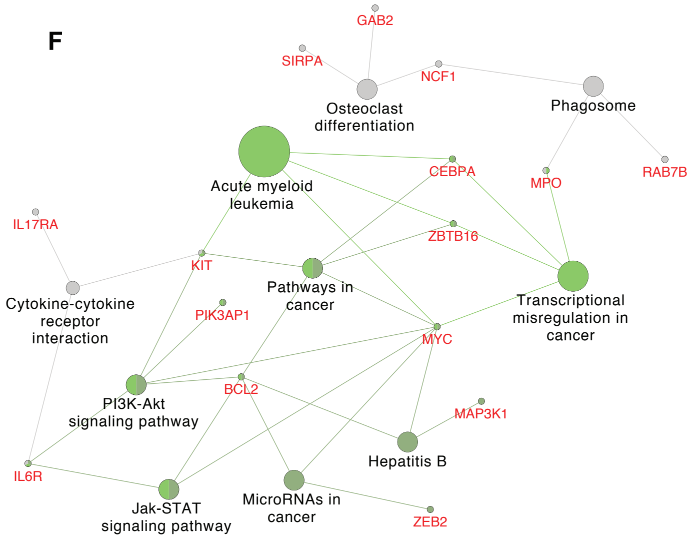

Up-regulated RUNX1-EVI1 target genes responding to RUNX1-EVI1 knock-down

Supplemental Figure 5 (related to Figure 5)

A, B) Gene set enrichment analysis (GSEA) based on RNA-Seq from SKH-1 cells after 10 days of treatment with either RUNX1-EVI1 siRNA (siREVI1) or control siRNA (siMM). Loss of enrichment of gene set associated with either hematopoietic (A) or leukaemic stem cells (LSC, B) when SKH-1 were treated with RUNX1-EVI1 siRNA.

C) Venn diagram of the overlap of DHS peaks showing that knock-down of RUNX1-EVI1 for 10 days leads to the generation of 2510 new DHSs.

D) Heatmap ranking according to sequence tag counts showing DHS profiles after either control siRNA or RUNX1-EVI1 siRNA transfection. Group 1 – 3 identify peaks which are control siRNA-specific, unchanged or RUNX1-EVI1 siRNA specific, respectively.

E) Enriched transcription factor binding motifs in group 2 and 3 peaks defined in (D) highlighting the appearance of C/EBP motifs after RUNX1-EVI1knock-down.

F) KEGG pathway for RUNX1-EVI1 ChIP-seq target genes whose expression is upregulated at least 1.5 fold, between RUNX1-EVI1 siRNA and control siRNA treated cells (10 days of treatment), as identified by RNA-seq.

Figure S6

A

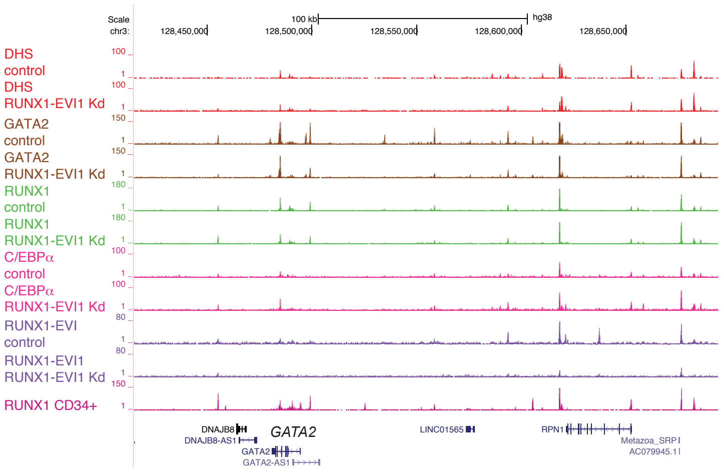

B

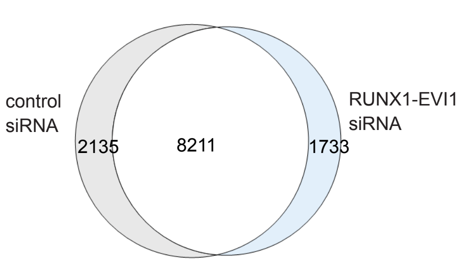

control siRNA specific distal peaks

Motif	match	P-value	%target
	ETS	1e-157	41.7
	RUNX	1e-62	30.9
	AP-1	1e-55	24.2
	**GATA**	1e-26	19.6

shared distal peaks

Motif	match	P-value	%target
	ETS	1e-359	53.2
	RUNX	1e-238	33.7
	AP-1	1e-182	20.6
	SP1	1e-87	15.5

RUNX1-EVI1 siRNA specific distal peaks

Motif	match	P-value	%target
	ETS	1e-154	37.2
	RUNX	1e-71	30.9
	**C/EBP**	1e-49	18.2
	AP-1	1e-43	15.5

C

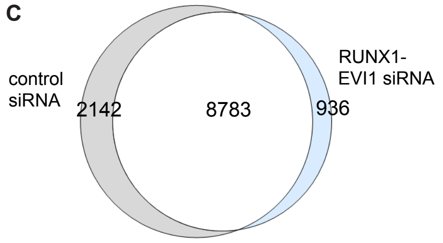

control siRNA specific distal peaks

Motif	Match	P-value	%targets
	ETS	1e-245	61.9
	**GATA**	1e-160	37.3
	RUNX	1e-48	19.7
	AP-1	1e-33	10.1
	E-box	1e-28	57.7

shared distal peaks

Motif	Match	P-value	%targets
	ETS	1e-1049	65.8
	RUNX	1e-383	44.9
	GATA	1e-279	30.8
	AP-1	1e-257	16.7
	E-box	1e-73	29.9

RUNX1-EVI1 siRNA specific distal peaks

Motif	Match	P-value	%targets
	ETS	1e-114	42.8
	**C/EBP**	1e-99	38.3
	RUNX	1e-53	30.7

D

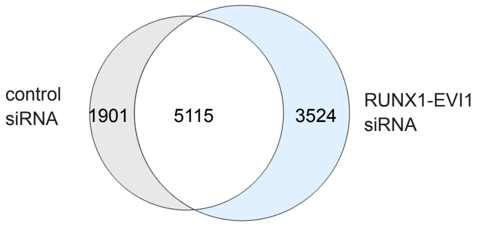

control siRNA specific distal peaks

Motif	match	P-value	%target
	ETS	1e-111	52.6
	AP-1	1e-46	17.5
	RUNX	1e-40	39.4
	**GATA**	1e-15	16.7

shared distal peaks

Motif	match	P-value	%target
	ETS	1e-466	58.7
	RUNX	1e-232	43.5
	AP-1	1e-185	18.3
	C/EBP	1e-79	15.4
	GATA	1e-39	12.1

RUNX1-EVI1 siRNA specific distal peaks

Motif	match	P-value	%target
	ETS	1e-323	39.0
	**C/EBP**	1e-211	28.9
	RUNX	1e-105	30.8
	AP-1	1e-85	15.7

E

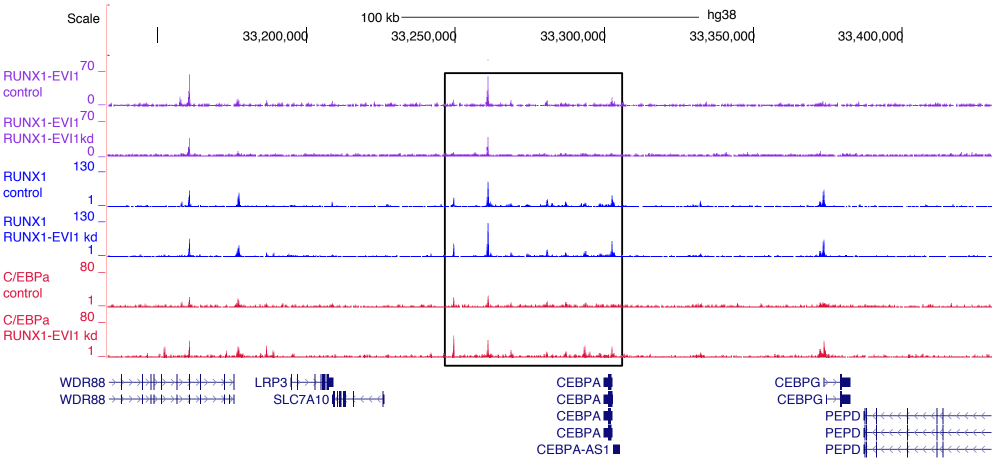

Supplemental Figure 6 (related to Figure 6)

A) UCSC genome browser screen shot of RNA-Seq, DNase-Seq and RUNX1, RUNX1-EVI1 and C/EBPα ChIP-seq 10 days after either control siRNA or RUNX1-EVI1 siRNA treatment at *GATA2.* At *GATA2*, C/EBPα binding increases following RUNX1-EVI1 siRNA transfection.

B) Analyses of RUNX1 ChIP-Seq peaks: GATA motif-containing RUNX1 binding sites are found only in SKH-1 treated with control siRNA whereas CEBP motif-containing RUNX1 ChIP-seq peaks are found only in SKH-1 treated with RUNX1-EVI1 siRNA. Left panel: Venn diagram showing overlap of RUNX1 ChIP-seq peaks in SKH-1 treated with control siRNA with RUNX1 ChIP-seq peaks in SKH-1 treated with RUNX1-EVI1 siRNA (after 10 days treatment). The tables show de novo motif analyses of distal sites found only in SKH-1 cells treated with control siRNA (left), or RUNX1-EVI1 siRNA (right), or found in both control and RUNX1-EVI1 siRNA treated SKH-1 (middle).

C) Analyses of GATA2 ChIP-Seq peaks: Loss of some GATA2 binding sites following RUNX1-EVI1 knockdown in SKH-1 cells. Left panel: Venn diagram showing overlap of GATA2 ChIP-seq peaks in SKH-1 treated with control siRNA with GATA2 ChIP-seq peaks in SKH-1 treated with RUNX1-EVI1 siRNA (after 10 days treatment). The tables shows de novo motif analyses of distal sites found only in SKH-1 treated with control siRNA (left), or RUNX1-EVI1 siRNA (right), or found in both control and RUNX1-EVI1 siRNA treated SKH-1 (middle).

D) Analyses of C/EBPα ChIP-Seq peaks: GATA motif containing sites found in control siRNA but not RUNX1-EVI1 siRNA treated SKH-1 C/EBPα binding sites. Conversely CEBP motif containing sites most commonly found in RUNX1-EVI1 siRNA, but not control siRNA treated SKH-1 C/EBPα binding sites. Left panel: Venn diagram showing overlap of C/EBPα ChIP-seq peaks in SKH-1 treated with control siRNA with C/EBPα ChIP-seq peaks in SKH-1 treated with RUNX1-EVI1 siRNA (after 10 days treatment). The tables show de novo motif analyses of distal sites found only in SKH-1 treated with control siRNA (left), or RUNX1-EVI1 siRNA (right), or found in both control and RUNX1-EVI1 siRNA treated SKH-1 (middle).

(E) UCSC browser screenshot depicting transcription factor binding (RUNX1-EVI1, RUNX1 and C/EBPα) to the regulatory elements of the *CEBPA* locus (boxed in), without (control) and with RUNX1-EVI1 knock-down.

Figure S7

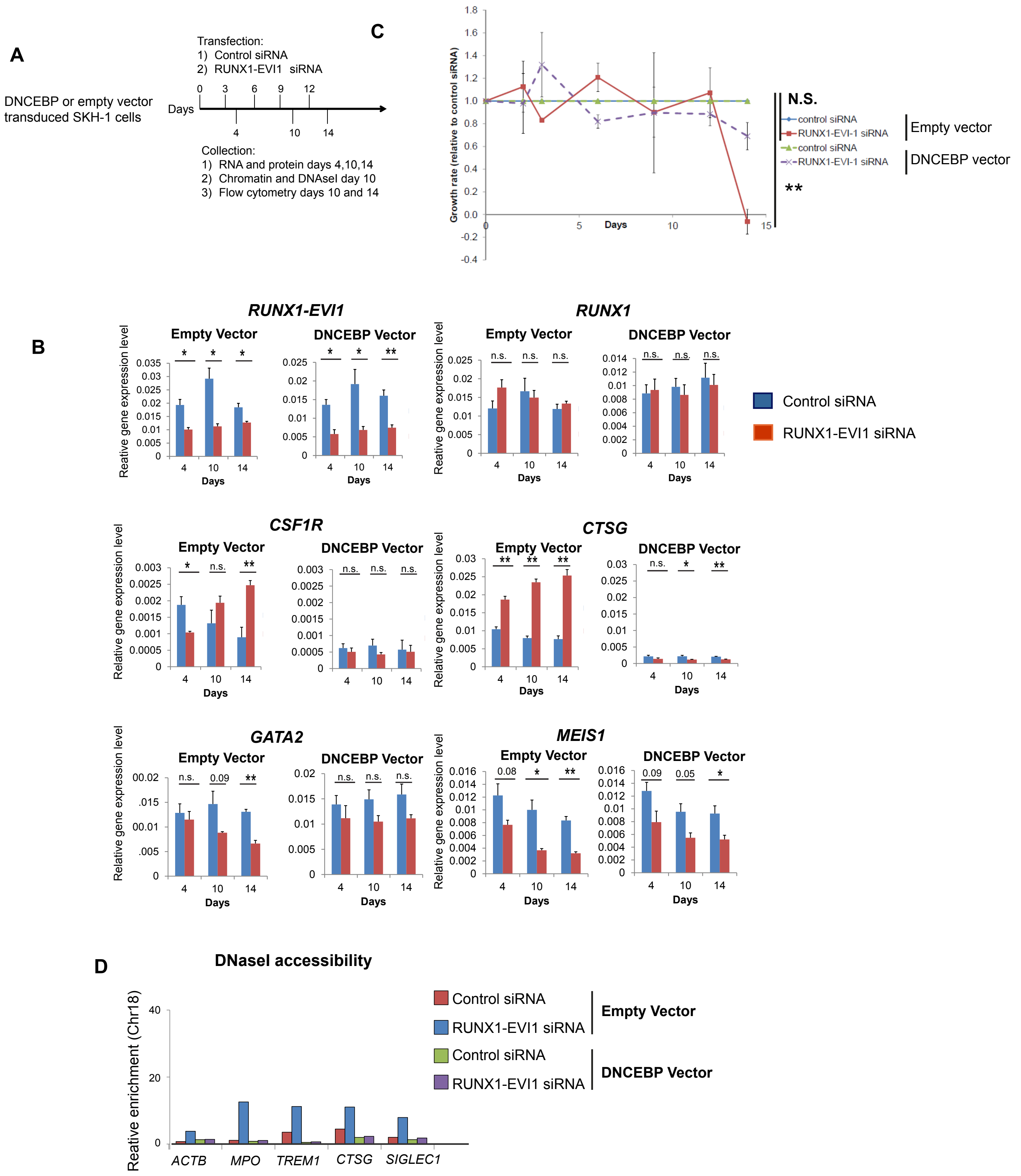

Supplemental Figure 7 (related to Figure 7)

A) Experimental scheme of RUNX1-EVI1 knockdown in either empty vector or DNCEBP vector transduced SKH-1 cells. Transfection with either control siRNA or RUNX1-EVI1 siRNA on days 0, 3, 6, 9 or 12 in both empty vector transduced SKH-1 or DNCEBP transduced SKH-1. Cells collected for RNA, chromatin, DNase I or flow cytometry on designated days.

B) mRNA levels as measured by RT-qPCR, relative to GAPDH after control or RUNX1-EVI1 siRNA transfection (4, 10 or 14 days of treatment). RUNX1-EVI1 or RUNX1 mRNA levels in either empty vector or DNCEBP vector transduced SKH-1. Decrease in *RUNX1-EVI1* mRNA levels in both empty or DNCEBP vector transduced SKH-1transfected with RUNX1-EVI1 siRNA, but *RUNX1* mRNA levels remain the same. *CTSG* and *CSF1R* mRNA levels increase in empty vector transduced SKH-1 following RUNX1-EVI1 knockdown but not in DNCEBP vector transduced SKH-1 treated in an identical manner. *GATA2* and *MEIS1* mRNA levels decrease in empty vector transduced SKH-1 following RUNX1-EVI1 knockdown. *GATA2* and *MEIS1* mRNA levels also decrease in DNCEBP vector transduced SKH-1 following RUNX1-EVI1 knockdown, but this decrease is reduced. n.s. not significant, * $p<0.05$ ** $p<0.01$ by unpaired t-test

C) Growth rates of empty (solid line) or DNCEBP vector (dashed line) transduced SKH-1 cells, transfected with RUNX1-EVI1 siRNA, relative to control siRNA treatment. Mean and SEM of 3 independent experiments. ** denotes $p<0.01$ by paired t-test between control and RUNX1-EVI1 siRNA transfected empty vector transduced SKH-1 cells, after 14 days of siRNA treatment. Differences between growth rates of DNCEBP transduced SKH-1 after RUNX1-EVI1 siRNA and control cells by paired t-test. (n.s.): not significant.

D) DNase I accessibility measurement using qPCR at the *MPO* and *SIGLEC1* enhancer, and the *TREM1* and *CTSG* promoter. DNase I digests were performed on empty vector and DNCEBP vector transduced SKH-1 cells following either control or RUNX1-EVI1 siRNA transfection. DNase I accessibility increases in empty vector transduced SKH-1 following RUNX1-EVI1 knockdown, but not in DNCEBP vector transduced SKH-1 cells treated identically. Primers amplifying sequences within the *ACTB* gene body are shown as negative control. Enrichment was normalized relative to chromosome 18. A 2nd independent experiment is shown in the main figure 7H.

Supplemental Experimental Procedures

Cell line culture

Cells were maintained in a humidified incubator at 37°C with 5% CO2. t(3;21) SKH-1 and K562 cells were cultured in RPMI medium with 10% fetal calf serum (FCS) supplemented with glutamine and penicillin/streptomycin. t(8;21) Kasumi-1 cells were cultured in RPMI with 15% FCS supplemented with glutamine and penicillin/streptomycin. HEK293T and HeLa cells were cultured in Dulbecco Modified Eagle Media (DMEM) with 10% FCS supplemented with glutamine and penicillin/streptomycin.

Purification of blood samples from patients with AML

Blood from t(3;21) patient 1 was diluted 1:1 with PBS and layered onto density gradient medium (Lymphoprep Stem Cell technology, USA). The blood-PBS-lymphoprep mix was subsequently centrifuged at 592xg (acceleration setting 4, no brakes). After centrifugation, the mixture had separated into two phases with the mononuclear cells separated into a layer in between. This middle layer was then isolated and incubated with CD34+ microbeads (Miltenyi-Biotech, USA), according to the manufacturer instructions. The CD34+ expression on this fraction was confirmed by flow cytometry before either immediately use for DNase I hypersensitivity site mapping or RNA extraction by Trizol, as described below.

Cells from t(3;21) patient 2 was previously isolated by density gradient medium and cryopreserved at the Erasmus University Medical Centre, Netherlands. CD34+ cells were thawed with pre-warmed RPMI-1640 +10%FCS. After centrifugation, the cell pellet was re-suspended in 750 µl MACS buffer (PBS with 0.5% BSA and 2 mM EDTA) and 35 µl CD34-PE, with a separate sample stained with IgG PE as an isotype control. CD34+ cells were isolated by FACS using a MoFlo Astrios (Beckman Coulter, USA) and were then directly used for DNase I hypersensitivity site mapping or underwent RNA extraction by Trizol, as described below.

Purification of CD34+ mobilized peripheral blood stem cells

CD34+ peripheral blood stem cells (PBSCs) from healthy adults identified by the NHS Blood and Transplant service (NHSBT) were mobilized into the peripheral circulation by administrating donors with pegylated G-CSF (trade name: Lenograstim, Chugai Pharmaceuticals, Japan). Cells were harvested from the patients by apheresis and stored by NHSBT in liquid nitrogen. Cryopreserved cells were thawed at 37ºC using a waterbath and eluted from storage bag with a PBS/Glucose/Citrate solution (0.09% glucose + 3.3% FCS + 1mM sodium citrate). After centrifugation at 300xg for 5 minutes, the cell pellet was treated with DNase I (Roche, Switzerland) at 0.6mg/ml concentration in PBS/Glucose/Magnesium/Calcium solution (PBS+0.5 mM $MgCl_2$ + 1.2 mM $CaCl_2$) + 1 % FCS + 0.1 % glucose + 2 mM $MgCl_2$) for 5 minutes at room temperature. Following DNase I treatment the cells were once again diluted with PBS/Glucose/Citrate solution and the mononuclear isolated by density gradient medium and CD34+ beads separation by MACS columns, as described above.

siRNA mediated depletion of RUNX1-EVI1, RUNX1 and GATA2

1x10^7 Kasumi-1 or SKH1 cells were electroporated using a EPI 3500 (Fischer, Germany) electroporation at 350v, 10ms. siRNA sequences (Axolabs, Germany) specific for the translocation breakpoint of Runx1-EVI1 were 5'-GAACCUCGAAAUAAUGAGUGU-3' (sense) and 5'-ACUCAUUAUUUCGAGGUUCUC-3' (antisense). Control siRNA was 5'-CCUCGAAUUCGUUCUGAGAAG-3' (sense) with 5'-UC UCAGAACGAAUUCGAGGUU-3' (antisense). siRNA was used at 200 nM. GATA2 siRNAs (3) were: GATA2 ID 2624, Trilencer-27 Human siRNA, Origene Technologies, Inc. and RUNX1 siRNAs (4) were: ON-TARGETplus RUNX1 siRNA, Dharmacon. After electroporation, the cells remained in their cuvettes for 5 minutes before being directly added to RPMI-1640 with 10% FCS, supplemented with penicillin/streptomycin and glutamine at a concentration of 0.5 x10^6 cells per ml and returned to an incubator kept at 37°C and 5% CO_2.

RNA extraction

Pelleted cells from primary patient material were lysed by adding 1ml Trizol ™ (Life Technologies, US). 200 µl of chloroform was added and the mixture was manually shaken for 15 seconds. The mixture was incubated at room temperature for 3 minutes. The mixture was centrifuged at 12000 x g for 15 minutes at 4°C. The top clear aqueous phase was removed and placed in a fresh tube. 0.5 ml of 100% isopropanol was added to the isolated aqueous phase and incubated at room temperature for 10 minutes which was then transferred to a RNeasy MinElute column (Qiagen, USA) and centrifuged for 15 s at 8000xg. 350 µl of RWI buffer from the RNeasy Kit was added to the column and centrifuged for 15 s at 8000xg. 10 µl DNase I and 70µl RDD buffer (Qiagen, USA) were mixed and added to the column and incubated for 15 minutes at room temperature. Afterwards, 350 µl of RWI buffer from RNeasy Kit (Qiagen, USA) was added to the column and centrifuged for 15s at 8000xg. Following this 500 µl of RPE buffer was added and centrifuged for 15 s at 8000xg. The column was washed with 500 µl 80% ethanol and centrifuged at 2 minutes at 8000xg. The column was dried by centrifuged at 5 minutes at 8000xg. RNA was eluted from the column by adding 12 µl of water to the column followed by centrifugation at 5 minutes at 8000xg.

RNA was isolated from SKH-1 cells by Trizol™ (Life Technologies, US) as by manufacturer's instructions. At the last step of the protocol RNA was resuspended in 17 µl of RNAse free water to which was added 2 µl of 10x buffer supplied with the Ambion Turbo DNase I (Thermos Scientific, USA), of which 1 µl was added. All of which was incubated at 37ºC for 30 minutes. The RNA solution was then purified using a Nucleospin RNA clean up column (Machery Nagel, France), according to their instructions. The quality of RNA from all methods was assessed using a spectrophotometer, by the ratio of the absorbance at 260 nM and 280 nM wavelengths. RNA has a greater absorbance in the 260 nM wavelength, Eukaryotic Total RNA PICO Bioanalyser chip (Agilent technologies, USA) allows visualisation of the size of the RNA molecules and thus, demonstrates whether the sample is degraded or not.

RNA Seq libraries

RNA-Seq libraries were prepared with a Total RNA Ribo-zero library preparation kit (with ribosomal RNA depletion) (Illumina, USA) according to manufacturer's instructions with the following alterations: 15 cycles of PCR was undertaken to amplify the library and adaptors for multiplexing were used at a 1:4 dilution. Library quality was checked by running the samples on a Bioanalyser and libraries were quantified using a Kapa library quantification kit

(Kapa Biosystems, USA) and run in a pool of eight indexed libraries in two lane of a HiSeq 2500 (Illumina, USA) using rapid run chemistry with 100bp paired end reads.

cDNA synthesis

1μg RNA was used to make cDNA with 0.5 μg OligoDT primer, Murine Moloney Virus reverse transcriptase and RNase Inhibitor (Promega, USA) according to the manufacturer's protocol.

Real-time polymerase chain reaction

RT-PCR was performed using Sybr Green mix (Applied Biosystems, UK), at 2x dilution. Primers were used at 100 nM final concentration. cDNA was diluted either 1:10 or 1:50 depending on expression levels of targets. A 7900HT system (Applied Biosystems, UK) was used to perform qPCR. Analyses were performed in technical duplicates using a standard curve derived from RNA purified from the untreated cell line (1:10 followed by 1:5 dilutions). Primer sequences are listed in the Appendix.

Dead cell removal and Annexin V/PI staining for flow cytometry

Dead cell removal was performed using negative selection on a MS column following incubation with Dead Cell Removal microbeads (Mitenyi Biotech, USA) as per manufacturer's instructions. Dead cell removal was performed on all samples prior to RNA extraction or DHSs mapping. Annexin V-APC/PI staining (Ebiosciences, USA) or Annexin V-FITC/PI staining (BD Biosciences, USA) was performed according to manufacturer's instructions. Annexin V-APC staining was used for cells that expressed GFP. FACS data were analyzed by Summit 4.3 software (Beckman Coulter).

DNaseI hypersensitivity site mapping

Prior to DNaseI digestion, apoptotic cells were removed using the Dead Cell Removal Kit (Miltenyl Biotech, UK) as per manufacturer's instructions. 3x 10^7 SKH-1 cells were suspended in 1 ml DNase I buffer (0.3M sucrose, 60 mM KCl, 15 mM NaCl, 5 mM MgCl2, 10 mM Tris pH7.4). Digestion on 4.5×10^6 cells was performed with DNase I (Worthington, DPPF grade) at 80 units/ml in DNase I buffer with 0.4% NP-40 and 2 mM CaCl2 at 22ºC for 3 minutes. The reaction was stopped with cell lysis buffer (0.3M NaAcetate, 10mM EDTA pH 7.4, 1% SDS) with 1mg/ml Proteinase K and incubated at 45ºC overnight.

For DHS mapping in CD34+ purified t(3;21) patient cells and in SKH-1 transfected with siRNA, lower cell numbers were available and therefore the DNase I concentrations were reduced according to the cell numbers available.

The digested DNase I material was treated with RNAse A (Sigma Aldrich, Germany) at a final concentration of 100 μg/ml at 37ºC for 1 hr. Genomic DNA was extracted using phenol/chloroform method: an equal volume of phenol was added to the reaction and placed on a rotator wheel for 45 minutes. This was centrifuged for 5 minutes at 16000 x g at room temperature. The top layer was transferred to a new tube and the process was repeated sequentially with phenol/chloroform and chloroform. After purification by chloroform extraction, genomic DNA was precipitated with ethanol. This was pelleted by centrifugation for 5 minutes, at 16000 x g at 4°C. The pellet was resuspended with 70% ethanol and centrifugation for 5 minutes, at 16000 x g at 4°C. The pellet was air-dried and dissolved by Tris-EDTA (40 mM Tris Acetate 1 mM EDTA).

Digestion was checked visually by running the samples on a 0.7% agarose gel and by RT-PCR evaluating the ratio of open (TBP promoter) to closed regions of DNA (chromosome 18) and active gene body (beta-actin) to prevent selection of over digested samples (primers in Table S5B). Subsequently, between 2 to 10 µg of DNase I-digested DNA (depending on material available) were run on a 1.2% agarose gel for selection of shorter fragments to increase the fraction of fragments captured from DHSs. Prior to loading on gel, the purified DNA was treated again with RNAse A (Sigma Aldrich, USA) at a final concentration of 100 µg/ml at 37ºC for 1 hr. 50-300 bp fragments were isolated and purified from the gel using a MinElute gel extraction kit (Qiagen, USA) as per manufacturer's instructions and validated by qPCR as before (Figure 2-1 B-C). Following this, the size selected sample was validated again by RT-PCR, this time using shorter amplicons to enable detection of the shorter fragments enriched by the size selection process.

Library production of DNase I material for high throughput sequencing
After size selection, a library was prepared using Tru-seq DNA sample preparation kit (Illumina, USA) or MicroPlex library preparation kit v2 (Diagenode, Belgium) as per manufacturer's protocol. After PCR a final size selection step was performed by running the library on 1.5% TAE gel, followed by excision of 190-250 bp sized gel fragment. The library was purified from the gel using a MinElute gel extraction kit (Qiagen, USA).

The quality of the libraries was assessed on an Agilent 2100 Bioanalyser. Libraries were subsequently run on two lanes of an Illumina HiSeq 2500 flow-cell for transcription factor footprinting, or as part of 12 indexed libraries in one lane of a NextSeq500 (Illumina, USA) for DHS mapping alone.

ChIP-qPCR and ChIP-Seq library preparation

Double cross-linking
A double cross-linking technique was used to optimize the efficiency of transcription factor chromatin immunoprecipitation (ChIP). $2x10^7$ cells were washed thrice in PBS. Di(N-succinimidyl) glutarate (DSG) (Sigma-Aldrich, Germany) at 850 µg/ml was added to 2x106 cells per ml and were incubated for forty-five minutes. Cells were washed four times and fixed with 1% formaldehyde (Pierce, Thermos Scientific, USA) for ten minutes. Glycine to produce a final concentration of 100mM was added to stop the reaction. The pellet was washed again with PBS. Buffer A (HEPES pH 7.9 10 mM, EDTA 10 mM, EGTA 0.5 mM, Triton x100 0.25%, complete mini protease inhibitor cocktail (PIC) 1x (Sigma-Aldrich, Germany) was added for 10 mins at 4°C and removed by centrifugation at 500 g for 5 minutes. This was repeated with buffer B (HEPES pH 7.9 10 mM, EDTA 1 mM, EGTA 0.5 mM, Triton x100 0.01%, PIC 1x). The residual nuclei were then spun down at 16000 x g at 4°C for 5 minutes and aliquoted at $2x10^7$ cells for 4 immunoprecipitations.

Chromatin immunoprecipitation (ChIP)
Each aliquot of $2x10^7$ cells was re-suspended in 600 µl of sonication buffer (Tris-HCL pH 8 25 mM, NaCL 150 mM, EDTA 2 mM, Triton 100x 1%, SDS 0.25%, Protease inhibitor cocktail (PIC) 1x). 300 µl of nuclei in sonication buffer was placed in each polystyrene tube and sonicated at 75% amplitude, 26 cycles: 30s on and 30s off per cycle (Q800, Active Motif, USA). Subsequently, 1.2ml of dilution buffer (Tris-HCL pH8 25 mM, NaCL 150 mM, EDTA 2 mM, Triton 100x 1%, glycerol 7.5%, PIC 1x) was added to the pooled post sonication

material. This was divided equally between four immunoprecipitations (with 5% of input taken for validation).

15 µl protein G beads (Diagenode, Belgium) were washed twice with 500 µl of 50 mM citrate phosphate buffer and once with 100 mM sodium phosphate). 2 µg antibody (EVI1, C50E12, Cell Signalling, lot 3; or RUNX1, Ab23980, Abcam lot 144722) or 4µg antibody (C/EBPα, A2814 Santa Cruz) was added to 10 µl 100 mM sodium phosphate, 0.5% BSA and incubated with protein G beads at 4°C for 1 hour. Chromatin was then added to the protein G beads with antibody and returned to 4°C for 4 hours. Unbound chromatin was separated from the beads by magnet and the attached beads were washed by buffer 1 (Tris HCL 20 mM, NaCl 150 mM, EDTA 2 mM, Triton x100 1%, SDS 0.1%), twice with buffer 2 (Tris HCL 20 mM, NaCl 500 mM, EDTA 2 mM, Triton x100 1%, SDS 0.1%), LiCL buffer (Tris HCL 10 mM, LiCl 250 mM, EDTA 1 mM, NP40 0.5%, sodium deoxychlolate 0.5%) and finally twice with wash buffer 4 (Tris HCL pH8, 10 mM, NaCl 50 mM, EDTA 1mM). The column was eluted twice with 50 µl buffer (NaHCO3 100 mM and SDS 1%) and the eluant containing the chromatin was pooled. Crosslinks were reversed by incubating the samples at 65°C overnight in 500 mM NaCl, 500 µg/ml proteinase K. DNA was purified by Ampure beads (Beckman Coulter, USA), as above, with the DNA eluted with 50 µl water. Validation of the ChIP was performed by qPCR using a standard curve of genomic DNA from untreated SKH-1 cells (10ng/ µl followed by serial 1:5 dilutions). The input material was diluted 1:5 with water and qPCR was performed as above with primers listed in SI. Validation was analyzed as a ratio of the qPCR signal from the ChIP material over the input.

Library production of ChIP material for high throughput sequencing
Libraries for high throughput sequencing were prepared using the Tru-seq DNA sample preparation kit (Illumina, USA) or Kapa HyperPrep kit (Kapa Biosystems, USA), as per manufacturer's protocol. 18 cycles of PCR was performed and 200-350bp fragments were size selected by running the samples in an agarose gel. Libraries were purified from the gel using a MinElute Gel extraction kit (Qiagen, USA). Libraries were validated by qPCR, with an analysis of the ChIP signal of a positive control region (e.g. PU.1 3H enhancer) over a negative control region (e.g. *IVL*). Finally, libraries were quantified by Kapa library quantification kit (Kapa Biosystems, USA) and run in a pool of four indexed libraries in one lane of a HiSeq 2500 (Illumina, USA) or 12 indexed libraries in one lane of a NextSeq 500 (Illumina, USA) using 50 cycle single-end reads.

Retroviral production

Lenti- and retroviral transduction

pSIEW DNCEBP vector was generated by cloning the DNCEBP insert into the pHR-cppt-SIEW vector (Bomken et al., 2013). The DNCEBP insert was originally developed by Charles Vinson (NIH, USA) (Krylov et al., 1995). We used the pSIEW vector backbone (Empty and DNCEBP) to produce lentiviral particles. Packaging and envelope genes were on a separate plasmid to prevent further virus particle generation once transduced into the target cell.

Transfection of HEK293T cells for lentiviral production
HEK293T cells were re-plated 24 hour prior to transfection, so that at time of transfection they were 80-90% confluent. On the day of transfection TransIT-293T (Mirius, USA) was brought to room temperature. Trans-IT-293T forms a complex with DNA plasmids to enable

transfection into cells. A DNA mix was made from plasmids (Backbone vector containing transgene 30 µg, and the packaging vectors: Tat 1.2 µg, Rev 1.2 µg, Gag/Pol 1.2 µg, VSV-G 2.4 µg (gift from George Murphy, Boston, USA) (Sommer et al., 2009). For each 15 cm^2 2 ml of Optimem serum free media was mixed with 90 µl of TransIT-293 (Mirius, USA). This was allowed to rest at room temperature for 15 minutes. DNA mix was added to the TransIT-293 mixture and was left at room temperature for a further 15 minutes. Fresh DMEM with 10% FCS supplemented with glutamine and penicillin/streptomycin was exchanged with previous media on the HEK293T plates. The TransIT-293 – DNA mixture was then added drop-wise to the HEK293T plate. The viral supernatant was collected after 48 hours and subsequently every 12 hours for 36 hours.

Virus concentration

The virus concentration technique was the same for all viral particles. Viral supernatant was centrifuged at 1660xg 4°C 15 minutes to pellet cell debris. The supernatant was then filtered through a 0.45µm disc filter.

The viral supernatant was concentrated using a Centricon Plus 70 100 kDa filter (Millipore, USA), using the manufacturers instruction. The column was pre-rinsed with sterile water and the column centrifuged at 2000xg, 25 minutes at 4 degrees. The column was then inverted and the concentrate recovered by centrifugation at 1000xg for 2 minutes.

Lentiviral transduction of SKH-1

SKH-1 were transduced with viral concentrates with polybrene at 8 µg/ml by spinoculation at 1500xg for 2 hours at 32°C in non tissue culture treated plates. The plate was subsequently returned to the incubator overnight and at the next morning the viral media was removed and exchanged with fresh media. Viral transduction was estimated by eGFP percentage by flow cytometry 5 days after viral transduction. Cell sorting by FACS was undertaken by sorting for GFP positive cells.

Antibody staining for flow cytometry

$15x10^4$ were centrifuged at 300xg and washed with MACS buffer. The cell pellet was re-suspended in 50 µl MACS buffer and 2 µl of antibody was added and incubated for 15 minutes at 4ºC in the dark. After incubation, the cells was washed once with MACS buffer before resuspension in 300 µl MACS buffer and analyzed on Cyan ADP (Beckman Coulter, USA). Data was analyzed on Summit 4.3 (Beckman Coulter, USA). Antibodies used in this project are listed below.

Whole cell lysate preparation by RIPA buffer lysis

Whole cell lysate was made by lysing $5x10^6$ cells using RIPA buffer (50 mM Tris pH 7.4, 150 mM NaCl, 0.25% Na-deoxycholate, 1% NP40, 1 mM PMSF, complete mini protease inhibitor cocktail (PIC) 1x (Sigma-Aldrich, Germany)). After incubation on ice for 15 minutes, the sample was sonicated for 1 min using a Bioruptor (Diagenode, Belgium) at 4°C.

Nuclear extract

Nuclear extracts were prepared using a co-immunoprecipitation kit (Active Motif, USA). Protein extracts were quantified using Bradford protein reagent (Bio-Rad, USA) and 595nM absorbance quantified by spectrophotometry. Absolute concentrations were determined using a standard curve from a known concentration of BSA (Pierce, USA).

Western blotting

Cell lysate or nuclear extracts was run on an acrylamide gel and transferred to nitrocellulose membrane. This was probed with the antibodies described in the appendix. Enhanced chemiluminescence by SuperSignal PICO (Thermos Scientific, USA) was used to develop the membrane. Chemiluminescence was detected using either developer or Chemidoc XRS system (BioRad, USA).

Oligonucleotide sequences

**Name**	**Forward**	**Reverse**
**Quantitative PCR Primers used for DNA and RNA amplification**		
Actin (long)	GCAATGATCTGAGGAGGGAAGGG	GTGTCTTTCCTGCCTGAGCTGAC
Actin (short)	GCAATGATCTGAGGAGGGAAGGG	AGCTGTCACATCCAGGGTCCTCA
CEBPA	GAGGGACCGGAGTTATGACA	AGACGCGCACATTCACATT
CEBPB	GACAAGCACAGCGACGAGTA	CTCCAGGTTGCGCATCTT
Chromosome 18 (long)	ACTCCCCTTTCATGCTTCTG	AGGTCCCAGGACATATCCATT
Chromosome 18 (short)	AGGTCCCAGGACATATCCATT	GTTCAAATTGTGTTTTGTGGTTA
CSF1R	GCGGGACTATACCAATCTGC	AGCAGGTCAGGTGCTCACTA
CTSG for ChIP	AGACCGTGTAATCCAAGCCA	TCTCGGCACTGACTTAGCAG
CTSG for DNase I	GGTTTCATCACCCAAGGCTG	TGGCTTGGATTACACGGTCT
CTSG for mRNA	TCCTGGTGCGAGAAGACTTTG	GGTGTTTTCCCGTCTCTGGA
GAPDH	CCTGGCCAAGGTCATCCAT	AGGGGCCATCCACAGTCTT
GATA2	CAGACGAAGGCAACCATTTT	GCTCAGACCACCAAGTCTCC
HOXA9	GTGATGCCATTTGGGCTTAT	GGGGTGAGAGAAGGGAGAAG
IVL	GCCGTGCTTTGGAGTTCTTA	CCTCTGCTGCTGCCACTT
MEIS1	CAGAAAAAGCAGTTGGCACA	GGTCTATCATGGGCTGCACT
MPO for mRNA	CCAACAACATCGACATCTGG	GCTGAACACACCCTCGTTCT
MPO for ChIP	CAACACACTCACACCCCACT	TGGGAACTCTAAGTGGGCAG
PU.1 3H Enh	AACAGGAAGCGCCCAGTCA	TGTGCGGTGCCTGTGGTAAT
RUNX1	CCCTCAGCCTCAGAGTCAGAT	AGGCAATGGATCCCAGGTAT
RUNX1-EVI1	CCACAGAGCCATCAAAATCA	TCTGGCATTTCTTCCAAAGG
SIGLEC1	GTATCAGGGGCTGCTTCCTC	CTGGGTTGGACAGTAGAGCT
TBP promoter (long)	CTGGCGGAAGTGACATTATCAA	GCCAGCGGAAGCGAAGTTA
TBP promoter (short)	CTGGCGGAAGTGACATTATCAA	CCCGACCTCACTGAACCC
TREM1	ACAAGGCACCACAATGACCT	GGCCTCATATCCTGTTGTGC
**siRNA sequences**		
RUNX1-EVI1 (Axolabs, Germany)	GAACCUCGAAAUAAUGAGUGU	ACUCAUUAUUUCGAGGUUCUC
RUNX1 (Dharmacon, ON-TARGETplus)	not available	not available

GATA2 (Origene, SR301743)	not available	not available

Antibodies

Antibodies for probing Western blots		
**Antibody target**	**Company**	**Serial number**
EVI1	Cell Signalling	2593
RUNX1 (C-terminal epitope)	Abcam	23980
RUNX1 (N-terminal epitope)	Cell Signalling	4334
C/EBPα	Abcam	40761
FLAG epitope	Sigma	F3165
GAPDH	Abcam	8245
Anti-Rabbit HRP	Cell signalling	7074
Anti-Mouse HRP	Jackson	115 035 062
**Antibodies for ChIP**		
**Antibody target**	**Company**	**Serial number**
EVI1	Cell Signalling	2593
RUNX1 (C-terminal epitope)	Abcam	23980
C/EBPα	Santa Cruz	A2814
GATA2	R+D	AF2046
**Antibodies for flow cytometry**		
**Antibody target-flurochrome**	**Company**	**Serial number**
CD34-APC	Miltenyl-biotech	10098139
CD34-PE	Mitlenyl-biotech	130081002
CD117-APC (Clone A3C6E21)	Mitlenyl-biotech	130091733
CD11b-APC	Miltenyl-biotech	130091241
CD11b PE (Clone M1/70)	Ebiosciences	120011281
CD14-FITC	Miltenyl-biotech	130080701
CD14-PE (Clone MΦP9)	BD Biosciences	562691
Annexin V-FITC/PI kit	BD Pharmingen	556547
Annexin V-APC/PI kit	Ebiosciences	88-8005-74
IgG FITC	Miltenyl-biotech	130093192
IgG PE	Miltenyl-biotech	130093193
IgG APC	Miltenyl-biotech	130093194

DATA ANALYSIS

ChIP and DNase I sequencing data Analysis

Alignment

Sequences from all ChIP and DNase I sequencing experiments in fastq format were mapped onto the reference human genome version hg38, Genome Reference Consortium GRCh38. The quality control statistics for the samples were obtained using FastQC software (http://www.bioinformatics.babraham.ac.uk/projects/fastqc/). The raw reads were aligned to the reference genome using Bowtie2 (Langmead and Salzberg, 2012). Reads from ChIP-seq data that were uniquely aligned to chromosomal positions were retained and duplicate reads were removed from the aligned data using Picard tools (http://broadinstitute.github.io/picard/). The aligned reads were used to generate density profiles using "genomeCoverageBed" function from bedtools (http://bedtools.readthedocs.org/en/latest/). These tag densities were displayed using the UCSC Genome Browser (Kent et al., 2002). The numbers of aligned reads are listed in Table S5. RUNX/ETO ChIP is the combined of a publically available data downloaded from GEO with accession numbers GSM1113429**,** GSM1113430 (Ben-Ami, et. al, 2013) and GSM1082306 (Wang et. al, 2013).

Peak calling

Regions of enrichment (peaks) of ChIP and DNAse1 sequencing data were identified using DFilter software (Kumar et al., 2013) with recommended parameters (-bs=100 -ks=50 –refine). Peak overlaps, gene annotations were performed using in-house scripts. High confidence ChIP-Seq peaks were defined as those overlapping peaks in the DNase-Seq data. Overlaps between ChIP and DNase I sequencing were defined by requiring the summit of a peak in the ChIP dataset to lie between start and end coordinates of a peak in the DNase I data. Peaks were allocated to genes if located in either their promoters or within the region of 2000 bp downstream and 2000 bp upstream of the transcription start sites (TSS), as intragenic if not in the promoter but within the gene body region, or if intergenic, to the nearest gene located within 100 kb. Overlaps between ChIP-seq peaks were defined by requiring the summits of two peaks to lie within +/-200 bp.

Clustering of ChIP and DNAseI sequencing data

Hierarchical clustering with Euclidean distance and complete linkage clustering was used for clustering of transcription factors based on similar binding patterns of different ChIP-seq data, in SKH-1 cells. The high confidence peaks for all transcriptional factors were intersected and merged when overlapping. The read counts for all union peaks were normalized with regards to total reads depth counts and then Pearson's correlation coefficients were calculated between samples using log2 of the normalized read counts. A correlation matrix was generated and Pearson correlation coefficients are displayed after hierarchical clustering as a heatmap. Colors in the heatmap indicate the strength of association between each pair of transcription factors. Heat maps were generated using Mev

from TM4 microarray software suite (Saeed et al., 2006). Same way was used for DNase-Seq data clustering.

Average tag density profile and heatmap

The tag density and average profiles for Figure 1E were generated by calculating the tag density normalized as coverage per million within 4 kb of the DNAse1 peak summit. The read counts for all union peaks were computed. Coverages were calculated for all union peaks and ranked by log2 fold change. Heatmap images were generated via Java TreeView (http://jtreeview.sourceforge.net/) and average profiles were plotted using R (https://www.r-project.org/).

Motif identification and clustering

De novo motif analysis was performed on peaks using HOMER (Benner et.al 2010). Motif lengths of 6, 8, 10, and 12 bp were identified in within ± 200 bp from the peak summit. The top enriched motifs with a significant p value score were recorded. The annotatePeaks function in HOMER was used to find occurrences of motifs in peaks. In this case we used known motif position weight matrices (PWM) from HOMER database.

Motif clustering

Digital footprinting of t(3;21) AML patients 1 and 2 and t(8;21) AML patients 1 and 2 from DNase I high-depth sequencing data was performed using the Wellington algorithm (Piper et al., 2013) with FDR=0.01. For the heatmap that shows hierarchical clustering of motif occurrences within RUNX1/EVI1 footprints (Figure 3E), a motif positions search was done within peaks that are only footprinted in t(3;21) patients. The distance between the centers of each motif pairs was calculated and the motif frequency was counted if the first motif was within 50bps distance from the second motif. Z-scores were calculated from the mean and standard deviation of motif frequencies observed in random sets using bootstrap analysis. For bootstrapping, peak sets with a population equal to that of the footprinted peaks were randomly obtained from the union of t(3;21), t(8;21) and CD34+ DNase-Seq footprints. Motif search was repeated for each random set and then the mean and the standard deviation for the total motif frequencies of the random peak sets were calculated and compared with the actual motif frequencies to obtain the Z- scores. A matrix was generated and Z scores were displayed after hierarchical clustering as a heat map. Red color means that motifs are overrepresented and grey color indicates that motif is underrepresented. The same procedure was repeated with RUNX1/ETO and RUNX1 peaks (Figure S3 and 3F) that are only footprinted in t(8;21) patients and where motif search was done exactly within the footprint coordinates and the random sets were generated from the total patient’s footprints.

Motif enrichment

To identify motifs (identified by HOMER) that are relatively enriched in the distal transcription factors (TFs) sites of one cell type compared to another or one TF compared to another from same cell type we considered all possible comparisons (Figure 3F), these being TF sites in

(A) which are not shared with each of the other TFs (B). For a given set *j* of TFs, we defined a motif enrichment score (S_{ij}) for motif *i* in peak set *j* as

$$S_{ij}\frac{n_{ij}/M_j}{\sum_j n_{ij}/\sum_j M_j}$$

where n_{ij} is the number of peaks in each subset *j (j=1,2,…,12)* containing motif *i (i=1, 2,….,I), I* is the total number of motifs used in the test, and M_j the total number of peaks in each subset *j (j=1,2,…,30).* A matrix was generated and the motif enrichment scores were displayed as a heatmap after hierarchical clustering with Euclidean distance and complete linkage. The heatmap was generated using Mev from TM4 microarray software suite (Saeed et al., 2006).

RNA-Seq data Analysis

RNA-Seq reads were aligned to the hg38 human genome build using STAR. Separate density profiles for the positive and negative strand were generated for RNA-Seq data. Fragments per Kilobase of transcript per Million mapped reads (FPKM) values for each gene were extracted using Cufflinks and differentially expressed genes were extracted using the limma R package (Ritchie et al., 2015). All genes with p-value ≤ 0.01 were considered and at least 1.5-fold changes between before and after RUNX1/EVI1 knock down. The differentially expressed genes for the AML patients were considered with at least 2-fold changes using the CD34+ PBSC as a control. The numbers of aligned reads are listed in Table S5.

The correlation between any two samples was obtained as the Pearson correlation coefficient of expression values over all genes. A correlation matrix was thus generated for all the samples and hierarchically clustered.

Clustering of gene expression was carried out on signal intensity for all expressed genes and on fold-changes for genes associated with at least a 1.5-fold change. Hierarchical clustering was used with Euclidean distance and average linkage clustering. Heatmaps were generated using Mev (Saeed et al., 2006).

The GSEA software (Subramanian et al., 2005) was used to perform gene set enrichment analysis on group of genes. The normalized enrichment score (NES), the p-value and the FDR q-value are displayed on the enrichment plot.

Gene ontology (GO) analysis was performed using Bingo (Maere et al., 2005) and David online tool at david.abcc.ncifcrf.gov (Huang da et al., 2009) using Hypergeometric for overrepresentation and Benjamini and Hochberg (FDR) correction for multiple testing corrections. KEGG Pathway network analysis was performed using clueGO tools (Bindea et al., 2009) with kappa score = 0.3. The right-sided enrichment (depletion) test based on the hypergeometric distribution was used for terms and groups. Groups were created by iterative merging of initially defined groups based on the kappa score threshold. The relationship between the selected terms is defined based on their shared genes and the final groups are randomly colored where one, two colors or more represents that a gene/term is a member of

one, two or more groups respectively. The size of the nodes reflects the enrichment significance of the terms. The network is laid out using the layout algorithm supported by Cytoscape.

Supplemental References

1. Bindea, G., Mlecnik, B., Hackl, H., Charoentong, P., Tosolini, M., Kirilovsky, A., Fridman, W.H., Pages, F., Trajanoski, Z., and Galon, J. (2009). ClueGO: a Cytoscape plug-in to decipher functionally grouped gene ontology and pathway annotation networks. Bioinformatics *25*, 1091-1093.
2. Ben-Ami O, Friedman D, Leshkowitz D, Goldenberg D et al. Addiction of t(8;21) and inv(16) acute myeloid leukemia to native RUNX1. Cell Rep 2013 Sep 26;4(6):1131-43.
3. Bomken S, Buechler L, Rehe K, Ponthan F, Elder A, Blair H, Bacon CM, Vormoor J, Heidenreich O. Lentiviral marking of patient-derived acute lymphoblastic leukaemic cells allows in vivo tracking of disease progression. Leukemia. 2013 Mar;27(3):718-21.
4. Heinz S, Benner C, Spann N, Bertolino E et al. Simple Combinations of Lineage-Determining Transcription Factors Prime cis-Regulatory Elements Required for Macrophage and B Cell Identities. Mol Cell 2010 May 28;38(4):576-589.
5. Kent WJ, Sugnet CW, Furey TS, Roskin KM, Pringle TH, Zahler AM, Haussler D., The human genome browser at UCSC. Genome Res. 2002 Jun;12(6):996-1006.
6. Kumar V, Muratani M, Rayan NR, Kraus P, Lufkin T, Ng HH and Prabhakar S, Uniform, optimal signal processing of mapped deep-sequencing data, Nature biotechnology, 31, 615-622 (2013)
7. Langmead B, Salzberg S. Fast gapped-read alignment with Bowtie 2. Nature Methods.2012, 9:357-359.
8. Maere, S., Heymans, K., and Kuiper, M. (2005). BiNGO: a Cytoscape plugin to assess overrepresentation of gene ontology categories in biological networks. Bioinformatics *21*, 3448-3449.
9. Piper, J., Elze, M.C., Cauchy, P., Cockerill, P.N., Bonifer, C., and Ott, S. (2013). Wellington: a novel method for the accurate identification of digital genomic footprints from DNase-seq data. Nucleic Acids Res *41*, e201.
10. Ritchie et., al. *limma* powers differential expression analyses for RNA-sequencing and microarray studies. Nucl. Acids Res. *(2015)*
11. Saeed, A.I., Bhagabati, N.K., Braisted, J.C., Liang, W., Sharov, V., Howe, E.A., Li, J., Thiagarajan, M., White, J.A., and Quackenbush, J. (2006). TM4 microarray software suite. Methods Enzymol *411*, 134-193.
12. Subramanian, A., Tamayo, P., Mootha, V.K., Mukherjee, S., Ebert, B.L., Gillette, M.A., Paulovich, A., Pomeroy, S.L., Golub, T.R., Lander, E.S*., et al.* (2005). Gene set enrichment analysis: a knowledge-based approach for interpreting genome-wide expression profiles. Proc Natl Acad Sci U S A *102*, 15545-15550.
13. Sun XJ, Wang Z, Wang L, Jiang Y et al. A stable transcription factor complex nucleated by oligomeric AML1-ETO controls leukaemogenesis. Nature 2013 Aug 1;500(7460):93-7.

14. X Jiao, BT Sherman, R Stephens, MW Baseler, HC Lane, RA Lempicki. DAVID-WS: a stateful web service to facilitate gene/protein list analysis. Bioinformatics (2012) 28 (13): 1805-1806.

Supplemental tables

Table S1 Table S1 is an attached Excel file available via the website.

Title: Gene expression levels in patient cells and cell lines (RNA-Seq). Related to Figures 1 and 4

Sheet 1) RNA-Seq data from CD34+ cells purified from patients with either t(3;21) or t(8;21) AML, and from normal PBSCs. Related to Figure 1.
RNA-Seq values are expressed as FPKM.

Sheet 2) RNA-Seq data from SKH-1 cells following RUNX1-EVI1 siRNA transfection as compared to control siRNA transfection. Related to Figure 4
RNA was extracted after 2, 4 and 10 days of treatment. Values are expressed as FPKM values from RNA-Seq in control siRNA (MM) and RUNX1-EVI1 siRNA (KD) transfected cells. Gene expression fold change (FC) is given following either 2, 4 or 10 days of siRNA transfection.

Table S2 Patient details

Details of patient samples included in this study. t(8;21) patient 1 and 2 were processed before the commencement of this study.

ID	White cell count X 10^9/L	Age	Sex	Stage of treatment	Cytogenetics	Clinical notes	Mutations
t(3;21) # 1	23	44	F	Presentation	46,XX,t(3;21)(q26;q22), der(5)t(5;13)(q2;q3),der(7)t(1;7)(q3;q3[5]/47, idem,+12,+der(21), t(3;21)[4]	Therapy related AML (previous Myelofibrosis and T cell lymphoma)	KRAS
t(3;21) # 2	54	72	M	Presentation	46,XY,t(3;21)(q26;q22), del(12)(p12p13)[20]	RAEB-t	DNMT3, SRSF2
t(8;21) # 1	2.12	45	M	Presentation	46,XY,t(8;21)(q22;q22)	AML	None found
t(8;21) # 2	53	53	M	Presentation	46,XY,t(8;21)(q22;q22)	AML	CBL, FLT3-TKD
PBSC # 1	N/A	51	M	N/A	N/A	Mobilized PBSCs from sibling donor for allogeneic stem cell transplant	N/A
PBSC # 2	N/A	47	F	N/A	N/A	Mobilized PBSCs from autologous donor for stem cell transplant (CNS lymphoma)	N/A

Table S3 DNA Sequencing data and peak numbers of the experiments indicated in the table below (related to Figures 1,2,3, 6)

**DNase-Seq reads alignment and peaks detected in t(3;21) SKH-1 cell line, primary CD34+ AML blasts and normal CD34+ PBSC.**			
**DNase-Seq Dataset**	**Aligned reads**	**Total Peaks**	
CD34 J209 (PBSC 1)	173,261,382	36,041	
CD34 R299 (PBSC 2)	205,284,104	36,088	
t(3;21) SKH1 cell line	348,530,966	28,821	
GT027 (t(3;21) patient 1	407,335,267	31,532	
AML5354 (t(3;21) patient 2	237,196,453	35,666	
H12812 (t(8;21) patient 1	405,680,774	32,262	
H18901 (t(8;21) patient 2	387,658,545	35,052	
**ChIP-seq in untreated t(3;21) SKH-1, and normal CD34+ PBSC** (High confidence peaks are ChIP-seq peaks that overlap with a DHS.)			
**ChIP Dataset**		**Total peaks**	**High Confidence Peaks**
Anti-RUNX1 SKH1		15,609	10,922
Anti-EVI1 SKH1		14.992	8,947
Anti-RUNX1 CD34+PBSC		13,951	11,226
**DNase-Seq alignments from SKH-1 after 10 days treatment of control or RUNX1-EVI1 siRNA**			
**DNase-Seq Dataset**	**Aligned reads**	**Total peaks**	
Control siRNA Day 10	8,531,753	16,456	
RUNX1-EVI1 siRNA Day 10	19,976,087	18,672	
**ChIP-Seq read alignments from SKH-1 after 10 days (D10) treatment of control (MM) or RUNX1-EVI1 siRNA (KD)** (High confidence peaks are ChIP-seq peaks that also coincide with DHSs.)			
**ChIP-seq Dataset**	**Aligned reads**	**Peaks**	**High Confidence Peaks**
Anti-EVI1 D10 MM	20,350,942	12,842	7,252
Anti-EVI1 D10 KD	29,752,295	2,014	1,208
Anti-RUNX1 D10 MM	19,147,420	15,185	10,346
Anti-RUNX1 D10 KD	20,959,942	15,592	9,944
Anti- C/EBPα D10 MM	23,509,134	9,346	7,016
Anti- C/EBPα D10 KD	29,564,903	11,808	8,639
Anti- GATA2 MM	44,761,095	14,640	8,100
Anti- GATA2 KD	22,408,237	13,628	9,791

Table S4 RNA-Seq read alignments of the experiments indicated in the table below (related to Figures 1 and 4)

**RNA-Seq data in t(3;21) SKH-1 cell line, primary CD34+ AML blasts and normal CD34+ PBSC.**	
**RNA-Seq Dataset**	**Aligned reads**
t(3;21) SKH-1 cell line replicate 1	30,334,985
t(3;21) SKH-1 cell line replicate 2	11,898,204
CD34 R454 (PBSC # 1)	16,538,775
CD34 R423 (PBSC # 2)	12,402,996
H12812 (t(8;21) patient 1)	12,347,883
H18901 (t(8;21) patient 2)	11,331,201
GT027 (t(3;21) patient 1)	48,166,460
AML5354 (t(3;21) patient 2)	11,969,780
**RUNX1-EVI1 or control siRNA transfected SKH-1 RNA-Seq data**	
Control siRNA (siMM) or RUNX1-EVI1 siRNA (siREVI1) transfected cells. RNA -Seq in SKH-1 cells after either 2 (D2), 4 (D4) or 10 (D10) days of siRNA treatment. Two biological replicates performed (Rep1 and Rep2).	
**RNA-Seq Dataset**	**Aligned reads**
siMM_D2_Rep1	43,584,690
siMM_D2_Rep2	41,344,409
siMM_D4_Rep1	33,410,586
siMM_D4_Rep2	44,832,782
siMM_D10_Rep1	22,705,489
siMM_D10_Rep2	22,419,529
siREVI1_D2_Rep1	43,782,238
siREVI1_D2_Rep2	23,157,018
siREVI1_D4_Rep1	37,541,312
siREVI1_D4_Rep2	53,187,360
siREVI1_D10_Rep1	17,656,510
siREVI1_D10_Rep2	30,380,957
