# Supplementary material for: RUNX1-ETO and RUNX1-EVI1 Differentially Reprogram the Chromatin Landscape in t(8;21) and t(3;21) AML
Source: Cell Rep. 2017 May 23;19(8):1654–68. doi: 10.1016/j.celrep.2017.05.005 (PMC5457485; doi:10.1016/j.celrep.2017.05.005)
Supplement: Document S2. Article plus Supplemental Information [file mmc3.pdf]

Cell Reports

Article

RUNX1-ETO and RUNX1-EVI1 Differentially Reprogram the Chromatin Landscape in t(8;21) and t(3;21) AML

Graphical Abstract

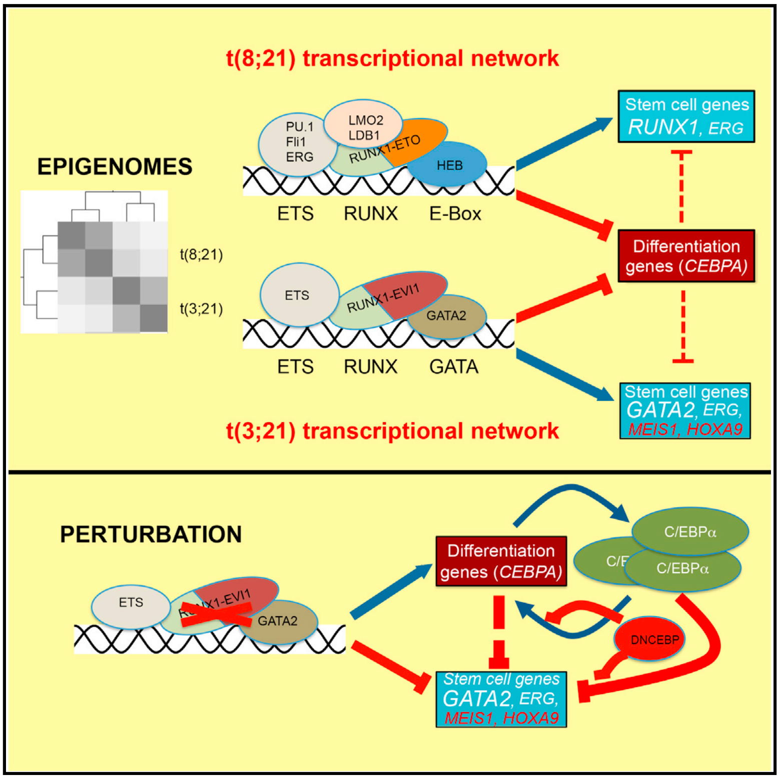

Authors

Justin Loke, Salam A. Assi, Maria Rosaria Imperato, ..., Peter N. Cockerill, Olaf Heidenreich, Constanze Bonifer

Correspondence

c.bonifer@bham.ac.uk

In Brief

Loke et al. compare the regulatory signature of two related types of acute myeloid leukemia, t(8;21) expressing RUNX1-ETO and t(3;21) expressing RUNX1-EVI1. Each fusion protein displays a distinct binding pattern and cooperates with different transcription factors to impact the epigenome, but both downregulate the myeloid differentiation regulator C/EBPα.

Highlights

- t(8;21) and t(3;21) AML are regulated by unique gene regulatory networks
- RUNX1-ETO and RUNX1-EVI1 have different binding patterns and binding partners
- Depletion of each fusion protein initiates C/EBPα-dependent myeloid differentiation
- t(8;21) AML survival depends on RUNX1, whereas t(3;21) AML survival requires GATA2

Accession Numbers

GSE87286

CrossMark

Loke et al., 2017, Cell Reports *19*, 1654–1668
May 23, 2017 © 2017 The Author(s).
http://dx.doi.org/10.1016/j.celrep.2017.05.005

CellPress

OPEN ACCESS
CellPress

Cell Reports
Article

RUNX1-ETO and RUNX1-EVI1 Differentially Reprogram the Chromatin Landscape in t(8;21) and t(3;21) AML

Justin Loke,[1,4] Salam A. Assi,[1,4] Maria Rosaria Imperato,[1] Anetta Ptasinska,[1] Pierre Cauchy,[1] Yura Grabovska,[2] Natalia Martinez Soria,[2] Manoj Raghavan,[1] H. Ruud Delwel,[3] Peter N. Cockerill,[1] Olaf Heidenreich,[2] and Constanze Bonifer[1,5,*]
[1]Institute for Cancer and Genomic Sciences, College of Medicine and Dentistry, University of Birmingham, B15 2TT Birmingham, UK
[2]Northern Institute for Cancer Research, University of Newcastle, Newcastle upon Tyne NE2 4HH, UK
[3]Department of Hematology, Erasmus University Medical Center, Dr. Molewaterplein 50, 3015 GE Rotterdam, the Netherlands
[4]These authors contributed equally
[5]Lead Contact
*Correspondence: c.bonifer@bham.ac.uk
http://dx.doi.org/10.1016/j.celrep.2017.05.005

SUMMARY

Acute myeloid leukemia (AML) is a heterogeneous disease caused by mutations in transcriptional regulator genes, but how different mutant regulators shape the chromatin landscape is unclear. Here, we compared the transcriptional networks of two types of AML with chromosomal translocations of the *RUNX1* locus that fuse the RUNX1 DNA-binding domain to different regulators, the t(8;21) expressing RUNX1-ETO and the t(3;21) expressing RUNX1-EVI1. Despite containing the same DNA-binding domain, the two fusion proteins display distinct binding patterns, show differences in gene expression and chromatin landscape, and are dependent on different transcription factors. RUNX1-EVI1 directs a stem cell-like transcriptional network reliant on GATA2, whereas that of RUNX1-ETO-expressing cells is more mature and depends on RUNX1. However, both types of AML are dependent on the continuous expression of the fusion proteins. Our data provide a molecular explanation for the differences in clinical prognosis for these types of AML.

INTRODUCTION

Acute myeloid leukemia (AML) is the most common acute leukemia in adults. Despite improvements in supportive care, outcome typically remains poor for AML patients older than 60 years who are unfit for intensive chemotherapy (Dennis et al., 2015). AML is highly heterogeneous and has been subdivided according to different categories of disease-causing mutations associated with different therapeutic responses. Subclasses are primarily defined by mutations in transcription factors, epigenetic regulators, and signaling molecules that affect cell growth and transcription factor activity (Cancer Genome Atlas Research Network, 2013; Papaemmanuil et al., 2016). Consequently, myeloid differentiation is impaired at different developmental stages, and different sets of genes are activated or repressed in distinct subsets of AML. Currently, the molecular details of how specific mutant transcriptional regulator proteins affect different sets of genes, and how such deregulated transcriptional networks impact myeloid differentiation, are unknown.

Mutations involving the hematopoietic master regulator RUNX1 are among the most commonly found abnormalities in AML. RUNX1 is the DNA-binding component of core binding factor (CBF), binding as a dimer with CBFβ, which is encoded by another recurrently rearranged gene in AML. The most common category of RUNX1 rearrangement is the product of the t(8;21) chromosomal translocation, RUNX1-ETO, which comprises the RUNX1 DNA-binding domain linked to the almost complete ETO protein (also known as RUNX1T1), which functions as a repressor by recruiting histone deacetylases (Bae et al., 1993; Erickson et al., 1992) (Figure 1A). The t(8;21) translocation involves 12% of newly diagnosed younger patients with AML (Grimwade et al., 2010). RUNX1-ETO leads to a block in myeloid differentiation (Cabezas-Wallscheid et al., 2013; Okuda et al., 1998; Regha et al., 2015), and its expression is required for leukemic propagation (Dunne et al., 2006; Heidenreich et al., 2003; Martinez et al., 2004; Ptasinska et al., 2012).

The product of another *RUNX1* translocation, t(3;21)(q26;q22), is RUNX1-EVI1, whereby the RUNT domain is fused to the entire *EVI1* gene (Figure 1A) (Mitani et al., 1994; Nucifora et al., 1994). *EVI1* (also known as *MECOM* or *PRDM3*) encodes a dual domain zinc-finger transcription factor with direct DNA-binding activity together with a histone methyl transferase (SET) domain (Morishita et al., 1995) (Figure 1A) and is an essential regulator of self-renewal in hematopoietic stem cells (Goyama et al., 2008). The t(3;21) translocation is rarely found in patients with de novo AML (Lugthart et al., 2010) and is more commonly found in those with therapy-related myelodysplastic syndrome (MDS)/AML (Rubin et al., 1990) or as a secondary event in the transformation of chronic myelogenous leukemia (CML) from chronic phase to blast crisis (Nukina et al., 2014).

Although RUNX1-ETO and RUNX1-EVI1 carry the same DNA-binding domain and bind to the same motifs in vitro (Meyers et al., 1993; Tanaka et al., 1995), the two classes of AML have distinct clinical characteristics. The t(8;21) translocation generally has a better clinical outcome than the t(3;21) translocation (Byrd et al., 2002; Grimwade et al., 2010; Slovak et al., 2000),

This is an open access article under the CC BY license (http://creativecommons.org/licenses/by/4.0/).

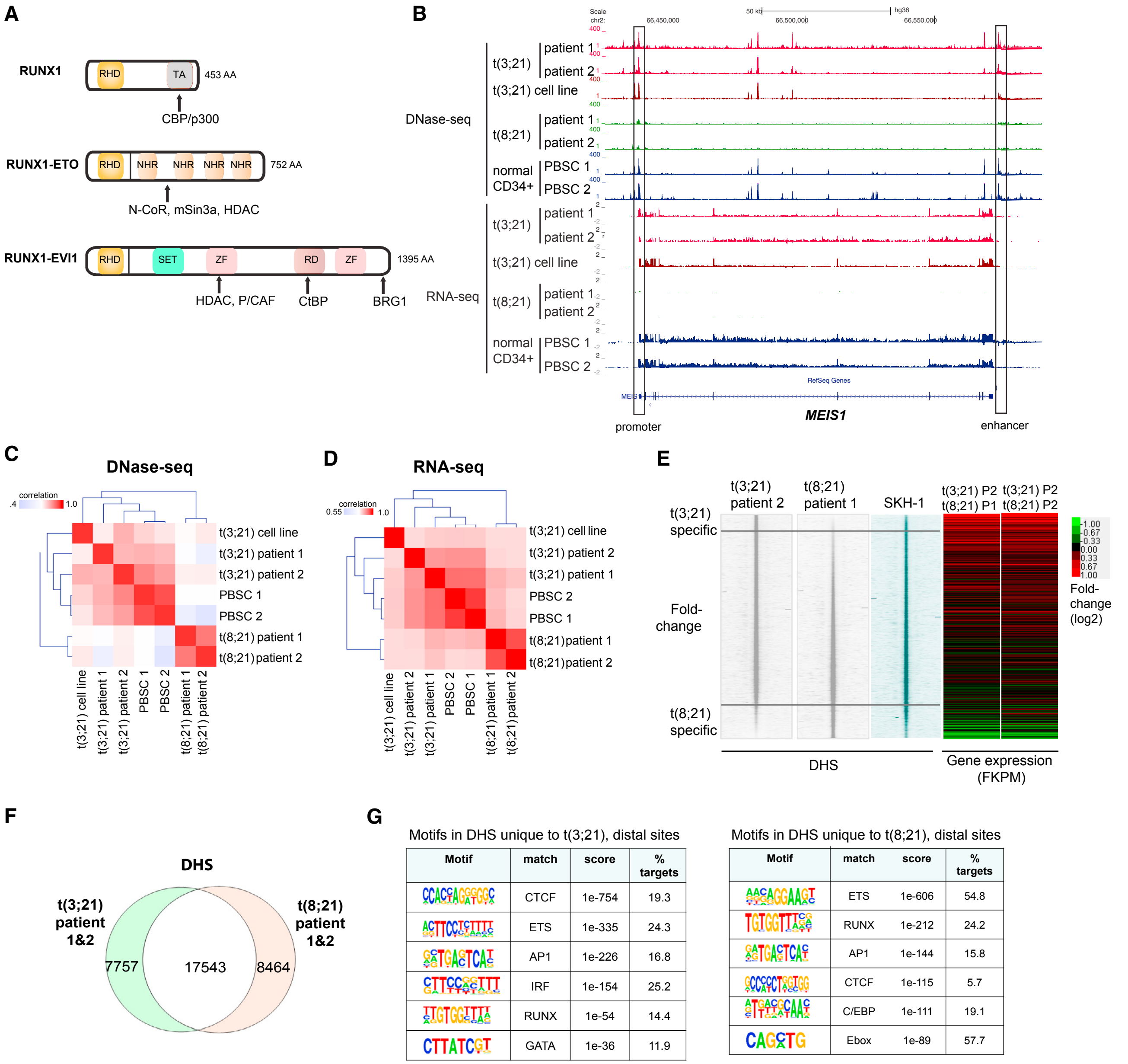

Figure 1. t(3;21) and t(8;21) Are Epigenetically Distinct Types of AML

(A) Structure of RUNX1, RUNX1-ETO, and RUNX1-EVI1 with their interacting partners. AA, amino acids; RHD, Runt homology domain; TA, transactivation domain; NHR, nervy homology region; SET, Su(var)3-9 and "Enhancer of zeste"; ZF, zinc finger domain; RD, proline-rich repressive domain; CBP, CREB-binding protein; HDAC, histone deacetylase; CtBP, C-terminal-binding protein; N-CoR, nuclear receptor co-repressor.

(B) UCSC genome browser screenshot of DNase-seq and corresponding RNA-seq in two patients with t(3;21) AML, two patients with t(8;21) AML, t(3;21) cell line, and normal CD34^{+} PBSCs at the *MEIS1* locus. An enhancer (Xiang et al., 2014) denoted at +140 kb is accessible in t(3;21) AML and normal CD34^{+} PBSCs, but not in t(8;21) AML.

(C) Clustering based on the strength of correlation between samples of DNase-seq data from cells of two patients with t(3;21), two patients with t(8;21), two independent CD34^{+} PBSCs, and the t(3;21) SKH-1 cell line.

(D) Correlation clustering of RNA-seq data (as in C) from two t(3;21) patients and the SKH-1 cell line with two t(8;21) patients and two normal CD34^{+} PBSCs.

(E) DNase-seq profiles spanning 4-kb windows for t(3;21) patient 2, t(8;21) patient 1, and SKH-1 cells. Peaks are ranked from top to bottom in order of increasing relative DNA sequence tag count for peaks identified in t(8;21) patient 1 relative to t(3;21) patient 2. The heatmaps to the right depict the relative expression of genes nearest to each DHS calculated as the ratio of FPKM values for t(3;21) patient 2 (P2) divided by values for t(8;21) patient 1 (P1) or patient 2 (P2).

(F) Venn diagram showing the overlap of DNase-seq peaks between t(3;21) patients (both patients combined) and t(8;21) patients (both patients combined).

(G) De novo motif discovery in distal DHSs unique to t(3;21) as compared to t(8;21) patients and distal DHSs unique to t(8;21) compared to t(3;21) patients (as shown in F).

See also Figure S1.

and the 5-year event-free survival for t(3;21) patients is only 14% (Lugthart et al., 2010). However, animal models with RUNX1-ETO and RUNX1-EVI1 expression do show similarities. Mice carrying *RUNX1-EVI1* knocked into the *RUNX1* locus display a phenotype similar to the *RUNX1-ETO* knockin (Maki et al., 2005; Okuda et al., 1998; Yergeau et al., 1997), as they die at embryonic day 13.5 (E13.5) with a failure of adult hematopoiesis. RUNX1-ETO and RUNX1-EVI1 also both require additional secondary mutations before they can cause AML in mice (Cuenco et al., 2000; Cuenco and Ren, 2001; Yuan et al., 2001), but RUNX1-EVI1 promotes a more aggressive leukemia with a reduced latency (Cuenco et al., 2000; Maki et al., 2006; Schessl et al., 2005; Schwieger et al., 2002). The molecular mechanisms underlying these similarities and differences in tumor pathology and clinical response are unclear. To address these issues, we compared the gene expression profiles as well as the chromatin landscape and transcription factor occupancy patterns of patients carrying the t(8;21) and t(3;21) translocations using global DNase I hypersensitive site (DHS) mapping, digital DNase I footprinting, and chromatin immunoprecipitation sequencing (ChIP-seq). These studies revealed that RUNX1-ETO and RUNX1-EVI1 associate with distinct subsets of regulatory elements that bind different classes of transcription factors and deregulate different sets of genes. As previously observed for RUNX1-ETO, depletion of RUNX1-EVI1 in t(3;21) cells initiates myeloid differentiation, which is linked to the upregulation of genes known to be vital for myeloid differentiation. Importantly, initiation of differentiation in either type of AML requires the presence of the master regulator of terminal myeloid differentiation, C/EBPα. Hence, despite having the same DNA-binding domain, our data show that the two different RUNX1 fusion proteins maintain the block in differentiation via unique gene regulatory networks.

RESULTS

t(3;21) and t(8;21) AML Display Different Epigenetic Landscapes and Gene Expression Profiles

In order to obtain a first indication of the similarities and differences in the cistromes regulating gene expression patterns in t(8;21) and t(3;21) AML we mapped the accessible chromatin landscape by identifying all DHSs in purified CD34$^+$ leukemic blast cells of two t(3;21) and two t(8;21) AML patients, two sets of normal CD34$^+$ progenitor cells purified as mobilized peripheral blood stem cells (PBSCs) from peripheral blood, and a t(3;21) cell line derived from a CML patient in blast crisis (SKH-1; Mitani et al., 1994)). We performed DNase I sequencing (DNase-seq) to identify all DHSs within chromatin as described previously (Ptasinska et al., 2012), and analyzed gene expression profiles using RNA sequencing (RNA-seq). These comparisons uncovered profound differences in gene expression profiles and DHS patterns between t(8;21) and t(3;21) AML, in particular with HOXA-associated genes such as *HOXA9* and its partner gene, *MEIS1*, which are highly expressed in t(3;21) malignancies, but not in t(8;21) AML (Figures 1B, S1A, and S1B). The SKH-1 cell line proved to be a surprisingly good model of primary t(3;21), as on average 90% of its DHSs overlapped with each of the two primary AMLs (Figures S1B, S1D, and S1E), despite the fact that all three cell types have a very different mutational background (Table S2). Correlation clustering analyses showed that DHS and gene expression profiles of t(3;21) and t(8;21) patients clustered separately as two distinct groups (Figures 1C and 1D) and showed differential gene expression and DHS patterns (Figures 1E, 1F, and S1D–S1F). Interestingly, for both DNase-seq and RNA-seq data, t(3;21) cells clustered closer to normal CD34$^+$ cells (PBSCs) than t(8;21) cells (Figures 1 C and 1D), suggesting a status close to early progenitor and stem cells for this type of AML. Furthermore, although RUNX, ETS, AP-1, and CTCF motifs were shared between the DNA motifs present within distal DHSs specific for the two patient classes, t(3;21) patients exhibited a specific enrichment for GATA motifs, whereas DHSs specific for t(8;21) were enriched in motifs for CEBP and E-box-binding factors, as observed previously (Figure 1G) (Ptasinska et al., 2014). In contrast, 90% of DHSs that are common to both t(3;21) and t(8;21) cells were also shared with normal CD34$^+$ PBSCs. Consistent with this finding, DHSs common to both types of AML regulate housekeeping functions (Figure S1C, right panel).

RUNX1-ETO and RUNX1-EVI1 Are Recruited to an Overlapping but Distinct Set of Binding Sites

The differential enrichment for GATA, CEBP, and E-box motifs prompted us to examine whether the binding patterns of RUNX1-ETO, RUNX1-EVI1, and RUNX1 differ between patient groups or whether the shared RHD DNA-binding domain would lead to similar binding pattern. This question is of significant interest, because RUNX1 and RUNX1-ETO have only one DNA-binding domain, whereas RUNX1-EVI1 has two additional potential DNA-binding domains derived from EVI1 (the zinc-finger domains; Figure 1A), which can contribute additional DNA specificity. It has been previously shown by in vitro studies that EVI1 binds to the GATA-like sequence GA(C/T)AAGA(T/C)AAGATAA (Delwel et al., 1993) and TGACAAGATAA (Perkins et al., 1991), which resemble one of the t(3;21)-specific motifs (Figure 1G). To investigate the in vivo specificity of the fusion proteins compared to RUNX1, we first generated RUNX1 and RUNX1-EVI1 ChIP-seq data from SKH-1 t(3;21) cells. We then compared these data with previously published RUNX1-ETO and RUNX1 ChIP data from the t(8;21) Kasumi-1 cell line (Ptasinska et al., 2012), as well as previously published RUNX1-binding data from primary CD34$^+$ cells (Cauchy et al., 2015). Both t(8;21) and t(3;21) AML co-express their respective fusion proteins together with wild-type RUNX1, but no expression of either EVI1 (Figure S2A) or ETO was detected, as reported previously (Mitani et al., 1994; Ptasinska et al., 2014). ETO and EVI1 antibodies therefore detected the fusion proteins, whereas the C-terminal RUNX1 antibody detected wild-type RUNX1 (Figure S2A, red).

A variety of tools were used in combination to analyze the ChIP datasets to demonstrate that despite similar total numbers of binding sites and genomic distribution, the two fusion proteins and RUNX1 each bind to overlapping but largely distinct sets of binding sites (Figures 2A–2D, S2C, S2G, and S3A). In Figures 2E and S2H, we ranked the RUNX1-ETO and the RUNX1-EVI1 or the respective RUNX1 ChIP peaks according to fold difference along the same genomic coordinates (Figures 2E, S2G, and S2H)

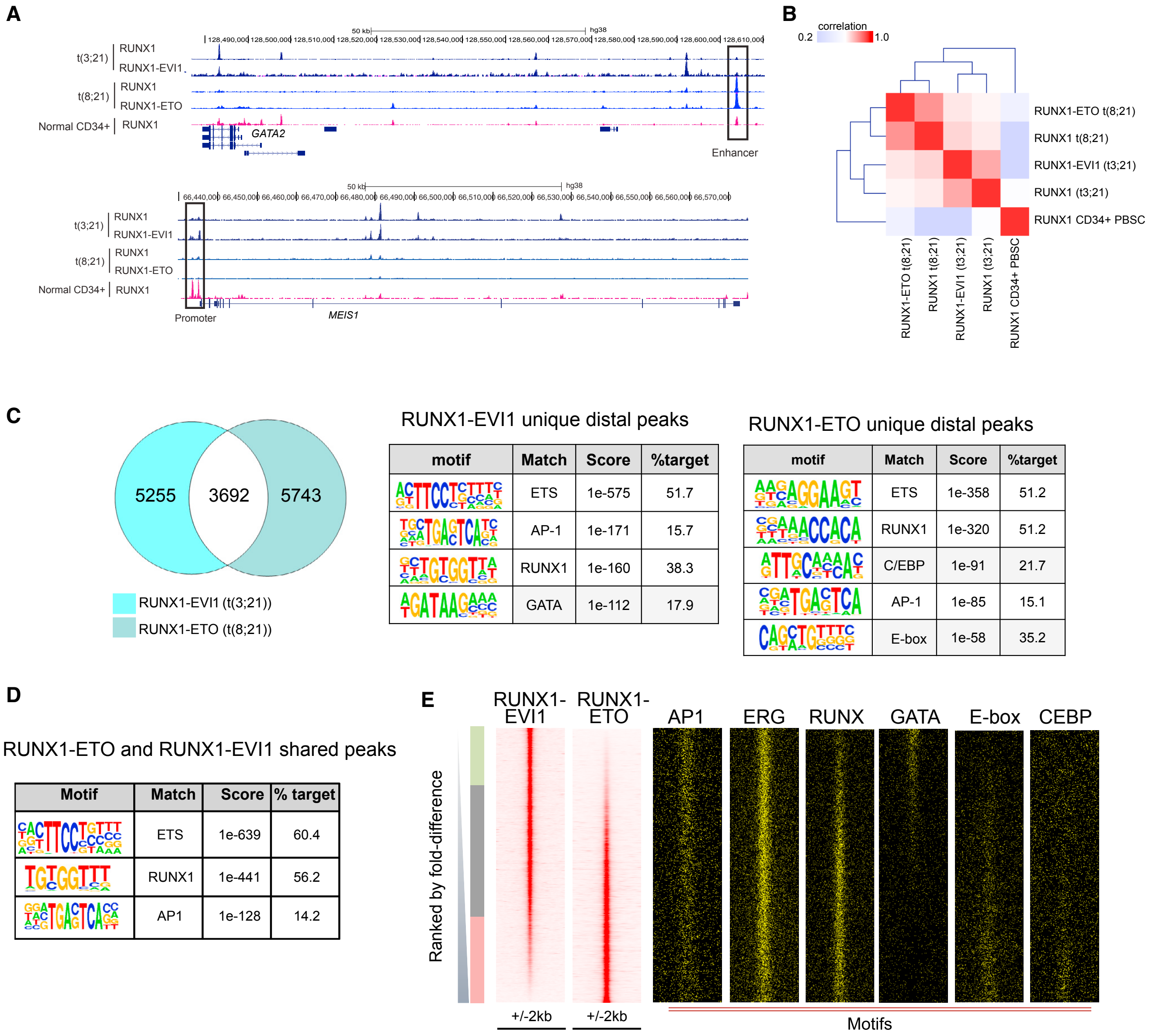

Figure 2. RUNX1-ETO and RUNX1-EVI1 Bind to Different Sites in Each Type of AML

(A) UCSC genome browser screen shot of aligned reads at *GATA2* and *MEIS1* from ChIP-seq experiments showing binding of RUNX1 (C-terminal antibody) and RUNX1-EVI1 from t(3;21) SKH-1 cells, RUNX1 and RUNX1-ETO from t(8;21) Kasumi-1 cells, and RUNX1 from normal CD34^{+} PBSCs. The boxed element indicates the *GATA2* enhancer.

(B) Matrix-based depiction of the correlation between ChIP-seq experiments followed by hierarchical clustering.

(C) Venn diagram of peak overlap between ChIP-seq for RUNX1-EVI1 in t(3;21) SKH-1 versus RUNX1-ETO in t(8;21) Kasumi-1. Tables depict de novo motif analyses of distal sites bound uniquely by each fusion protein. Gray highlights motifs found uniquely in either RUNX1-EVI1- or RUNX1-ETO-bound sites.

(D) Motif enrichment analysis in RUNX1-ETO and RUNX1-EVI1 shared peaks.

(E) RUNX1-EVI1 ChIP peaks were ranked according to tag count, and RUNX1-ETO peaks were plotted alongside, together with motifs for the indicated transcription factors.

See also Figure S2.

and then plotted the motifs, again along the same coordinates. These analyses show unequivocally that the two fusion proteins as well as RUNX1 show a distinct binding pattern in each cell type and that GATA motifs partition with RUNX1-EVI1, whereas E-box and C/EBP motifs partition with RUNX1-ETO. The same holds true for RUNX1 binding patterns (Figure S2H). Here, we also plotted our previously reported RUNX1-binding peaks from normal CD34^{+} cells alongside (Ptasinska et al., 2014), supporting the idea that the cistrome of t(3;21) cells is related to that of CD34^{+} cells. At important myeloid regulator genes, such as

the CSF-1 receptor gene (*CSF1R)* and the PU.1 gene (*SPI1*), the two fusion proteins target the same regulatory elements (Figures 3A and S3B) (Himes et al., 2001; Li et al., 2001). At many other sites the fusion protein binding sites co-localize specifically with alternate sets of other binding motifs (Figure 2C), such as GATA motifs in t(3;21) and C/EBP and E-Box sites in t(8;21). We did not detect the longer GATA-like motifs in the RUNX1-EVI1 ChIP-seq peaks identified in the in vitro studies (Figure S2E).

ChIP experiments in t(8;21) cells have shown that RUNX1-ETO co-associates with a number of hematopoietic regulators such as the E-box-binding protein HEB; the ETS factors ERG, FLI1, and PU.1; and the LMO2/LDB1 complex (Martens et al., 2012; Ptasinska et al., 2014; Sun et al., 2013). To test whether the GATA motifs in RUNX1-EVI1 peaks were bound by GATA factors, we examined the expression of GATA-family members in t(3;21) and t(8;21) patients and found that *GATA2*, but not *GATA1*, was expressed at a higher level in t(3;21) than in t(8;21) (Figure S3C). Other GATA factors were not expressed at all (Table S1). ChIP experiments demonstrated that RUNX1-EVI1, RUNX1, and GATA2 co-associated within a large population of sequences (Figures 3A, 3B, and S3D), which were characterized by ETS, RUNX, AP-1, and GATA motifs (Figures 3C and S3E). To identify other enriched motifs at the binding sites of RUNX1 and the two fusion proteins, we performed a more refined analysis examining the enrichment of multiple motifs at binding sites specific for each factor (see analysis scheme above Figure 3D) and cell type and then clustered the enrichment p-values (Figure 3D). Such an analysis highlights whether a set of motifs shows a higher enrichment in one cell type as compared to another, indicating the binding of different transcription factors around specific binding sites for each fusion protein and highlighting the relative importance of a transcription factor family in each cell type. For t(8;21) cells, this analysis showed that RUNX1 and RUNX1-ETO sites cluster separately with a strong enrichment of RUNX, C/EBP, and GFI1B motifs for both RUNX1-ETO and RUNX1 peaks (boxed in blue), a selective enrichment of E-box motifs for RUNX1-ETO peaks, and a specific enrichment for ETS family motifs for RUNX1 peaks. In contrast, RUNX1-EVI1 and RUNX1 peaks clustered together in t(3;21), with a strongly enriched motif signature for GATA, STAT, HOXA9, and ETS motifs (boxed in green).

To validate our ChIP data in primary cells, we performed digital DNase I footprinting and identified regions protected from nuclease digestion indicative of transcription factor binding using the Wellington algorithm (Piper et al., 2013). We then filtered footprints against our cell line ChIP data for RUNX1 (in t(8;21) and t(3;21)), RUNX1-EVI1, and RUNX1-ETO. Finally, we performed a bootstrapping analysis, which highlights the significance of occupied motif co-clustering within windows of 50 bp, and plotted enriched motifs in a co-clustering matrix (Figures 3E, 3F, S3F, and S3G). These analyses showed that RUNX1-EVI1-binding sites clustered with occupied PU.1/ERG (ETS), AP-1, and GATA motifs, suggesting that they may exist as a complex. This is in line with the fact that EVI1 has been shown to directly interact with the AP-1 family member FOS in several cell lines (Bard-Chapeau et al., 2012) and to co-localize with AP-1 motifs (Glass et al., 2013). In contrast, RUNX1-bound sites in t(3;21) only co-localized with occupied ERG (ETS) motifs in each cell type. However, the picture for RUNX1-ETO binding was different. Occupied RUNX1-ETO-bound sites clustered together with RUNX, ERG, and E-box motifs (Figure 3D), highlighting the nature of the RUNX1-ETO complex.

Survival of t(3;21) Cells Depends on the Continuous Expression of GATA2, but Not RUNX1

Our binding data suggested that RUNX1-EVI1 and RUNX1-ETO associate with different transcription factor complexes. Furthermore, such differential binding is also found with the wild-type RUNX1 protein expressed from the non-translocated allele in each AML type, indicating that RUNX1 fulfills different roles in programming the chromatin landscape in each cellular context. It was previously shown that the survival of t(8;21) cells is dependent on the expression of wild-type RUNX1, whereby RUNX1 regulated a complementary set of genes balancing the effects of RUNX1-ETO (Ben-Ami et al., 2013). We therefore tested whether this was also true for t(3;21) cells. To this end, we treated Kasumi-1 and SKH-1 cells with small interfering RNAs (siRNAs) specific for RUNX1 as well as control siRNAs and measured their survival using staining for the apoptosis marker Annexin V and propidium iodide (PI), which indicates dead cells. Figures 3G, 3H (left), and S3H–S3J demonstrate that after 5 days of knockdown, t(8;21) cells showed in increased cell death as compared to control cells, while SKH-1 cells showed no difference and thus do not require wild-type RUNX1.

Previous studies have shown that the members of the RUNX1-ETO complex (namely LMO2 and ERG) are required for the leukemogenicity of RUNX1-ETO and their survival (Sun et al., 2013). To gain first insights into whether GATA2 was preferentially required for the survival of t(3;21) cells, we depleted GATA2 in both t(3;21) and t(8;21) cells by siRNA treatment (Figures 3G and 3H, right, and Figures S3H–S3J) and measured their survival using staining for Annexin V and PI. These experiments show a strong increase in the number of apoptotic and dead cells in t(3;21) cells, but not in t(8;21) cells, indicating that GATA2 plays a more important role in the survival of t(3;21) cells than in t(8;21) cells.

Taken together, our data demonstrate that despite sharing the same DNA-binding domain, the two fusion proteins predominantly bind to different genomic sites, co-bind with different partners, and operate within a different chromatin landscape. Moreover, the transcriptional networks regulating the survival of both types of AML depend on different non-mutated transcription factors, with t(8;21) cell depending on RUNX1 and t(3;21) cell being dependent on GATA2.

RUNX1-EVI1 Is Required to Maintain the Undifferentiated Phenotype and Survival of t(3;21) AML

The t(8;21) translocation is a driver mutation (Wiemels et al., 2002), and RUNX1-ETO expression is required to maintain the leukemic phenotype in t(8;21) AML (Dunne et al., 2006). In contrast, RUNX1-EVI1 is a secondary mutation found in secondary AML and in CML in blast crisis (Nukina et al., 2014; Paquette et al., 2011; Rubin et al., 1987, 1990). We therefore used an siRNA knockdown approach to investigate whether RUNX1- was also required to maintain the full leukemic potential of t(3;21) cells.

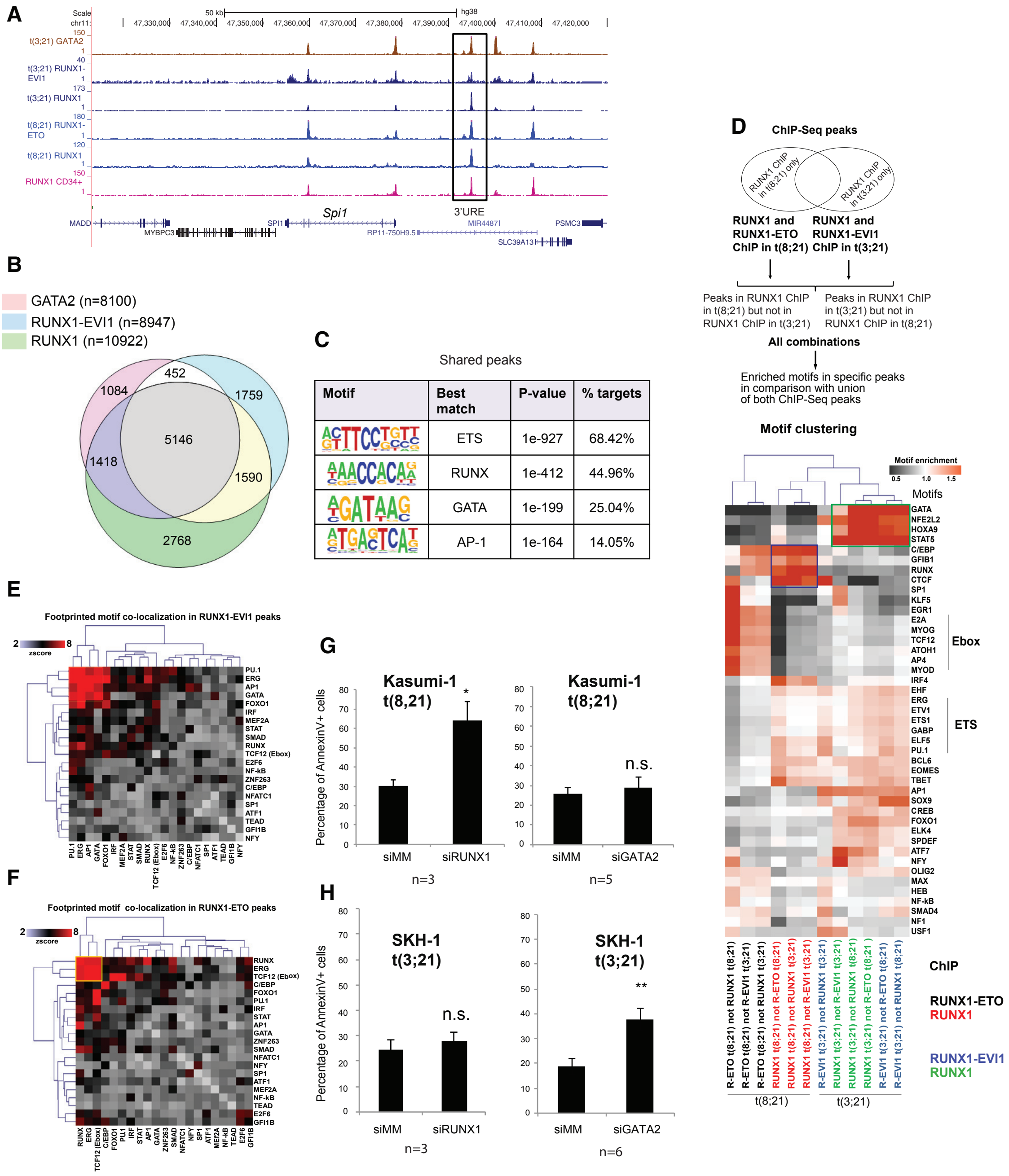

Figure 3. RUNX1 Fusion Proteins Form Part of a Gene Regulatory Network Unique to Each Leukemia

(A) UCSC genome browser screen shot of *Spi1* showing ChIP-seq data of RUNX1 and RUNX1-EVI1 from t(3;21) SKH-1 cells, RUNX1 and RUNX1-ETO from t(8;21) Kasumi-1 cells, and RUNX1 from normal CD34^{+} PBSCs.

(B) GATA2, RUNX1-EVI1, and RUNX1 ChIP-seq in t(3;21) SKH-1 cells. Venn diagram depicting the overlap between GATA2, RUNX1-EVI1, and RUNX1 peaks and the numbers of peaks in each group. White: overlap of GATA2- and RUNX1-EVI1-bound sites; purple: overlap of GATA2 and RUNX1 bound sites; yellow: overlap of RUNX1- and RUNX1-EVI1-bound sites; gray: number of sites bound by all three transcription factors.

(C) Transcription-factor-binding motifs enriched in the shared peaks from (B).

(D) Hierarchical clustering of enriched motifs discovered in a pairwise comparison between RUNX1 and RUNX1 fusion ChIP-seq peaks between t(3;21) and t(8;21) cells identifying unique peaks for each type of AML. Enrichment score was calculated by the level of motif enrichment in the unique peaks as compared to union of peaks in the pair of experiments. The heatmap depicts the degree of motif enrichment. Two specific sets of enriched motifs unique to each ChIP seq experiment are highlighted: the blue box highlights specifically enriched motifs in RUNX1-bound sites, the green box highlights enriched motifs specific for t(3;21) but not other peaks.

(E and F) Bootstrapping analysis of footprinted motifs at RUNX1-EVI1- or RUNX1-ETO-binding sites in patient cells. RUNX1-EVI1-binding sites from the t(3;21) SKH-1 cell line (E) and RUNX1-ETO-binding sites from the t(8;21) Kasumi-1 cell line (F) mapped onto footprints generated from DNase I data of either t(3;21)

(legend continued on next page)

We targeted the siRNA to the junction between RUNX1 and EVI1 to specifically deplete RUNX1-EVI1, but not RUNX1 (Figures S2A and S4A). We transfected SKH-1 with this siRNA and control siRNA, in parallel with control K562 cells, over a period of 2–14 days (Figure 4A). Flow cytometry revealed that SKH-1 cells transfected with RUNX1-EVI1 siRNA, but not control RNA, decreased the expression of the progenitor cell marker CD34 (Figures 4B and 4C). SKH-1 cells treated with siRNA, but not K562 cells, showed a diminished growth rate (Figure 4D) and started to undergo apoptosis (Figures S4B and S4C), indicating that the fusion protein is required for their survival. The analysis of RNA-seq data revealed significant changes in gene expression after knockdown (Figures 4E, 4F, and S4E; Table S1) with genes being progressively up- and downregulated. qPCR analyses confirmed that genes downregulated by siRNA included stem cell genes such as *GATA2* (Figures 5A–5C), whereas upregulated genes included myeloid differentiation markers such as *MPO*, *CSF1R*, *CTSG*, and *CEBPA* (Figures 5E–5H and S4F). The expression of *CEBPB* was unaffected (Figure 5D). Gene set enrichment analysis (GSEA) showed that the cells downregulated a stem cell program after knockdown of RUNX1-EVI1 (Figures S5A and S5B). Kyoto Encyclopedia of Genes and Genomes (KEGG) pathway analysis for RUNX1-EVI1 target genes downregulated after RUNX1-EVI1 knockdown highlighted multiple signaling genes, such as *PIM1*, *DUSP1*, *DUSP6*, *JAK1*, and *JAK3* (Figure 5I). A parallel analysis of upregulated genes identified *CEBPA*, *KIT*, and *MPO* (Figure S5F). A more refined picture was also seen when we analyzed downregulated core genes bound by RUNX1-EVI1, RUNX1, and GATA2 (Figure 5J). This analysis again identified genes encoding for factors important for stem cell function such as ERG, WT1, and MEIS1.

C/EBPα Is Required for the Response of t(3;21) Cells to RUNX1-EVI1 Knockdown

To identify factors that are involved in driving the differentiation of t(3;21) cells after RUNX1-EVI1 knockdown, we examined the changes in the epigenetic landscape of t(3;21) SKH-1 cells by mapping DHSs in cells treated with a control siRNA or after 10 days of knockdown with a RUNX1-EVI1-specific siRNA (Figure S5C). Examples of these data are depicted in the genome browser screenshots shown in Figures 6A, 6B, and S6A. We then ranked our DHS data according fold difference in sequence tag count (Figure S5D). This analysis revealed three groups of elements: a small group of peaks (group 1) unique for control cells, a large number of shared peaks (group 2), and 2,510 peaks that only appeared after knockdown (group 3). A de novo analysis of DNA motifs in these groups revealed that C/EBP motifs were specifically enriched in the DHSs gained after knockdown (Figure S5E). These results were concordant with the downregulation of *GATA2* and the upregulation of *CEBPA* expression after RUNX1-EVI1 knockdown (Figures 5A and 5H). To examine whether these changes in gene expression and motif composition were reflected in changes of binding of the respective factors, we measured the binding of RUNX1, GATA2, and C/EBPα before and after 10 days of RUNX1-EVI1 knockdown (Figures 6A, 6B, and S6A show screenshots). These experiments show that RUNX1-EVI1 knockdown did not influence the overall global genomic distribution of binding sites for these factors (Figure 6C) and did not influence the binding levels of RUNX1, although there was both a decrease in binding at some sites and increases in binding at others (Figures 6E and S6B). GATA2 binding decreased slightly overall and some binding sites were lost (Figures 6D and S6C), which can be explained by the lower expression of the *GATA2* gene (Figure 5A). These findings also demonstrated that GATA2 binding was not categorically dependent on the presence of RUNX1-EVI1. However, C/EBPα binding levels were increased (Figure 6D and 6E) with a number of new binding sites (Figure S6D). An alignment of DNA motifs and the DHS peaks confirmed that following RUNX1-EVI1 knockdown, C/EBP motif containing DHSs increased (group 3) in parallel with a depletion of GATA motif containing DHSs (group 1)(Figure 6F). In contrast, ERG, RUNX, and AP-1 motifs were relatively evenly distributed (Figure 6F). *CEBPA* upregulation is likely to be caused by the reduction of binding of RUNX1-EVI1 after knockdown with a concomitant increase of the binding of RUNX1 and C/EBPα itself to the *CEBPA* locus (Figure S6E).

We next examined whether upregulation of C/EBPα was required for the response to oncogene depletion. To this end, we transduced SKH-1 cells with a lentiviral vector expressing a dominant-negative CEBP peptide (DNCEBP) to block C/EBPα binding (and that of all other C/EBP factors) during knockdown RUNX1-EVI1 by siRNA. The DNCEBP peptide dimerizes with CEBP transcription factors and prevents binding to DNA (Krylov et al., 1995). Expression of the FLAG-epitope-tagged DNCEBP peptide was confirmed by western blotting (Figure 7A). We then treated control and DNCEBP SKH-1 cell lines with RUNX1-EVI1 siRNA (Figures S7A and S7B). While RUNX1-EVI1 knockdown decreased CD34 expression and proliferation, co-expression of the DNCEBP peptide rescued the leukemic phenotype (Figures 7B, 7C, and S7C). Similarly, DNCEBP-expressing cells maintained high expression of *HOXA9* after RUNX1-EVI1 knockdown, slowed the decrease of the key stem cell renewal genes *GATA2* and *MEIS1*, and blocked increased expression of the markers of myeloid differentiation *CTSG*, *MPO*, and *CSF1R* (Figures 7D, 7E, and S7B). ChIP experiments confirmed reduced C/EBPα binding at the corresponding loci after DNCEBP expression (Figure 7F). Another interesting finding was that the expression of DNCEBP also reduced the increase in RUNX1 binding at a set of known C/EBP and RUNX1 target genes (Figures 7F and 7G) with a concomitant

patient 2 or t(8;21) patient 1, respectively. The heatmap shows the significance of co-localizing footprinted motifs at RUNX1 fusion protein-binding sites for each AML as compared to sampling by chance alone.

(G and H) Percentage of Annexin-V-positive cells after 5 days of treatment with a control siRNA (siMM) or with siRNAs specific for RUNX1 and GATA2, respectively, in Kasumi-1 cells (G) and SKH1 cells (H). Each experiment was done at least in triplicate as indicated, and error bars represent SEM. *$p < 0.05$ and **$p < 0.01$ by unpaired t test. n.s. not significant.

See also Figure S3.

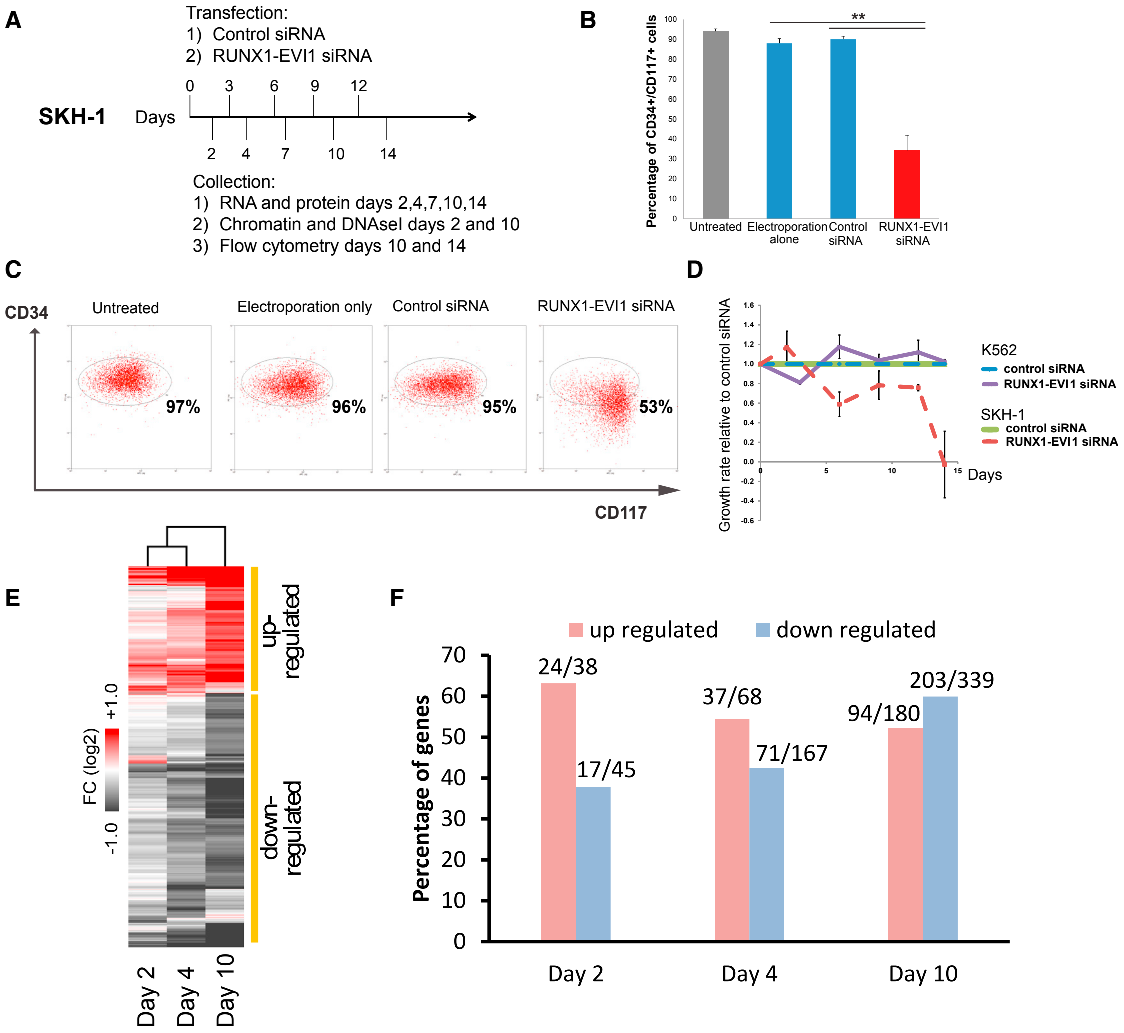

Figure 4. Knockdown of RUNX1-EVI1 Results in Loss of the Stem Cell Gene Program

(A) Experimental scheme for the siRNA transfection.

(B and C) RUNX1-EVI1 siRNA treatment in SKH-1 cells results in reduction in CD34 surface expression. SKH-1 cells after 14 days of either RUNX1-EVI1 or control siRNA transfection were stained with CD34-PE and CD117-APC. (B) Percentage of $CD34^{+}CD117^{+}$ cells. (C) Representative flow cytometry plot. Mean of six independent experiments is shown. Error bars represent SEM. *$p < 0.05$ by paired t test.

(D) Growth rates of SKH-1 (dashed lines) and K562 cells (solid lines) treated with RUNX1-EVI1 siRNA relative to treatment with control siRNA. The graph shows mean and SEM values from at least three independent experiments.

(E) Hierarchical clustering of gene expression changes as determined by RNA-seq at different time points of treatment. Unsupervised clustering of expression values of genes changing expression 1.5-fold after RUNX1-EVI1 siRNA transfection as compared to control siRNA. Average of two independent replicates. The heatmap color is related to the degree of differential expression (fold change [FC]) between RUNX1-EVI1 siRNA and control siRNA treatment.

(F) Percentage and number, respectively (on top of bars), of differentially expressed genes as measured by RNA-seq that are RUNX1-EVI1 ChIP-seq targets. Differentially expressed genes are those with an at least 1.5-fold change in gene expression between RUNX1-EVI1 siRNA as compared to control siRNA treatment.

See also Figure S4.

reduction in DNase I accessibility at genes strongly activated by RUNX1-EVI1 knockdown, such as *MPO* or *CTSG*, indicating that here, the cooperation of C/EBPα and RUNX1 is required for activation (Figures 7H and S7D).

Taken together, as summarized in Figure 7I, our study highlights how specific oncogenic transcription factors differentially program the epigenetic landscape in two types of AML with RUNX1 translocations but share the feature that they are dependent on the expression of the fusion protein and the suppression of C/EBPα to inhibit differentiation.

DISCUSSION

The study presented here used global analyses to investigate differences and similarities between two types of CBF AML: the t(8;21) expressing RUNX1-ETO and the t(3;21) expressing

OPEN
ACCESS
CellPress

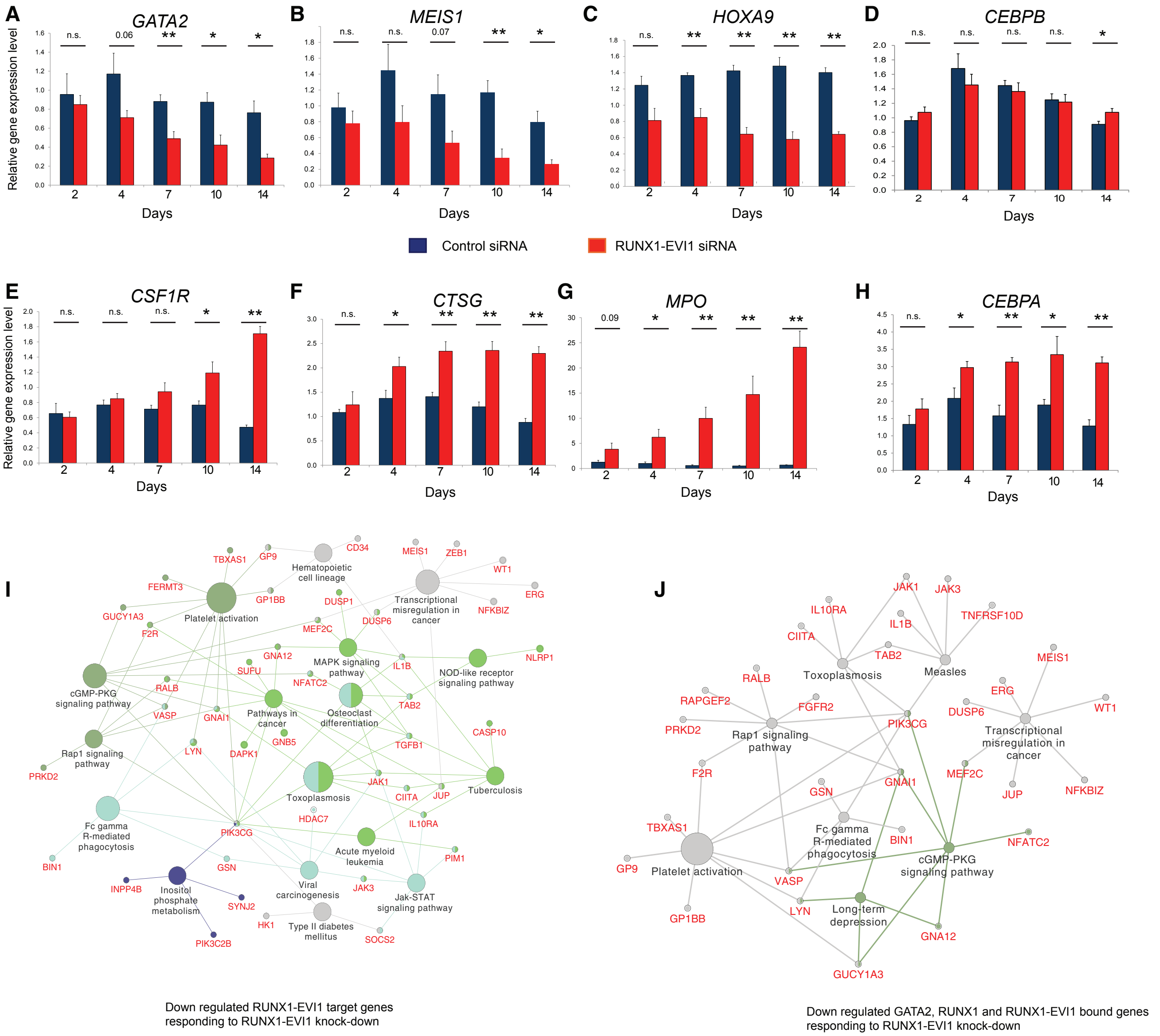

Figure 5. Knockdown of RUNX1-EVI1 Results in Loss of the Expression of Stem Cell Genes and the Upregulation of Myeloid Genes

(A–H) RT-PCR analysis of mRNA levels of the indicated genes relative to GAPDH and normalized to untreated cells in SKH-1 cells after RUNX1-EVI1 siRNA as compared to control siRNA transfection. *GATA2* (A), *MEIS1* (B), *HOXA9* (C), *CEBPB* (D), *CSF1R* (E), *CTSG* (F), MPO (G)*,* and *CEBPA* (H). The graph shows mean and SEM of four independent experiments. n.s., not significant; *p < 0.05; **p < 0.01 (unpaired t test). See also Table S1.

(I) KEGG pathways highlighting genes and pathways that are upregulated after RUNX1-EVI1 knockdown.

(J) KEGG pathways highlighting pathways associated with genes with shared binding of GATA2, RUNX1, and RUNX1-EVI1 that are upregulated after RUNX1-EVI1 knockdown.

See also Figure S5.

RUNX1-EVI1, which both carry the same RUNX1 DNA-binding domain. Our DHS mapping, digital footprinting experiments, and ChIP assays of patient cells and appropriate patient-derived model cell lines unequivocally determined how (1) the epigenetic and transcriptional profiles of the two types of AML differ and (2) show that the RUNT DNA-binding domain of each fusion protein is not the sole determining factor for the selection of fusion protein-binding sites in the genome. Moreover, each type of AML displays a unique, stable transcriptional network that is dependent on the presence of each fusion protein but requires a different set of associated transcription factors.

t(3;21) and t(8;21) AML Display Alternate Transcriptional Networks

The RUNX1-ETO and RUNX1-EVI1 fusion proteins are both unable to cause leukemia in mice on their own (Cuenco et al.,

OPEN ACCESS
CellPress

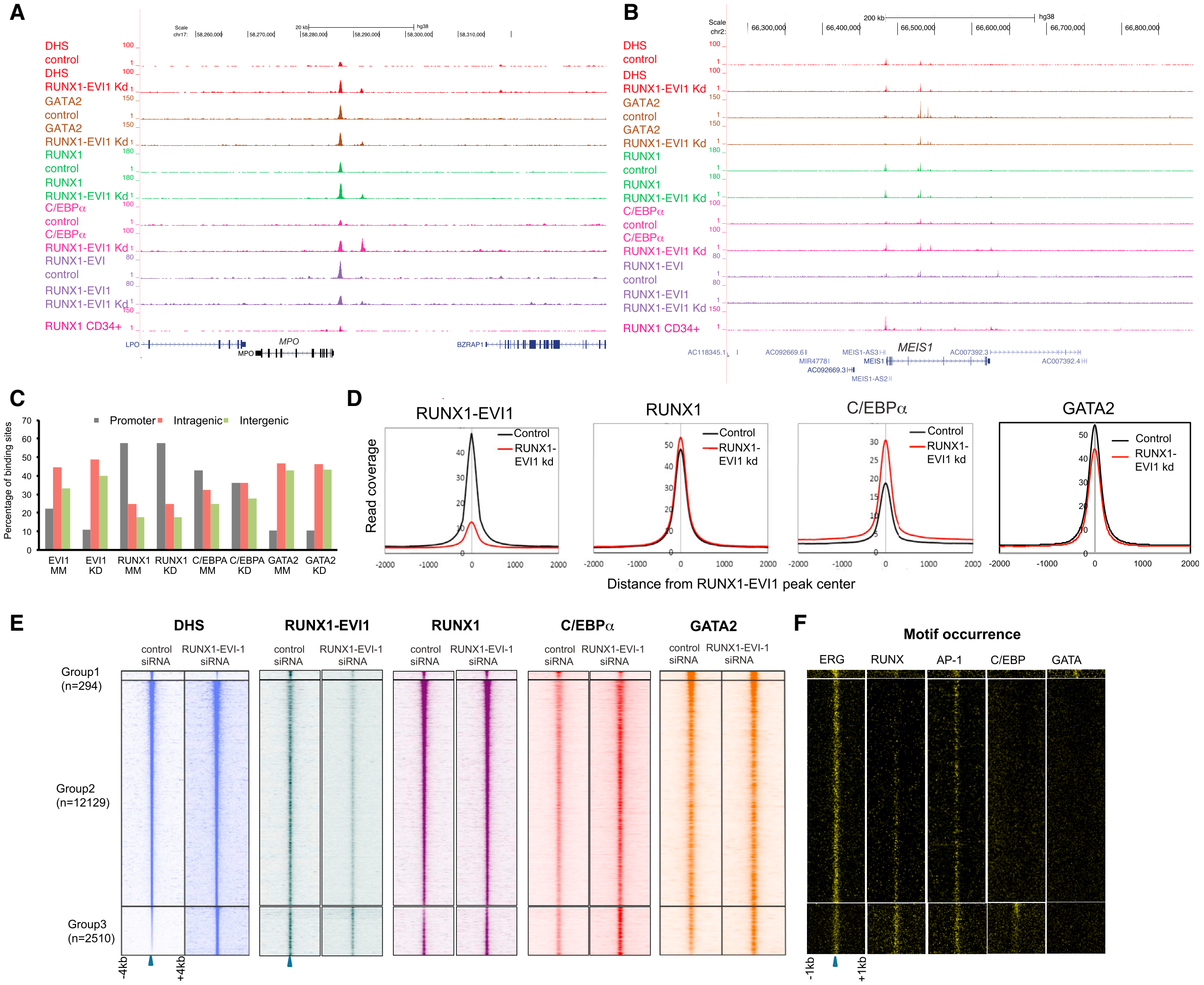

Figure 6. RUNX1-EVI1 Knockdown Results in Genome-wide Reprogramming of the Epigenome

(A and B) UCSC genome browser screen shots showing aligned reads at *MPO* (A) and *MEIS1* (B) depicting DNase-seq and RUNX1, RUNX1-EVI1, GATA2, and C/EBPα ChIP-seq data from SKH-1 after either control siRNA or RUNX1-EVI1 siRNA treatment.

(C) Genomic distribution of the indicated factors after transfection with either control or RUNX1-EVI1 siRNA.

(D) Average profiles of RUNX1-EVI1, RUNX1, C/EBPα, and GATA2 ChIP-seq reads centered on RUNX1-EVI1 peaks within a 4-kb window after transfection with either control or RUNX1-EVI1 siRNA.

(E) Profiles of the DNase-seq and ChIP-seq tag density for the indicated factors in ±4-kb windows centered on DHS for SKH-1 treated with either control or RUNX1-EVI1 siRNA. All peaks were ranked according to the fold change in DNase-seq tag counts between control siRNA and RUNX1-EVI1 siRNA-treated SKH-1 cells.

(F) Densities of the indicated motifs underlying the same coordinates plotted within ±1-kb windows around the DHS marked with a blue arrow.

See also Figure S6.

2000; Okuda et al., 1998), and they show a different history of tumor development in humans. The t(8;21) translocation is a primary mutation that hits an early stem cell (Miyamoto et al., 2000), whereas t(3;21) is often found in CML patients after blast crisis, indicating that during tumor initiation, the two fusion proteins encounter a dramatically different chromatin landscape that dictates where they can bind. Previous studies from our laboratory that used an inducible version of RUNX1-ETO expressed in murine myeloid precursor cells demonstrated that the induction of the fusion protein leads to a rapid downregulation of myeloid genes such as *Spi1*(PU.1) and *Cebpa* and a concomitant increase in the expression of stem cell genes such as *Gata2* and *Erg*, indicating extensive feed-forward loops driving myelopoiesis (Regha et al., 2015). It is likely that the same holds true for RUNX1-EVI1, but a different differentiation stage or previous transformation event may be required for the establishment of a stable transformed transcriptional network incorporating the expression of this powerful oncoprotein.

The unique transcriptional network maintained by RUNX1-EVI1 explains the difference in clinical outcomes of t(3;21) as compared to t(8;21) AML. RUNX1-EVI1 appears to directly regulate a stem cell program establishing an immature phenotype associated with treatment resistance, (Eppert et al., 2011), expressing genes (*MSI2* and *ZEB1*) regulating leukemia aggressiveness (Ito et al., 2010; Stavropoulou et al., 2016). Furthermore, *HOXA9* and *MEIS1* are both expressed in t(3;21), but not in t(8;21), AML. *HOXA9* expression is associated with poor prognosis (Andreeff et al., 2008; Golub et al., 1999), and is linked to a number of mutational subtypes, including mixed lineage leukemia (MLL) and NUP98 translocations (Collins and Hess, 2016). *MEIS1* expression is also associated with poor prognosis as part of a gene expression pattern seen in HSCs and LSCs (Eppert et al., 2011). *HOXA9* and *MEIS1* are often co-expressed in AML (Lawrence et al., 1999) and *Hoxa9* requires the co-expression of *Meis1* to transform murine bone marrow progenitor cells (Kroon et al., 1998). This cooperativity can be explained by the identification of a large number of cis-regulatory elements that are co-bound by both Hoxa9 and Meis1 (Huang et al., 2012).

The different gene regulatory networks maintaining the two types of AML involve alternate sets of transcription factors, and they differentially program the chromatin landscape, thus impacting where the fusion proteins bind. Our data show that RUNX1-ETO-binding sites are enriched for occupied ETS/RUNX/E-box motifs, reflecting the structure of the RUNX1-ETO complex, with the ETS factors ERG and FLI1 in the complex being required for leukemia maintenance and leukemogenesis (Martens et al., 2012; Sun et al., 2013). The expression of *ERG* in AML is generally associated with poor prognosis (Diffner et al., 2013). In contrast, RUNX1-EVI1 co-localizes with bound GATA2 and occupied AP-1 motifs, suggesting association with a different complex. Our data indeed show that high-level GATA2 expression is required for the survival of SKH-1 cells, but not t(8;21) cells, whereas RUNX1 regulates a complementary set of genes and is required for the survival of t(8;21) cells (Ben-Ami et al., 2013), but not SKH-1 cells (this study). High expression of GATA2 is indeed associated with poor prognosis in pediatric AML (Luesink et al., 2012), which may contribute to the fact that the t(3;21) is more aggressive than t(8;21). How each CBF fusion protein complex programs the DHS landscape, causing differential expression of members of the each complex, is exemplified by the regulation of *GATA2*. *GATA2* expression is higher in t(3;21) cells than in t(8;21) cells, which can be explained by a differential activity of its *cis*-regulatory elements. Our data show that a distal *GATA2* enhancer, known to upregulate *GATA2* expression (Gröschel et al., 2014), is accessible and bound by RUNX1 in normal $CD34^+$ cells, and by RUNX1 and RUNX1-ETO in t(8;21) patient cells, but neither RUNX1 nor RUNX1-EVI1 binds to this element in t(3;21) cells.

In summary, RUNX1 and both fusion protein complexes bind to AML-type specific *cis*-regulatory modules, which through auto-regulation of genes encoding complex members initiate the formation of stable gene regulatory networks that ultimately define the behavior of each type of AML (Pimanda and Göttgens, 2010).

C/EBPα Is Required for the Differentiation of t(8;21) and t(3;21) AML Cells after Oncoprotein Knockdown

Despite the differences between t(3;21) and t(8;21) transcriptional networks, C/EBPα is downregulated in both types of AML, suggesting that it is a critical node by which leukemia is maintained. We show that C/EBPα is directly repressed by both RUNX1-EVI1 and RUNX1-ETO through binding to a recently characterized upstream enhancer (Avellino et al., 2016). In both t(8;21) and t(3;21) cells, knockdown of the CBF fusion protein leads to upregulation of *CEBPA*, and our ChIP-seq data directly show that the binding of C/EBPα is affected by the knockdown of both fusion proteins. Conversely, in both AML types, the reduction of C/EBPα-binding activity by either knockdown (Ptasinska et al., 2014) or expression of a dominant-negative version of C/EBP (DNCEBP) blocks myelopoiesis and abolishes the upregulation of genes required for terminal myeloid function (*MPO*, *CSF1R*, and *CTSG*). This result complements previous data showing that overexpression of *CEBPA* can overcome the RUNX1-EVI1-mediated differentiation block (Tokita et al., 2007). An interesting finding from our study is that that the block of C/EBPα binding also abolishes the establishment of specific DHSs at certain genes and the binding of other transcription factors, including RUNX1. C/EBPα interacts with SWI/SNF nucleosome remodeling complexes, and this interaction is important for the development of adipocytes (Pedersen et al., 2001). This ability to initiate a global reprogramming of chromatin structures may be the main driver of C/EBPα-mediated myeloid differentiation, and current experiments focus on the mechanistic details of how this occurs.

Taken together, our study provides an important paradigm for studies aimed at understanding how different leukemic fusion proteins program and interact with the epigenetic landscape in two related but different types of AML. Our data represent a resource that will facilitate global mechanistic studies of the genes, transcription factors, and pathways involved in blocking myeloid differentiation and emphasize that different types of AML, despite being a disease of one specific differentiation pathway, are maintained by highly diverse transcriptional networks. Our study therefore highlights the complexities we have to face in our understanding of AML heterogeneity if we want to use this knowledge to devise AML-specific therapies.

EXPERIMENTAL PROCEDURES

Purification of Leukemic Cells and Mobilized Peripheral Stem Cells

Cells were purified as described previously (Cauchy et al., 2015), with minor modifications as outlined in in Supplemental Experimental Procedures.

siRNA-Mediated Depletion

1×10^7 cells were electroporated using an EPI 3500 (Fischer) at 350 V, 10 ms. siRNA sequences are listed in the Supplemental Experimental Procedures. siRNA was used at 200 nM. After electroporation, the cells remained in their cuvettes for 5 min before being directly added to RPMI-1640 with 10% fetal calf serum (FCS), supplemented with penicillin/streptomycin and glutamine at a concentration of 0.5×10^6 cells/mL, returned to an incubator, and kept at 37°C and 5% CO_2.

DHS Mapping, ChIP-Seq, and Digital Footprinting

DHS mapping, ChIP-seq, and digital footprinting using the Wellington algorithm (Piper et al., 2013) was performed as described previously (Cauchy

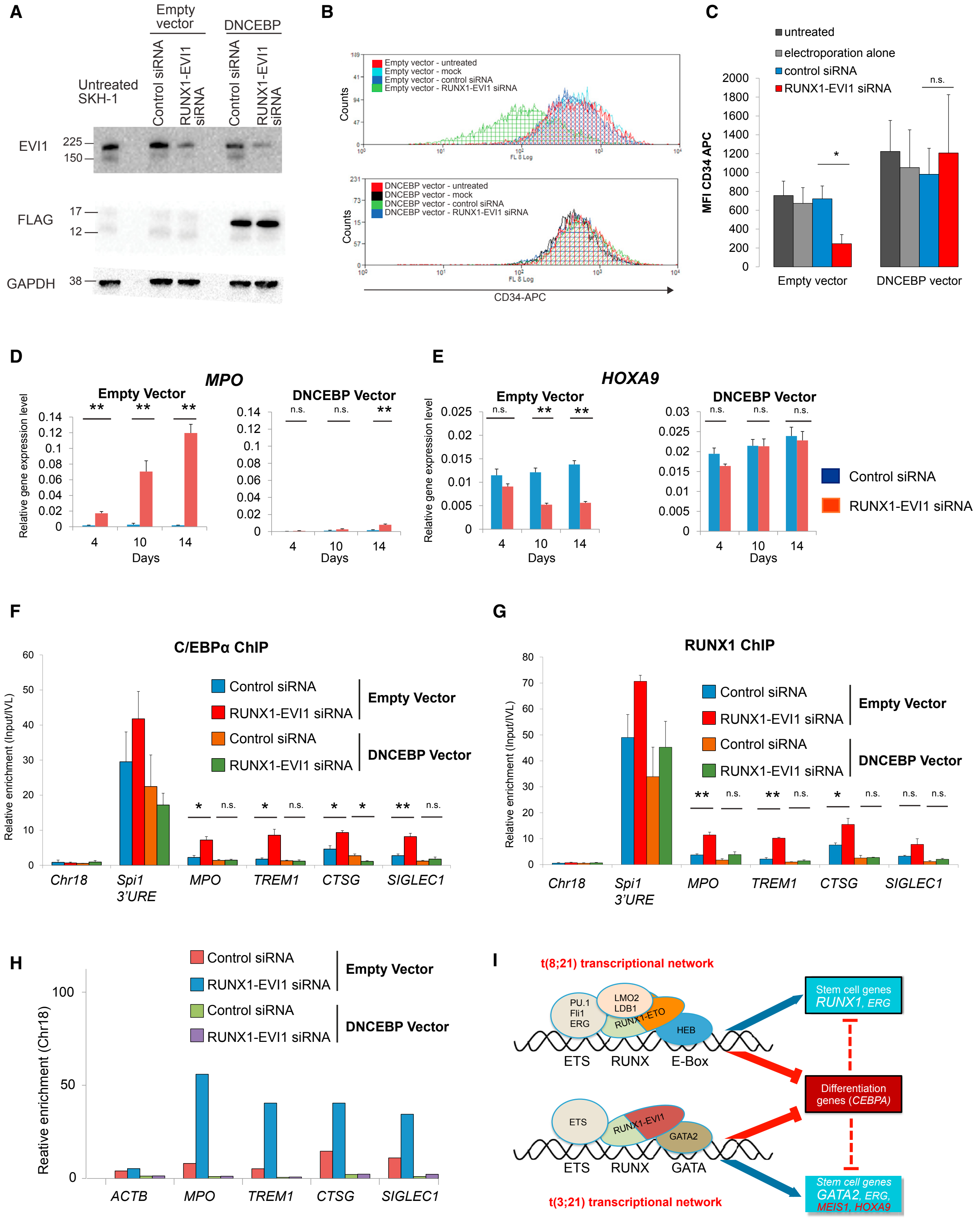

(legend on next page)

et al., 2015; Ptasinska et al., 2014), with minor modifications as outlined in in Supplemental Experimental Procedures. Details of antibodies and primers for qPCR are listed in Tables S3 and S4. Sequencing read data and list of peak numbers can be found in Table S3.

Data Analysis

Details of data analyses can be found in Supplemental Experimental Procedures.

Patient Samples

All human tissue was obtained with the required ethical approval from the National Health Service (NHS) National Research Ethics Committee. Detailed information about patient samples is listed in Table S2.

ACCESSION NUMBERS

The accession number for all t(3;21) next generation sequencing data reported in this paper is GEO: GSE87286.

SUPPLEMENTAL INFORMATION

Supplemental Information includes Supplemental Experimental Procedures, six figures, and four tables and can be found with this article online at http://dx.doi.org/10.1016/j.celrep.2017.05.005.

AUTHOR CONTRIBUTIONS

J.L. performed experiments and wrote the paper. M.R.I., A.P., Y.G., and N.M.S. performed experiments. S.A.A. and P.C. analyzed the data. M.R. and P.N.C. helped supervising the study and writing the paper. H.R.D. provided essential research material. O.H. designed and supervised experiments and helped writing the paper. C.B. designed the study, supervised experiments, and wrote the paper.

ACKNOWLEDGMENTS

This work was funded by a Clinical Training Fellowship from the Kay Kendall Leukemia Fund (KKL736) to J.L., M.R., and C.B., as well as by a Bloodwise programme grant (15001) to C.B. and P.N.C. OH was supported by grants from Bloodwise (12055), Cancer Research UK (C27943/A12788), and the North of England Children's Cancer Research Fund. We thank Stephen Kissane and Andrew Beggs and the staff of the Birmingham NGS sequence facility for expert services and support.

Received: February 16, 2017
Revised: April 13, 2017
Accepted: April 28, 2017
Published: May 23, 2017

REFERENCES

Andreeff, M., Ruvolo, V., Gadgil, S., Zeng, C., Coombes, K., Chen, W., Kornblau, S., Barón, A.E., and Drabkin, H.A. (2008). HOX expression patterns identify a common signature for favorable AML. Leukemia *22*, 2041–2047.

Avellino, R., Havermans, M., Erpelinck, C., Sanders, M.A., Hoogenboezem, R., van de Werken, H.J., Rombouts, E., van Lom, K., van Strien, P.M., Gebhard, C., et al. (2016). An autonomous CEBPA enhancer specific for myeloid-lineage priming and neutrophilic differentiation. Blood *127*, 2991–3003.

Bae, S.C., Yamaguchi-Iwai, Y., Ogawa, E., Maruyama, M., Inuzuka, M., Kagoshima, H., Shigesada, K., Satake, M., and Ito, Y. (1993). Isolation of PEBP2 alpha B cDNA representing the mouse homolog of human acute myeloid leukemia gene, AML1. Oncogene *8*, 809–814.

Bard-Chapeau, E.A., Jeyakani, J., Kok, C.H., Muller, J., Chua, B.Q., Gunaratne, J., Batagov, A., Jenjaroenpun, P., Kuznetsov, V.A., Wei, C.L., et al. (2012). Ecotopic viral integration site 1 (EVI1) regulates multiple cellular processes important for cancer and is a synergistic partner for FOS protein in invasive tumors. Proc. Natl. Acad. Sci. USA *109*, 2168–2173.

Ben-Ami, O., Friedman, D., Leshkowitz, D., Goldenberg, D., Orlovsky, K., Pencovich, N., Lotem, J., Tanay, A., and Groner, Y. (2013). Addiction of t(8;21) and inv(16) acute myeloid leukemia to native RUNX1. Cell Rep. *4*, 1131–1143.

Byrd, J.C., Mrózek, K., Dodge, R.K., Carroll, A.J., Edwards, C.G., Arthur, D.C., Pettenati, M.J., Patil, S.R., Rao, K.W., Watson, M.S., et al.; Cancer and Leukemia Group B (CALGB 8461) (2002). Pretreatment cytogenetic abnormalities are predictive of induction success, cumulative incidence of relapse, and overall survival in adult patients with de novo acute myeloid leukemia: results from Cancer and Leukemia Group B (CALGB 8461). Blood *100*, 4325–4336.

Cabezas-Wallscheid, N., Eichwald, V., de Graaf, J., Löwer, M., Lehr, H.A., Kreft, A., Eshkind, L., Hildebrandt, A., Abassi, Y., Heck, R., et al. (2013). Instruction of haematopoietic lineage choices, evolution of transcriptional landscapes and cancer stem cell hierarchies derived from an AML1-ETO mouse model. EMBO Mol. Med. *5*, 1804–1820.

Cancer Genome Atlas Research Network, Ley, T.J., Miller, C., Ding, L., Raphael, B.J., Mungall, A.J., Robertson, A., Hoadley, K., Triche, T.J., Jr., Laird, P.W., Baty, J.D., et al. (2013). Genomic and epigenomic landscapes of adult de novo acute myeloid leukemia. N. Engl. J. Med. *368*, 2059–2074.

Figure 7. C/EBPα DNA Binding Ability Is Critical for the Effects of RUNX1-EVI1 Knockdown

(A) Western blot analysis of whole-cell lysates of untreated t(3;21) SKH-1 cells and empty or DNCEBP vector transduced SKH-1 cells, transfected with either control or RUNX1-EVI1 siRNA after 14 days. Sizes (in kDa) are indicated. The blot was probed with EVI1, FLAG, or GAPDH antibodies (loading control) as indicated.

(B and C) Flow cytometry of empty and DNCEBP vector transduced SKH-1 cells, untreated or after 14 days of mock, control, or RUNX1-EVI1 siRNA treatment, stained with CD34-APC. (B) Representative histogram with overlay of different treatment conditions. (C) Graph of median fluorescence intensity (MFI) (median) of CD34-APC staining. Bars represent different treatment conditions. Mean of three independent experiments is shown, and error bars represent SEM. n.s., not significant; $^*p < 0.05$ (paired t test).

(D and E) RT-PCR analysis of mRNA levels of the indicated genes with and without RUNX1-EVI1 knockdown in the presence and absence of DNCEBP. (D) *MPO*. (E) *HOXA9*. mRNA levels relative to GAPDH in either empty vector or DNCEBP vector transduced SKH-1 after either control or RUNX1-EVI1 siRNA transfection (4, 10, or 14 days of treatment). The graph shows mean and SEM of three independent experiments. n.s., not significant; $^*p < 0.05$; $^{**}p < 0.01$ (unpaired t test).

(F and G) ChIP-qPCR with chromatin from empty or DNCEBP vector transduced SKH-1 10 days after either control or RUNX1-EVI1 siRNA transfection, using amplicons corresponding to the *MPO* and *SIGLEC1* enhancers, *TREM1* and *CTSG* promoters, the *SPI1* 3′ upstream regulatory element (URE) enhancer as a positive control, and chromosome 18 as a negative control. (F) C/EBPα ChIP. (G) RUNX1 ChIP. Enrichment was calculated relative to input and IVL. Mean of three independent experiments is shown, and error bars represent SEM. n.s., not significant; $^*p < 0.05$; $^{**}p < 0.01$ (unpaired t test).

(H) DNase I accessibility measurement using qPCR validation at *MPO* and *SIGLEC1* enhancer and *TREM1* and *CTSG* promoter. DNase I digestion was performed on empty vector and DNCEBP vector transduced SKH-1 following either control or RUNX1-EVI1 siRNA transfection. The *ACTB* amplicon was used as negative control. Enrichment was calculated relative to chromosome 18, which is a gene-free region that is DNase I inaccessible and used for normalization. A second independent experiment is shown in Figure S7D.

(I) Model depicting the binding sites and transcription factors interacting with RUNX1-ETO and RUNX1-EVI1, respectively, and their independent transcriptional networks that maintain the expression of stem cell/precursor genes but also block the expression of *CEBPA.* See also Figure S7.

Cauchy, P., James, S.R., Zacarias-Cabeza, J., Ptasinska, A., Imperato, M.R., Assi, S.A., Piper, J., Canestraro, M., Hoogenkamp, M., Raghavan, M., et al. (2015). Chronic FLT3-ITD signaling in acute myeloid leukemia is connected to a specific chromatin signature. Cell Rep. *12*, 821–836.

Collins, C.T., and Hess, J.L. (2016). Role of HOXA9 in leukemia: dysregulation, cofactors and essential targets. Oncogene *35*, 1090–1098.

Cuenco, G.M., and Ren, R. (2001). Cooperation of BCR-ABL and AML1/MDS1/EVI1 in blocking myeloid differentiation and rapid induction of an acute myelogenous leukemia. Oncogene *20*, 8236–8248.

Cuenco, G.M., Nucifora, G., and Ren, R. (2000). Human AML1/MDS1/EVI1 fusion protein induces an acute myelogenous leukemia (AML) in mice: a model for human AML. Proc. Natl. Acad. Sci. USA *97*, 1760–1765.

Delwel, R., Funabiki, T., Kreider, B.L., Morishita, K., and Ihle, J.N. (1993). Four of the seven zinc fingers of the Evi-1 myeloid-transforming gene are required for sequence-specific binding to GA(C/T)AAGA(T/C)AAGATAA. Mol. Cell. Biol. *13*, 4291–4300.

Dennis, M., Russell, N., Hills, R.K., Hemmaway, C., Panoskaltsis, N., McMullin, M.-F., Kjeldsen, L., Dignum, H., Thomas, I.F., Clark, R.E., et al. (2015). Vosaroxin and vosaroxin plus low-dose Ara-C (LDAC) vs low-dose Ara-C alone in older patients with acute myeloid leukemia. Blood *125*, 2923–2932.

Diffner, E., Beck, D., Gudgin, E., Thoms, J.A., Knezevic, K., Pridans, C., Foster, S., Goode, D., Lim, W.K., Boelen, L., et al. (2013). Activity of a heptad of transcription factors is associated with stem cell programs and clinical outcome in acute myeloid leukemia. Blood *121*, 2289–2300.

Dunne, J., Cullmann, C., Ritter, M., Soria, N.M., Drescher, B., Debernardi, S., Skoulakis, S., Hartmann, O., Krause, M., Krauter, J., et al. (2006). siRNA-mediated AML1/MTG8 depletion affects differentiation and proliferation-associated gene expression in t(8;21)-positive cell lines and primary AML blasts. Oncogene *25*, 6067–6078.

Eppert, K., Takenaka, K., Lechman, E.R., Waldron, L., Nilsson, B., van Galen, P., Metzeler, K.H., Poeppl, A., Ling, V., Beyene, J., et al. (2011). Stem cell gene expression programs influence clinical outcome in human leukemia. Nat. Med. *17*, 1086–1093.

Erickson, P., Gao, J., Chang, K.S., Look, T., Whisenant, E., Raimondi, S., Lasher, R., Trujillo, J., Rowley, J., and Drabkin, H. (1992). Identification of breakpoints in t(8;21) acute myelogenous leukemia and isolation of a fusion transcript, AML1/ETO, with similarity to Drosophila segmentation gene, runt. Blood *80*, 1825–1831.

Glass, C., Wuertzer, C., Cui, X., Bi, Y., Davuluri, R., Xiao, Y.Y., Wilson, M., Owens, K., Zhang, Y., and Perkins, A. (2013). Global identification of EVI1 target genes in acute myeloid leukemia. PLoS ONE *8*, e67134.

Golub, T.R., Slonim, D.K., Tamayo, P., Huard, C., Gaasenbeek, M., Mesirov, J.P., Coller, H., Loh, M.L., Downing, J.R., Caligiuri, M.A., et al. (1999). Molecular classification of cancer: class discovery and class prediction by gene expression monitoring. Science *286*, 531–537.

Goyama, S., Yamamoto, G., Shimabe, M., Sato, T., Ichikawa, M., Ogawa, S., Chiba, S., and Kurokawa, M. (2008). Evi-1 is a critical regulator for hematopoietic stem cells and transformed leukemic cells. Cell Stem Cell *3*, 207–220.

Grimwade, D., Hills, R.K., Moorman, A.V., Walker, H., Chatters, S., Goldstone, A.H., Wheatley, K., Harrison, C.J., and Burnett, A.K.; National Cancer Research Institute Adult Leukaemia Working Group (2010). Refinement of cytogenetic classification in acute myeloid leukemia: determination of prognostic significance of rare recurring chromosomal abnormalities among 5876 younger adult patients treated in the United Kingdom Medical Research Council trials. Blood *116*, 354–365.

Gröschel, S., Sanders, M.A., Hoogenboezem, R., de Wit, E., Bouwman, B.A., Erpelinck, C., van der Velden, V.H., Havermans, M., Avellino, R., van Lom, K., et al. (2014). A single oncogenic enhancer rearrangement causes concomitant EVI1 and GATA2 deregulation in leukemia. Cell *157*, 369–381.

Heidenreich, O., Krauter, J., Riehle, H., Hadwiger, P., John, M., Heil, G., Vornlocher, H.P., and Nordheim, A. (2003). AML1/MTG8 oncogene suppression by small interfering RNAs supports myeloid differentiation of t(8;21)-positive leukemic cells. Blood *101*, 3157–3163.

Himes, S.R., Tagoh, H., Goonetilleke, N., Sasmono, T., Oceandy, D., Clark, R., Bonifer, C., and Hume, D.A. (2001). A highly conserved c-fms gene intronic element controls macrophage-specific and regulated expression. J. Leukoc. Biol. *70*, 812–820.

Huang, Y., Sitwala, K., Bronstein, J., Sanders, D., Dandekar, M., Collins, C., Robertson, G., MacDonald, J., Cezard, T., Bilenky, M., et al. (2012). Identification and characterization of Hoxa9 binding sites in hematopoietic cells. Blood *119*, 388–398.

Ito, T., Kwon, H.Y., Zimdahl, B., Congdon, K.L., Blum, J., Lento, W.E., Zhao, C., Lagoo, A., Gerrard, G., Foroni, L., et al. (2010). Regulation of myeloid leukaemia by the cell-fate determinant Musashi. Nature *466*, 765–768.

Kroon, E., Krosl, J., Thorsteinsdottir, U., Baban, S., Buchberg, A.M., and Sauvageau, G. (1998). Hoxa9 transforms primary bone marrow cells through specific collaboration with Meis1a but not Pbx1b. EMBO J. *17*, 3714–3725.

Krylov, D., Olive, M., and Vinson, C. (1995). Extending dimerization interfaces: the bZIP basic region can form a coiled coil. EMBO J. *14*, 5329–5337.

Lawrence, H.J., Rozenfeld, S., Cruz, C., Matsukuma, K., Kwong, A., Kömüves, L., Buchberg, A.M., and Largman, C. (1999). Frequent co-expression of the HOXA9 and MEIS1 homeobox genes in human myeloid leukemias. Leukemia *13*, 1993–1999.

Li, Y., Okuno, Y., Zhang, P., Radomska, H.S., Chen, H., Iwasaki, H., Akashi, K., Klemsz, M.J., McKercher, S.R., Maki, R.A., and Tenen, D.G. (2001). Regulation of the PU.1 gene by distal elements. Blood *98*, 2958–2965.

Luesink, M., Hollink, I.H., van der Velden, V.H., Knops, R.H., Boezeman, J.B., de Haas, V., Trka, J., Baruchel, A., Reinhardt, D., van der Reijden, B.A., et al. (2012). High GATA2 expression is a poor prognostic marker in pediatric acute myeloid leukemia. Blood *120*, 2064–2075.

Lugthart, S., Gröschel, S., Beverloo, H.B., Kayser, S., Valk, P.J., van Zelderen-Bhola, S.L., Jan Ossenkoppele, G., Vellenga, E., van den Berg-de Ruiter, E., Schanz, U., et al. (2010). Clinical, molecular, and prognostic significance of WHO type inv(3)(q21q26.2)/t(3;3)(q21;q26.2) and various other 3q abnormalities in acute myeloid leukemia. J. Clin. Oncol. *28*, 3890–3898.

Maki, K., Yamagata, T., Asai, T., Yamazaki, I., Oda, H., Hirai, H., and Mitani, K. (2005). Dysplastic definitive hematopoiesis in AML1/EVI1 knock-in embryos. Blood *106*, 2147–2155.

Maki, K., Yamagata, T., Yamazaki, I., Oda, H., and Mitani, K. (2006). Development of megakaryoblastic leukaemia in Runx1-Evi1 knock-in chimaeric mouse. Leukemia *20*, 1458–1460.

Martens, J.H., Mandoli, A., Simmer, F., Wierenga, B.J., Saeed, S., Singh, A.A., Altucci, L., Vellenga, E., and Stunnenberg, H.G. (2012). ERG and FLI1 binding sites demarcate targets for aberrant epigenetic regulation by AML1-ETO in acute myeloid leukemia. Blood *120*, 4038–4048.

Martinez, N., Drescher, B., Riehle, H., Cullmann, C., Vornlocher, H.P., Ganser, A., Heil, G., Nordheim, A., Krauter, J., and Heidenreich, O. (2004). The oncogenic fusion protein RUNX1-CBFA2T1 supports proliferation and inhibits senescence in t(8;21)-positive leukaemic cells. BMC Cancer *4*, 44.

Meyers, S., Downing, J.R., and Hiebert, S.W. (1993). Identification of AML-1 and the (8;21) translocation protein (AML-1/ETO) as sequence-specific DNA-binding proteins: the runt homology domain is required for DNA binding and protein-protein interactions. Mol. Cell. Biol. *13*, 6336–6345.

Mitani, K., Ogawa, S., Tanaka, T., Miyoshi, H., Kurokawa, M., Mano, H., Yazaki, Y., Ohki, M., and Hirai, H. (1994). Generation of the AML1-EVI-1 fusion gene in the t(3;21)(q26;q22) causes blastic crisis in chronic myelocytic leukemia. EMBO J. *13*, 504–510.

Miyamoto, T., Weissman, I.L., and Akashi, K. (2000). AML1/ETO-expressing nonleukemic stem cells in acute myelogenous leukemia with 8;21 chromosomal translocation. Proc. Natl. Acad. Sci. USA *97*, 7521–7526.

Morishita, K., Suzukawa, K., Taki, T., Ihle, J.N., and Yokota, J. (1995). EVI-1 zinc finger protein works as a transcriptional activator via binding to a consensus sequence of GACAAGATAAGATAAN1-28 CTCATCTTC. Oncogene *10*, 1961–1967.

Nucifora, G., Begy, C.R., Kobayashi, H., Roulston, D., Claxton, D., Pedersen-Bjergaard, J., Parganas, E., Ihle, J.N., and Rowley, J.D. (1994). Consistent

intergenic splicing and production of multiple transcripts between AML1 at 21q22 and unrelated genes at 3q26 in (3;21)(q26;q22) translocations. Proc. Natl. Acad. Sci. USA *91*, 4004–4008.

Nukina, A., Kagoya, Y., Watanabe-Okochi, N., Arai, S., Ueda, K., Yoshimi, A., Nannya, Y., and Kurokawa, M. (2014). Single-cell gene expression analysis reveals clonal architecture of blast-phase chronic myeloid leukaemia. Br. J. Haematol. *165*, 414–416.

Okuda, T., Cai, Z., Yang, S., Lenny, N., Lyu, C.J., van Deursen, J.M.A., Harada, H., and Downing, J.R. (1998). Expression of a knocked-in AML1-ETO leukemia gene inhibits the establishment of normal definitive hematopoiesis and directly generates dysplastic hematopoietic progenitors. Blood *91*, 3134–3143.

Papaemmanuil, E., Gerstung, M., Bullinger, L., Gaidzik, V.I., Paschka, P., Roberts, N.D., Potter, N.E., Heuser, M., Thol, F., Bolli, N., et al. (2016). Genomic classification and prognosis in acute myeloid leukemia. N. Engl. J. Med. *374*, 2209–2221.

Paquette, R.L., Nicoll, J., Chalukya, M., Elashoff, D., Shah, N.P., Sawyers, C., Spiteri, E., Nanjangud, G., and Rao, P.N. (2011). Frequent EVI1 translocations in myeloid blast crisis CML that evolves through tyrosine kinase inhibitors. Cancer Genet. *204*, 392–397.

Pedersen, T.A., Kowenz-Leutz, E., Leutz, A., and Nerlov, C. (2001). Cooperation between C/EBPalpha TBP/TFIIB and SWI/SNF recruiting domains is required for adipocyte differentiation. Genes Dev. *15*, 3208–3216.

Perkins, A.S., Fishel, R., Jenkins, N.A., and Copeland, N.G. (1991). Evi-1, a murine zinc finger proto-oncogene, encodes a sequence-specific DNA-binding protein. Mol. Cell. Biol. *11*, 2665–2674.

Pimanda, J.E., and Göttgens, B. (2010). Gene regulatory networks governing haematopoietic stem cell development and identity. Int. J. Dev. Biol. *54*, 1201–1211.

Piper, J., Elze, M.C., Cauchy, P., Cockerill, P.N., Bonifer, C., and Ott, S. (2013). Wellington: a novel method for the accurate identification of digital genomic footprints from DNase-seq data. Nucleic Acids Res. *41*, e201.

Ptasinska, A., Assi, S.A., Mannari, D., James, S.R., Williamson, D., Dunne, J., Hoogenkamp, M., Wu, M., Care, M., McNeill, H., et al. (2012). Depletion of RUNX1/ETO in t(8;21) AML cells leads to genome-wide changes in chromatin structure and transcription factor binding. Leukemia *26*, 1829–1841.

Ptasinska, A., Assi, S.A., Martinez-Soria, N., Imperato, M.R., Piper, J., Cauchy, P., Pickin, A., James, S.R., Hoogenkamp, M., Williamson, D., et al. (2014). Identification of a dynamic core transcriptional network in t(8;21) AML that regulates differentiation block and self-renewal. Cell Rep. *8*, 1974–1988.

Regha, K., Assi, S.A., Tsoulaki, O., Gilmour, J., Lacaud, G., and Bonifer, C. (2015). Developmental-stage-dependent transcriptional response to leukaemic oncogene expression. Nat. Commun. *6*, 7203.

Rubin, C.M., Larson, R.A., Bitter, M.A., Carrino, J.J., Le Beau, M.M., Diaz, M.O., and Rowley, J.D. (1987). Association of a chromosomal 3;21 translocation with the blast phase of chronic myelogenous leukemia. Blood *70*, 1338–1342.

Rubin, C.M., Larson, R.A., Anastasi, J., Winter, J.N., Thangavelu, M., Vardiman, J.W., Rowley, J.D., and Le Beau, M.M. (1990). t(3;21)(q26;q22): a recurring chromosomal abnormality in therapy-related myelodysplastic syndrome and acute myeloid leukemia. Blood *76*, 2594–2598.

Schessl, C., Rawat, V.P.S., Cusan, M., Deshpande, A., Kohl, T.M., Rosten, P.M., Spiekermann, K., Humphries, R.K., Schnittger, S., Kern, W., et al. (2005). The AML1-ETO fusion gene and the FLT3 length mutation collaborate in inducing acute leukemia in mice. J. Clin. Invest. *115*, 2159–2168.

Schwieger, M., Löhler, J., Friel, J., Scheller, M., Horak, I., and Stocking, C. (2002). AML1-ETO inhibits maturation of multiple lymphohematopoietic lineages and induces myeloblast transformation in synergy with ICSBP deficiency. J. Exp. Med. *196*, 1227–1240.

Slovak, M.L., Kopecky, K.J., Cassileth, P.A., Harrington, D.H., Theil, K.S., Mohamed, A., Paietta, E., Willman, C.L., Head, D.R., Rowe, J.M., et al. (2000). Karyotypic analysis predicts outcome of preremission and postremission therapy in adult acute myeloid leukemia: a Southwest Oncology Group/Eastern Cooperative Oncology Group Study. Blood *96*, 4075–4083.

Stavropoulou, V., Kaspar, S., Brault, L., Sanders, M.A., Juge, S., Morettini, S., Tzankov, A., Iacovino, M., Lau, I.J., Milne, T.A., et al. (2016). MLL-AF9 expression in hematopoietic stem cells drives a highly invasive AML expressing EMT-related genes linked to poor outcome. Cancer Cell *30*, 43–58.

Sun, X.J., Wang, Z., Wang, L., Jiang, Y., Kost, N., Soong, T.D., Chen, W.Y., Tang, Z., Nakadai, T., Elemento, O., et al. (2013). A stable transcription factor complex nucleated by oligomeric AML1-ETO controls leukaemogenesis. Nature *500*, 93–97.

Tanaka, T., Mitani, K., Kurokawa, M., Ogawa, S., Tanaka, K., Nishida, J., Yazaki, Y., Shibata, Y., and Hirai, H. (1995). Dual functions of the AML1/Evi-1 chimeric protein in the mechanism of leukemogenesis in t(3;21) leukemias. Mol. Cell. Biol. *15*, 2383–2392.

Tokita, K., Maki, K., and Mitani, K. (2007). RUNX1/EVI1, which blocks myeloid differentiation, inhibits CCAAT-enhancer binding protein alpha function. Cancer Sci. *98*, 1752–1757.

Wiemels, J.L., Xiao, Z., Buffler, P.A., Maia, A.T., Ma, X., Dicks, B.M., Smith, M.T., Zhang, L., Feusner, J., Wiencke, J., et al. (2002). In utero origin of t(8;21) AML1-ETO translocations in childhood acute myeloid leukemia. Blood *99*, 3801–3805.

Xiang, P., Wei, W., Lo, C., Rosten, P., Hou, J., Hoodless, P.A., Bilenky, M., Bonifer, C., Cockerill, P.N., Kirkpatrick, A., et al. (2014). Delineating MEIS1 cis-regulatory elements active in hematopoietic cells. Leukemia *28*, 433–436.

Yergeau, D.A., Hetherington, C.J., Wang, Q., Zhang, P., Sharpe, A.H., Binder, M., Marín-Padilla, M., Tenen, D.G., Speck, N.A., and Zhang, D.E. (1997). Embryonic lethality and impairment of haematopoiesis in mice heterozygous for an AML1-ETO fusion gene. Nat. Genet. *15*, 303–306.

Yuan, Y., Zhou, L., Miyamoto, T., Iwasaki, H., Harakawa, N., Hetherington, C.J., Burel, S.A., Lagasse, E., Weissman, I.L., Akashi, K., and Zhang, D.E. (2001). AML1-ETO expression is directly involved in the development of acute myeloid leukemia in the presence of additional mutations. Proc. Natl. Acad. Sci. USA *98*, 10398–10403.

Cell Reports, Volume *19*

Supplemental Information

RUNX1-ETO and RUNX1-EVI1 Differentially Reprogram the Chromatin Landscape in t(8;21) and t(3;21) AML

Justin Loke, Salam A. Assi, Maria Rosaria Imperato, Anetta Ptasinska, Pierre Cauchy, Yura Grabovska, Natalia Martinez Soria, Manoj Raghavan, H. Ruud Delwel, Peter N. Cockerill, Olaf Heidenreich, and Constanze Bonifer

Supplemental information

Contents

Supplemental figures and legends 3
Supplemental Figure 1 (related to Figure 1) 3
Supplemental Figure 2 (related to Figure 2) 5
Supplemental Figure 3 (related to Figure 3) 7
Supplemental Figure 4 (related to Figure 4) 9
Supplemental Figure 5 (related to Figure 5) 11
Supplemental Figure 6 (related to Figure 6) 13
Supplemental Figure 7 (related to Figure 7) 15
Supplemental Experimental Procedures 17
Purification of blood samples from patients with AML 17
Purification of CD34+ mobilized peripheral blood stem cells 17
siRNA mediated depletion of RUNX1-EVI1, RUNX1 and GATA2 17
RNA extraction 18
RNA Seq libraries 18
cDNA synthesis 19
Real-time polymerase chain reaction 19
Dead cell removal and Annexin V/PI staining for flow cytometry 19
DNaseI hypersensitivity site mapping 19
Library production of DNase I material for high throughput sequencing 20
ChIP-qPCR and ChIP-Seq library preparation 20
Double cross-linking 20
Chromatin immunoprecipitation (ChIP) 20
Library production of ChIP material for high throughput sequencing 21
Retroviral production 21
Transfection of HEK293T cells for lentiviral production 21
Virus concentration 22
Lentiviral transduction of SKH-1 22
Antibody staining for flow cytometry 22
Whole cell lysate preparation by RIPA buffer lysis 22
Nuclear extract 22
Western blotting 23
Oligonucleotide sequences and antibodies 23
ChIP and DNase I sequencing data Analysis 25
Alignment 25

Peak calling 25
Clustering of ChIP and DNAseI sequencing data 25
Average tag density profile and heatmap 26
Motif identification and clustering 26
Motif clustering 26
Motif enrichment 26
RNA-Seq data Analysis 27
Supplemental tables 30

Supplemental figures and legends

Figure S1

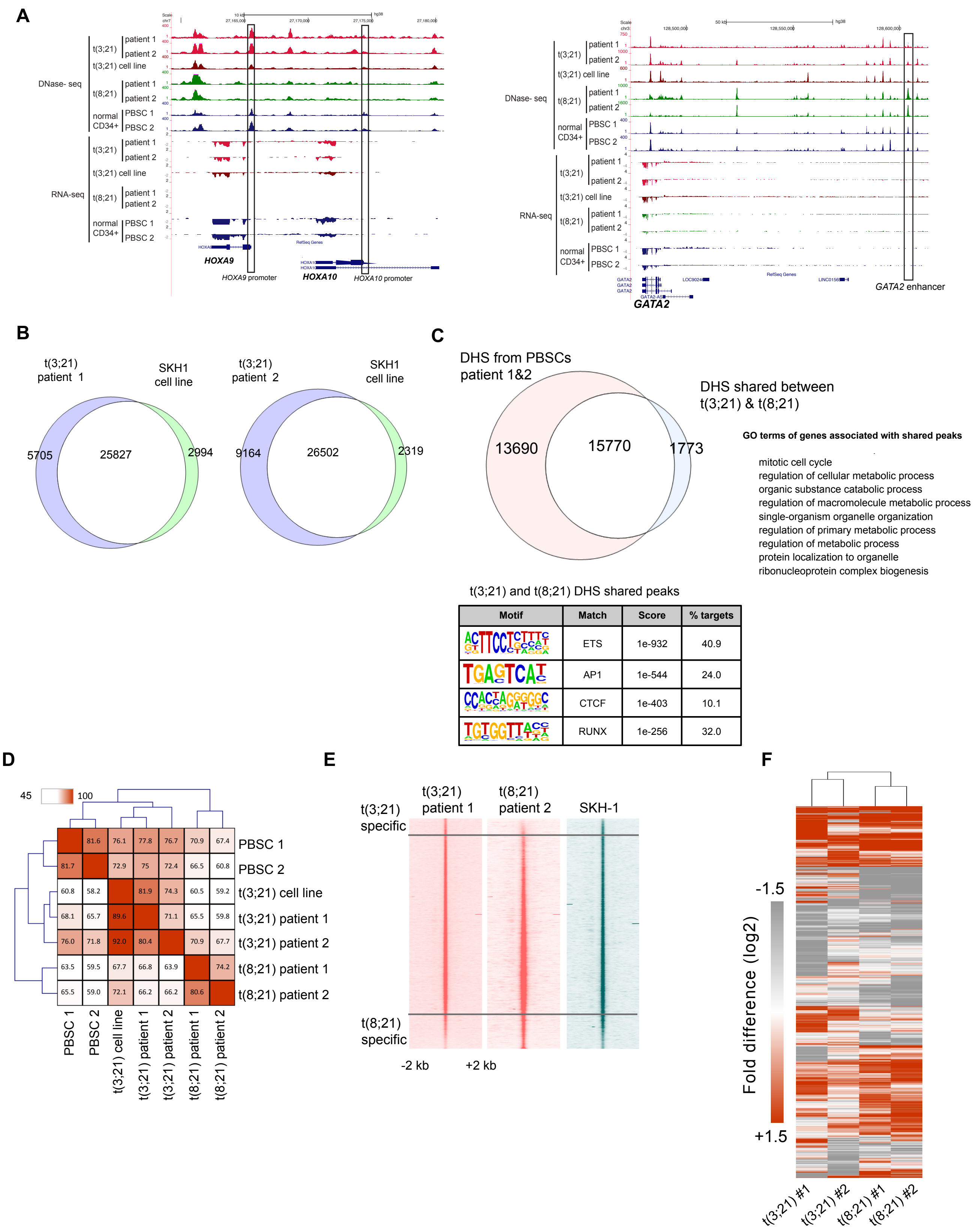

Supplemental Figure 1 (related to Figure 1)

A-B) UCSC genome browser screenshots of DNase-Seq and the corresponding RNA-Seq aligned reads from two patients with t(3;21) AML, two patients with t(8;21) AML, the t(3;21) cell line SKH-1, and normal CD34+ PBSCs. The regions depicted here span the *HOXA9/HOXA10* loci and the *GATA2* locus. The HOXA9 and HOXA10 loci include DHSs present in t(3;21) cells but not in t(8;21), whereas the *GATA2* encompass DHSs present in t(8;21) cells but not t(3;21) cells.

B) Venn diagram showing DHS overlap between t(3;21) patient 1 and SKH-1 cells, and also between t(3;21) patient 2 and SKH-1. Labels represent numbers of DHSs.

C) Venn diagram overlapping the DHS shared between t(3;21) and t(8;21) patient cells with those of CD34+ cells . Right pane: GO terms of genes associated with shared peaks. Bottom panel: enriched binding motifs in shared peaks.

D) Percentage overlap of DHS peaks in a pairwise comparison between each sample for the t(3;21) and t(8;21) patients and two samples of normal CD34+PBSCs. The clustering analysis defined three major clusters corresponding to (i) CD34+ PBSCs, (ii) t(3;21) patient samples and cell line, and (iii) t(8;21) patient samples.

E) DNase-Seq profiles spanning 4 kb windows for t(3;21) patient 1, t(8;21) patient 2, and SKH-1 cells. Peaks are ranked from top to bottom in order of increasing relative DNA sequence tag count for peaks identified in t(8;21) patient 2 relative to t(3;21) patient 1. The horizontal lines indicate the thresholds for peaks defined as either t(3;21) (top) or as t(8;21)-specific (bottom).

F) Hierarchical clustering of each RNA-Seq experiment by gene expression (fold difference in comparison with normal CD34+ PBSCs (expressed as log2 values). Clustering is based on the differential expression of genes in each patient sample, and reveals that patients with t(3;21) leukemia cluster apart from patients with t(8;21) leukemia.

Figure S2

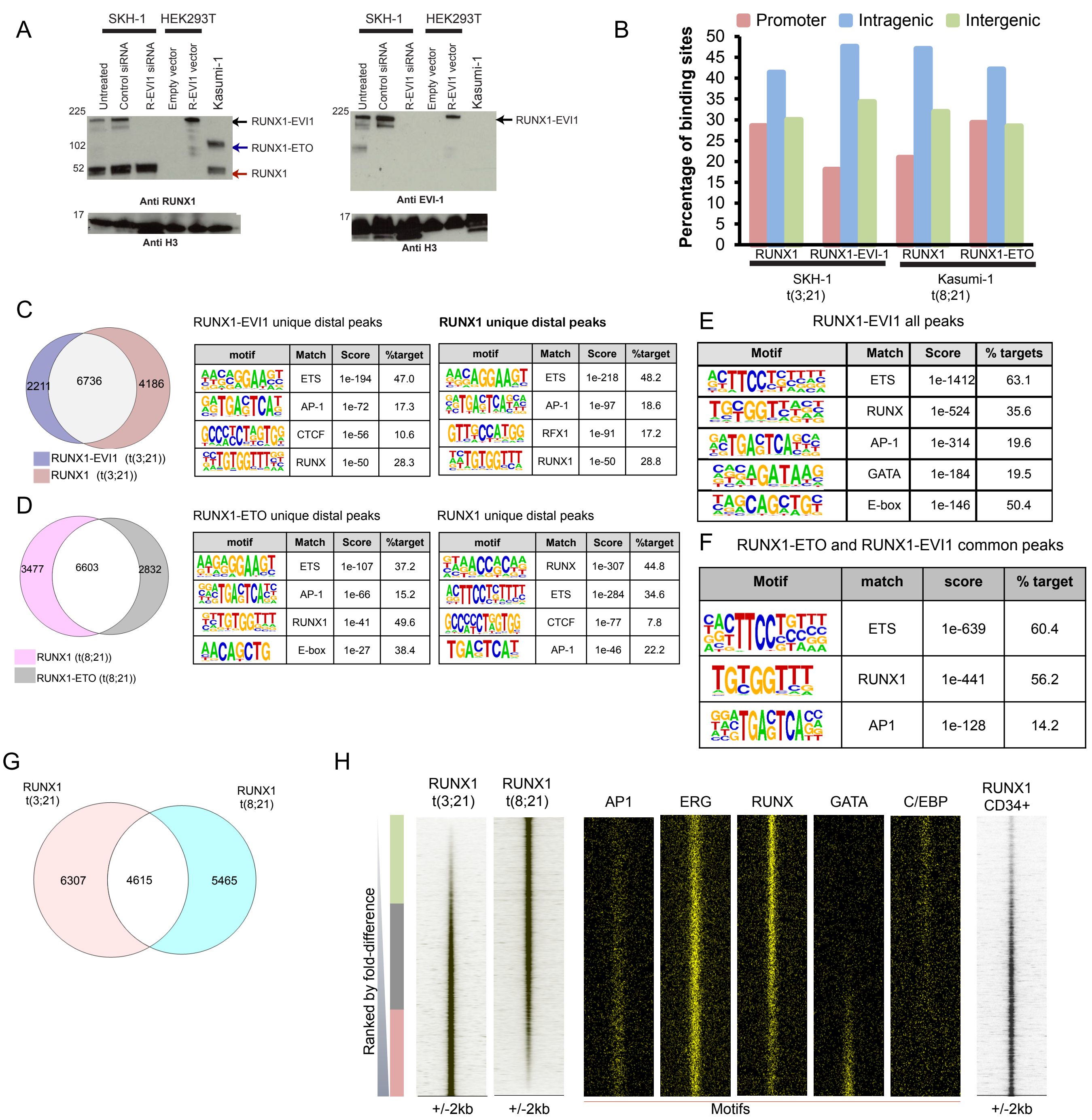

Supplemental Figure 2 (related to Figure 2)

A) Western blot analyses of nuclear extracts from: t(3;21) SKH-1 cells (untreated or transfected with control siRNA or RUNX1-EVI1 siRNA), HEK293T cells (transfected with either empty vector or RUNX1-EVI1 vector as size control), and t(8;21) Kasumi-1 cells. Sizes in kDalton are shown on the left of the blot. Western were blots probed with either an anti-EVI1 or an anti-RUNX1 (N-terminal epitope antibody). Anti-EVI1 and anti-RUNX1 (N-terminal epitope) antibody labels a RUNX1-EVI1 specific band (black arrow). This band is absent in SKH-1 transfected with a RUNX1-EVI1 specific siRNA and is present in HEK293T cells only when transfected with a RUNX1-EVI1 plasmid. In Kasumi-1 nuclear extract, EVI1 antibody does not detect anything, whilst the N-terminal RUNX1 antibody detects RUNX1-ETO (which

is smaller than RUNX1-EVI1) (blue arrow). The 52 kDa band is wild type RUNX1 (red arrow). Anti-H3 antibody was used as a loading control.

B) Percentage of binding sites within promoter, intragenic and intergenic region for RUNX1 and EVI1 (RUNX1-EVI1) ChIP-Seq in t(3;21) SKH-1 and RUNX1 and ETO (RUNX1-ETO) ChIP-Seq in t(8;21) Kasumi-1 cells.

C-D) Venn diagrams of peak overlap between ChIP-seq experiments with tables showing de novo motif analyses in distal peaks unique to each ChIP-Seq data set: C) RUNX1-EVI1 vs RUNX1 in t(3;21) SKH-1; D) RUNX1 vs RUNX1-ETO in t(8;21) Kasumi-1.

E) De novo motif analyses in all RUNX1-EVI1 binding sites using HOMER

G) Overlap between RUNX1 peaks between t(3;21) and t(8;21) cells.

H) RUNX1 peaks in t(3;21) cells were ranked according to tag count and RUNX1 peaks from t(8;21) were plotted alongside, together with the motifs for the indicated transcription factors on the right. To the far right RUNX1 peaks in normal CD34+ peripheral blood stem cells are shown..

Figure S3

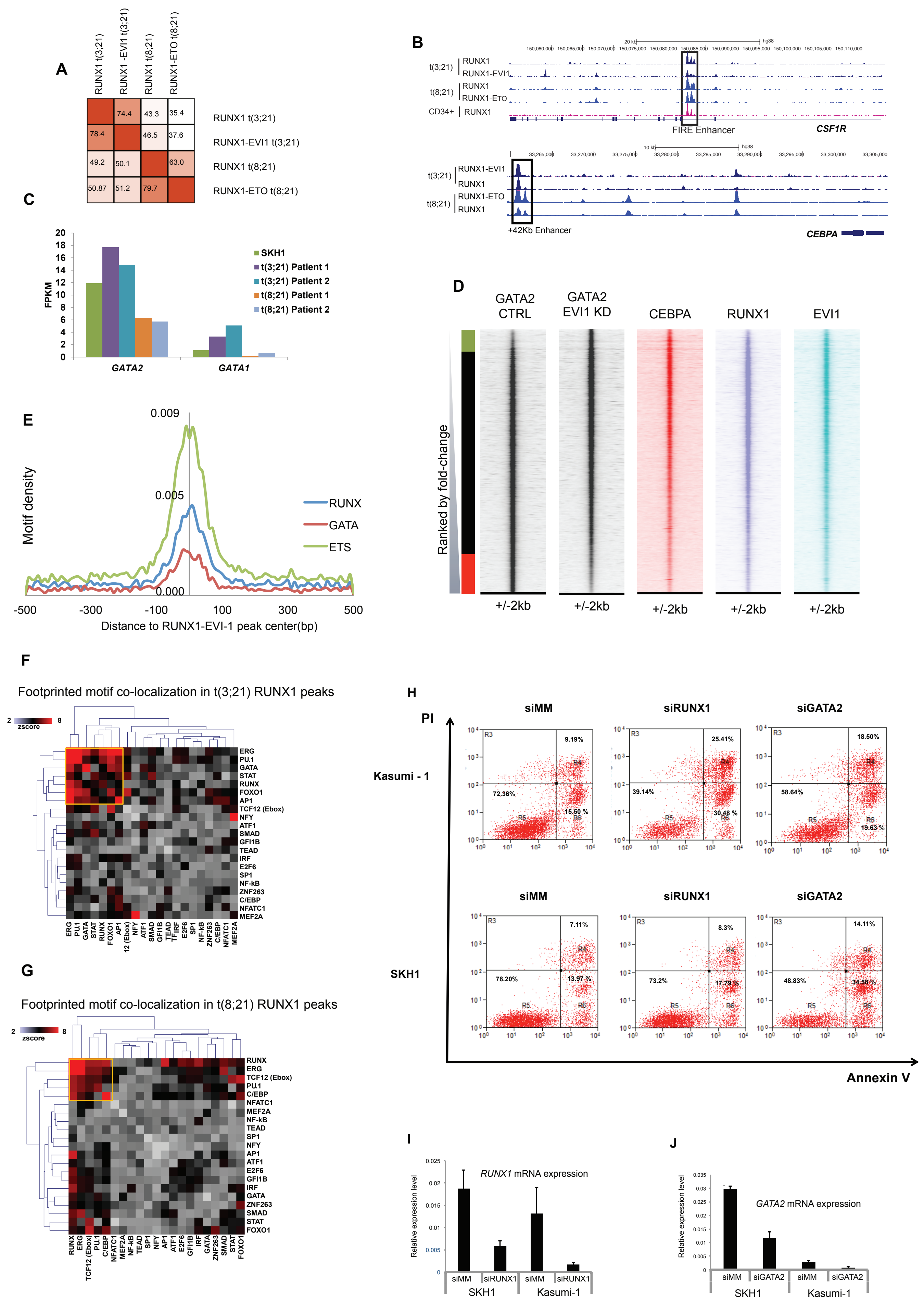

Supplemental Figure 3 (related to Figure 3)

A) Percentage of binding sites within promoter, intragenic and intergenic regions for RUNX1 and EVI1 (RUNX1-EVI1) ChIP-Seq in t(3;21) SKH1 cells and RUNX1 and ETO (RUNX1-ETO) ChIP-Seq in t(8;21) Kasumi-1 cells.

B) UCSC browser screen shot of the *CSF1R* locus. ChIP-Seq experiments were performed measuring RUNX1 and RUNX1-EVI1 binding in t(3;21) SKH-1 cells, RUNX1 and RUNX1-ETO binding in t(8;21) Kasumi-1 cells, and RUNX1 binding in normal CD34+ PBSC. The enhancers at *CSF1R* bind RUNX1 in both t(3;21) and t(8;21) cells, as well as RUNX1-EVI1 or RUNX1-ETO, respectively.

C) Expression of GATA2 and GATA1 based on FPKM values from RNA-Seq in SKH-1 (average of independent replicates), t(3;21) and t(8;21) patients (two patients for each CBF leukemia).

D) ChIP-Seq profiles spanning 4 kb windows for GATA2 ChIP-Seq peaks present in SKH-1 cells after either control siRNA or RUNX1-EVI1 siRNA treatment. Peaks are ranked from top to bottom in order of increasing relative DNA sequence tag count for peaks identified after control siRNA or RUNX1-EVI1 siRNA treatment in SKH-1. The boxes indicate GATA2 binding sites specific to control siRNA treated cells (bottom) or to RUNX1-EVI1 siRNA treated cells (top). To the right are C/EBPα, RUNX1 and RUNX1-EVI1 ChIP-Seq profiles in SKH-1 cells aligned to the same coordinates.

E) Average profiles of the distribution of the indicated binding motifs around the RUNX1-EVI1 binding peak center in a population of sequences bound by RUNX1-EVI1, RUNX1 and GATA2.

F, G) Bootstrap sampling analyses of DNase I footprinted motifs in RUNX1 binding sites from t(3;21) or t(8;21) leukemia. RUNX1 binding sites from ChIP-seq in t(3;21) SKH-1 cells (F) and t(8;21) Kasumi-1 cells (G) mapped onto t(3;21) patient 2 or t(8;21) patient 1, respectively. Motif footprinting probabilities from either t(3;21) patient 2 or t(8;21) patient 1 at these sites. The heatmap shows the probability of footprinted motifs co-localizing within a window of 50 bp, at RUNX1 binding sites, in each leukemia, as compared to sampling by chance alone. Enrichment (red) of GATA, AP-1, ERG and PU.1 occupied motifs is found at RUNX1 bound sites in t(3;21) patient samples (F). In contrast, enrichment (red) of RUNX, ERG and CEBP footprinted motifs at RUNX1 bound sites is found in t(8;21) patient samples (G).

H) Representative FACS panels of 3 independent experiments measuring Annexin V/PI staining on Kasumi-1 and SKH-1 cells after 5 days of knockdown with control siRNA (siMM), RUNX1 and GATA2 specific siRNAs in Kasumi-1 cells (upper panels) or SKH-1 cells (lower panels).

I) *RUNX1* mRNA levels after RUNX1 knockdown in the indicated cell types, relative to *GAPDH* expression. Graph of mean and error bars indicate the SD between three independent experiments.

J) *GATA2* mRNA levels after GATA2 knockdown relative to *GAPDH* expression in the indicated cell types. Graph of mean and error bars indicate the SD between three independent experiments.

Figure S4

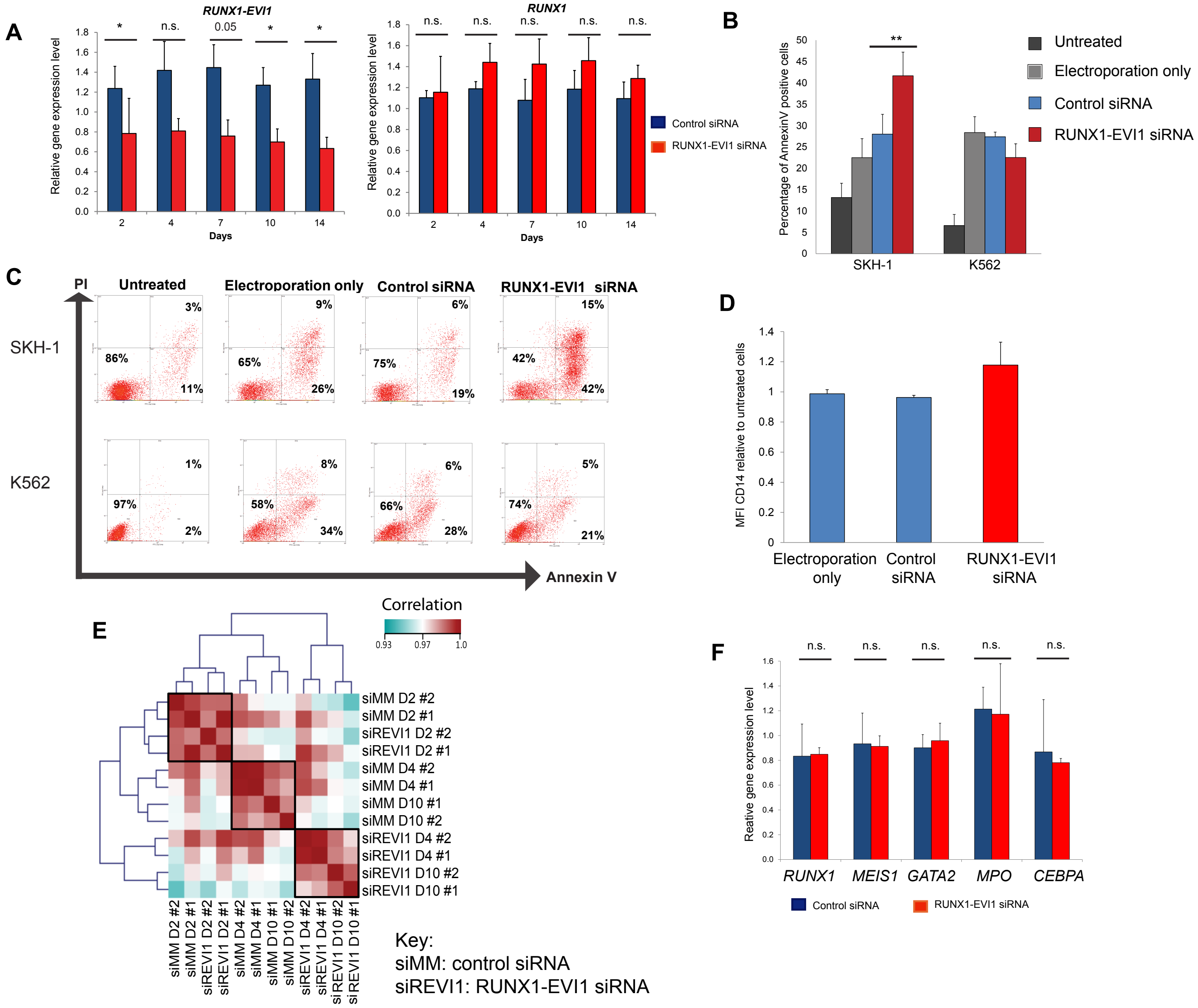

Supplemental Figure 4 (related to Figure 4)

A) *RUNX1-EVI1* mRNA decreases in SKH-1 cells treated with RUNX1-EVI1 siRNA, as compared to control siRNA treatment. *RUNX1* mRNA levels are unaffected by RUNX1-EVI1 siRNA as compared to control siRNA. RT-qPCR showing mRNA levels relative to GAPDH and normalized to untreated cells. *RUNX1-EVI1* or *RUNX1* mRNA levels in SKH-1 transfected with either specific RUNX1-EVI1 siRNA or control siRNA. Graph of mean and SEM of 4 independent experiments. n.s. not significant, * $p<0.05$ by unpaired t-test.

B-C) Treatment with RUNX1-EVI1 siRNA results in increased apoptosis of SKH-1 but not K562 cells. Annexin V FITC and PI staining in SKH-1 and K562 cells either untreated or

after 14 days with electroporation alone or, either, control siRNA or RUNX1-EVI1 siRNA transfection. B) Percentage of Annexin V stained cells. Graph of mean and SEM of at least 3 independent experiments. ** denotes p<0.01 by paired t-test. Representative flow cytometry plots shown in C).

D) CD14 expression on SKH-1 cells is unchanged following RUNX1-EVI1 knockdown. Flow cytometric analysis of t(3;21) SKH-1 cells stained with CD14-FITC: untreated, with either electroporation alone or, control or RUNX1-EVI1 siRNA transfection. MFI (median) of CD14 FITC relative to untreated SKH-1 after14 days of treatment. Graph of mean and SEM of 6 independent experiments.

E) Clustering analysis of RNA-Seq data from of SKH-1 treated with either control siRNA or RUNX1-EVI1 siRNA. RNA was extracted either after 2, 4 or 10 days of treatment as indicated. Hierarchical clustering of Pearson correlation coefficient between two biological replicates (#1 and #2) of each treatment conditions (siMM: control siRNA; siREVI1: RUNX1-EVI1 siRNA) was performed. Independent replicates cluster together but there are also three major clusters: samples from day 2 time point (both control siRNA and RUNX1-EVI1 siRNA treated samples), samples from control siRNA treated samples at day 4 and 10, and samples from RUNX1-EVI1 siRNA treated samples at day 4 and day 10.

F) RT-qPCR measurements of mRNA levels of *MPO*, *RUNX1, CEBPA*, *GATA2* and *MEIS1*, relative to GAPDH and normalized to untreated cells are unchanged in K562 cells after RUNX1-EVI1 siRNA as compared to control siRNA transfection. Graph of mean and SEM of 3 independent experiments. n.s. not significant by unpaired t-test.

Figure S5

A

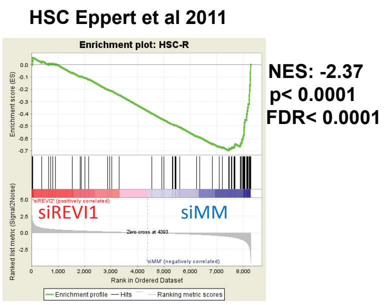

B

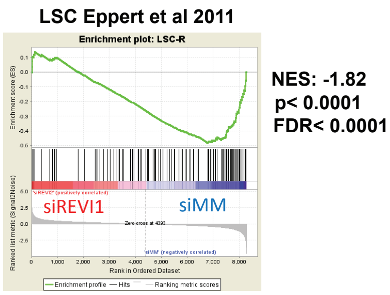

C

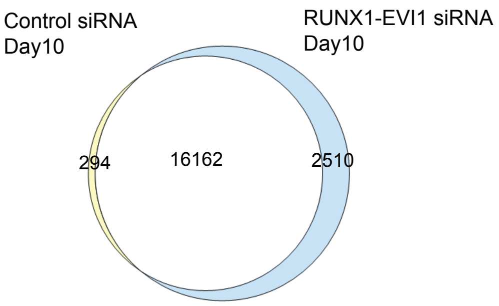

D

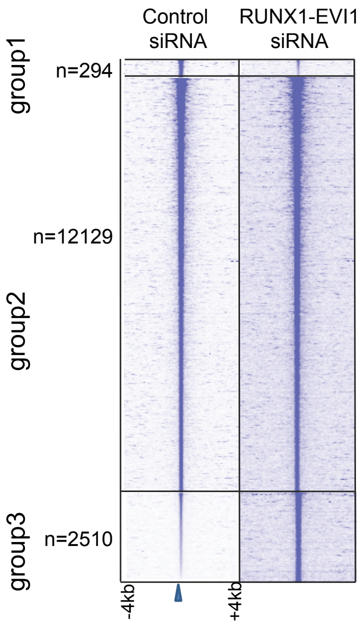

E

De novo motifs (group2)

Motif	match	P-value	%target
	ETS	1e-499	32.8
	AP1	1e-301	20.2
	CTCF	1e-225	13.7
	RUNX	1e-195	32.6
	E-box	1e-46	39.7
	GATA	1e-30	9.0

De novo motifs (group3)

Motif	match	P-value	%target
	ETS	1e-318	42.3
	C/EBP	1e-115	29.5
	RUNX	1e-98	24.5
	AP1	1e-98	18.5
	CTCF	1e-59	9.6

F

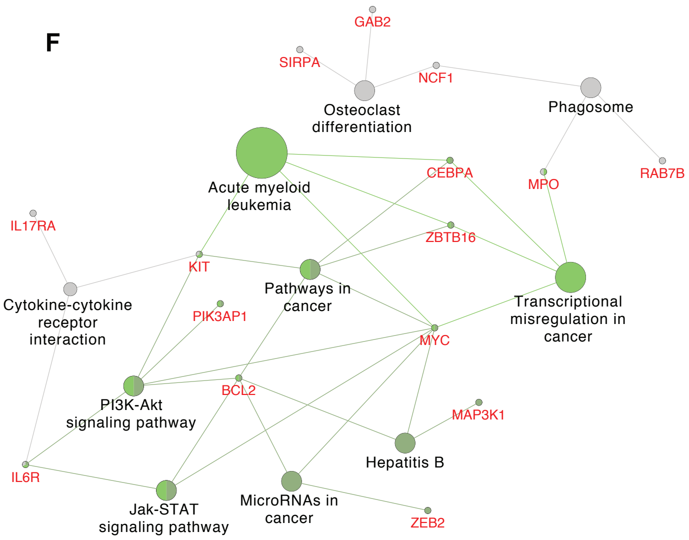

Up-regulated RUNX1-EVI1 target genes
responding to RUNX1-EVI1 knock-down

Supplemental Figure 5 (related to Figure 5)

A, B) Gene set enrichment analysis (GSEA) based on RNA-Seq from SKH-1 cells after 10 days of treatment with either RUNX1-EVI1 siRNA (siREVI1) or control siRNA (siMM). Loss of enrichment of gene set associated with either hematopoietic (A) or leukaemic stem cells (LSC, B) when SKH-1 were treated with RUNX1-EVI1 siRNA.

C) Venn diagram of the overlap of DHS peaks showing that knock-down of RUNX1-EVI1 for 10 days leads to the generation of 2510 new DHSs.

D) Heatmap ranking according to sequence tag counts showing DHS profiles after either control siRNA or RUNX1-EVI1 siRNA transfection. Group 1 – 3 identify peaks which are control siRNA-specific, unchanged or RUNX1-EVI1 siRNA specific, respectively.

E) Enriched transcription factor binding motifs in group 2 and 3 peaks defined in (D) highlighting the appearance of C/EBP motifs after RUNX1-EVI1knock-down.

F) KEGG pathway for RUNX1-EVI1 ChIP-seq target genes whose expression is upregulated at least 1.5 fold, between RUNX1-EVI1 siRNA and control siRNA treated cells (10 days of treatment), as identified by RNA-seq.

Figure S6

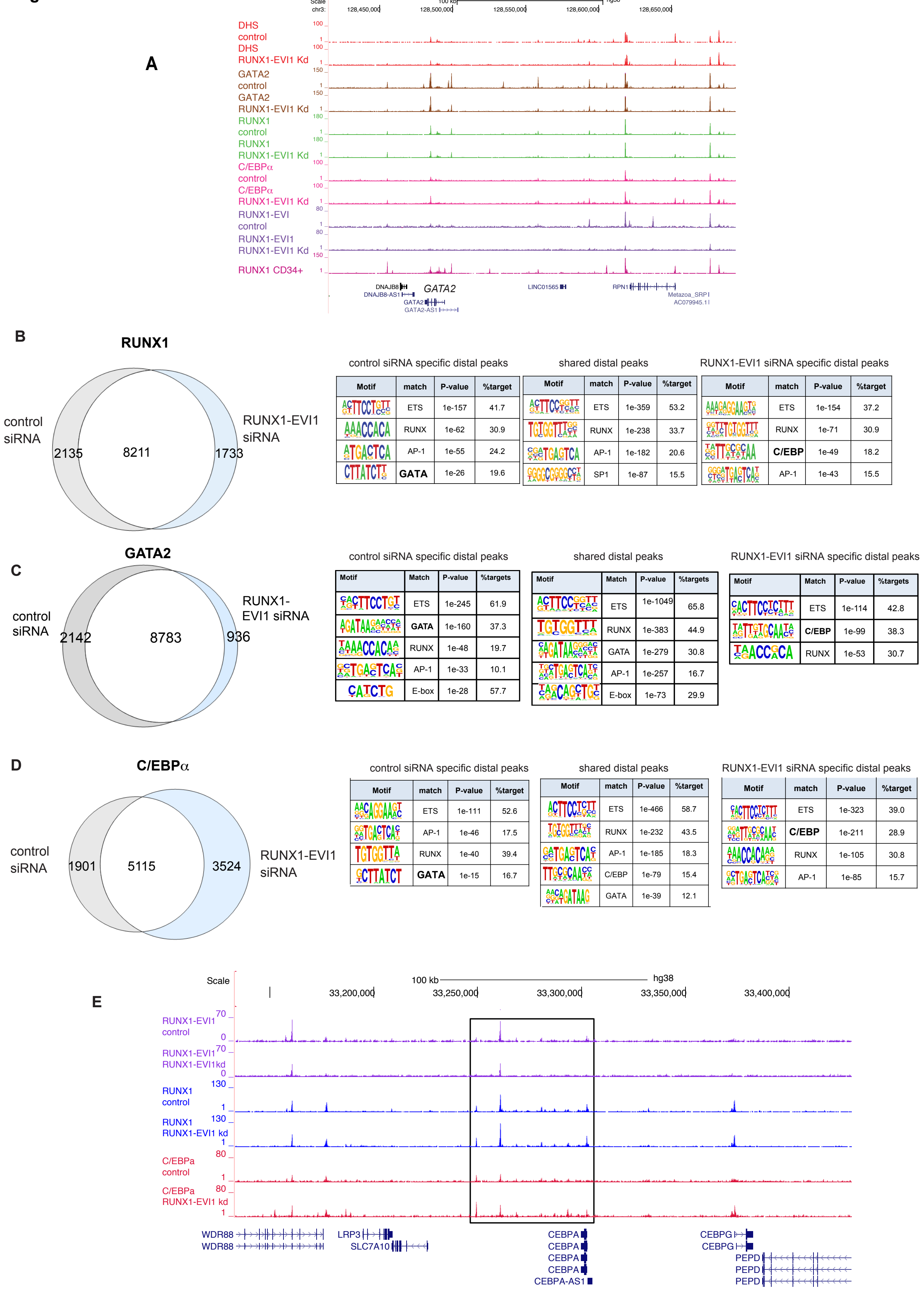

Supplemental Figure 6 (related to Figure 6)

A) UCSC genome browser screen shot of RNA-Seq, DNase-Seq and RUNX1, RUNX1-EVI1 and C/EBPα ChIP-seq 10 days after either control siRNA or RUNX1-EVI1 siRNA treatment at *GATA2.* At *GATA2*, C/EBPα binding increases following RUNX1-EVI1 siRNA transfection.

B) Analyses of RUNX1 ChIP-Seq peaks: GATA motif-containing RUNX1 binding sites are found only in SKH-1 treated with control siRNA whereas CEBP motif-containing RUNX1 ChIP-seq peaks are found only in SKH-1 treated with RUNX1-EVI1 siRNA. Left panel: Venn diagram showing overlap of RUNX1 ChIP-seq peaks in SKH-1 treated with control siRNA with RUNX1 ChIP-seq peaks in SKH-1 treated with RUNX1-EVI1 siRNA (after 10 days treatment). The tables show de novo motif analyses of distal sites found only in SKH-1 cells treated with control siRNA (left), or RUNX1-EVI1 siRNA (right), or found in both control and RUNX1-EVI1 siRNA treated SKH-1 (middle).

C) Analyses of GATA2 ChIP-Seq peaks: Loss of some GATA2 binding sites following RUNX1-EVI1 knockdown in SKH-1 cells. Left panel: Venn diagram showing overlap of GATA2 ChIP-seq peaks in SKH-1 treated with control siRNA with GATA2 ChIP-seq peaks in SKH-1 treated with RUNX1-EVI1 siRNA (after 10 days treatment). The tables shows de novo motif analyses of distal sites found only in SKH-1 treated with control siRNA (left), or RUNX1-EVI1 siRNA (right), or found in both control and RUNX1-EVI1 siRNA treated SKH-1 (middle).

D) Analyses of C/EBPα ChIP-Seq peaks: GATA motif containing sites found in control siRNA but not RUNX1-EVI1 siRNA treated SKH-1 C/EBPα binding sites. Conversely CEBP motif containing sites most commonly found in RUNX1-EVI1 siRNA, but not control siRNA treated SKH-1 C/EBPα binding sites. Left panel: Venn diagram showing overlap of C/EBPα ChIP-seq peaks in SKH-1 treated with control siRNA with C/EBPα ChIP-seq peaks in SKH-1 treated with RUNX1-EVI1 siRNA (after 10 days treatment). The tables show de novo motif analyses of distal sites found only in SKH-1 treated with control siRNA (left), or RUNX1-EVI1 siRNA (right), or found in both control and RUNX1-EVI1 siRNA treated SKH-1 (middle).

(E) UCSC browser screenshot depicting transcription factor binding (RUNX1-EVI1, RUNX1 and C/EBPα) to the regulatory elements of the *CEBPA* locus (boxed in), without (control) and with RUNX1-EVI1 knock-down.

Figure S7

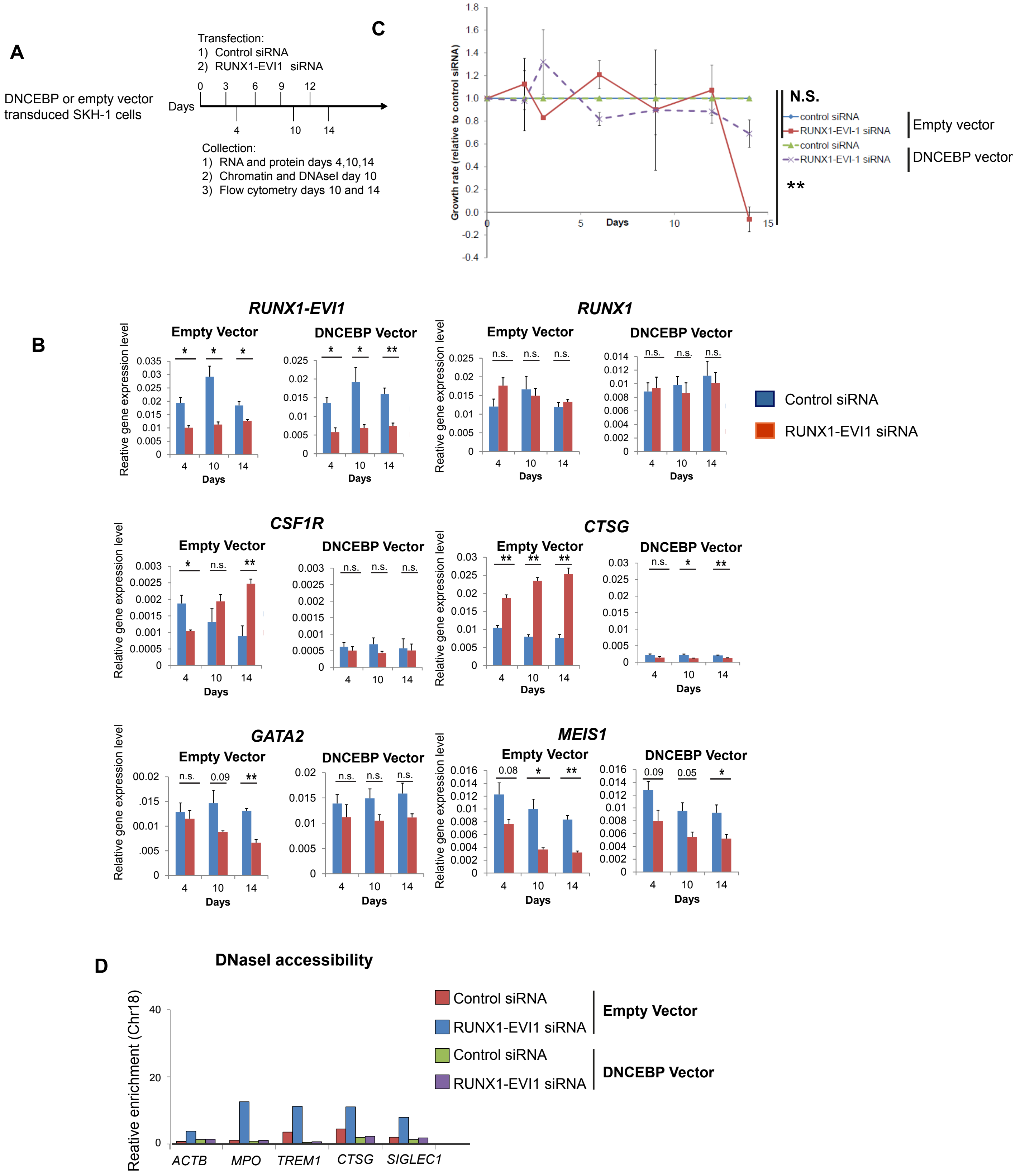

Supplemental Figure 7 (related to Figure 7)

A) Experimental scheme of RUNX1-EVI1 knockdown in either empty vector or DNCEBP vector transduced SKH-1 cells. Transfection with either control siRNA or RUNX1-EVI1 siRNA on days 0, 3, 6, 9 or 12 in both empty vector transduced SKH-1 or DNCEBP transduced SKH-1. Cells collected for RNA, chromatin, DNase I or flow cytometry on designated days.

B) mRNA levels as measured by RT-qPCR, relative to GAPDH after control or RUNX1-EVI1 siRNA transfection (4, 10 or 14 days of treatment). RUNX1-EVI1 or RUNX1 mRNA levels in either empty vector or DNCEBP vector transduced SKH-1. Decrease in *RUNX1-EVI1* mRNA levels in both empty or DNCEBP vector transduced SKH-1transfected with RUNX1-EVI1 siRNA, but *RUNX1* mRNA levels remain the same. *CTSG* and *CSF1R* mRNA levels increase in empty vector transduced SKH-1 following RUNX1-EVI1 knockdown but not in DNCEBP vector transduced SKH-1 treated in an identical manner. *GATA2* and *MEIS1* mRNA levels decrease in empty vector transduced SKH-1 following RUNX1-EVI1 knockdown. *GATA2* and *MEIS1* mRNA levels also decrease in DNCEBP vector transduced SKH-1 following RUNX1-EVI1 knockdown, but this decrease is reduced. n.s. not significant, * $p<0.05$ ** $p<0.01$ by unpaired t-test

C) Growth rates of empty (solid line) or DNCEBP vector (dashed line) transduced SKH-1 cells, transfected with RUNX1-EVI1 siRNA, relative to control siRNA treatment. Mean and SEM of 3 independent experiments. ** denotes $p<0.01$ by paired t-test between control and RUNX1-EVI1 siRNA transfected empty vector transduced SKH-1 cells, after 14 days of siRNA treatment. Differences between growth rates of DNCEBP transduced SKH-1 after RUNX1-EVI1 siRNA and control cells by paired t-test. (n.s.): not significant.

D) DNase I accessibility measurement using qPCR at the *MPO* and *SIGLEC1* enhancer, and the *TREM1* and *CTSG* promoter. DNase I digests were performed on empty vector and DNCEBP vector transduced SKH-1 cells following either control or RUNX1-EVI1 siRNA transfection. DNase I accessibility increases in empty vector transduced SKH-1 following RUNX1-EVI1 knockdown, but not in DNCEBP vector transduced SKH-1 cells treated identically. Primers amplifying sequences within the *ACTB* gene body are shown as negative control. Enrichment was normalized relative to chromosome 18. A 2nd independent experiment is shown in the main figure 7H.

Supplemental Experimental Procedures

Cell line culture

Cells were maintained in a humidified incubator at 37°C with 5% CO2. t(3;21) SKH-1 and K562 cells were cultured in RPMI medium with 10% fetal calf serum (FCS) supplemented with glutamine and penicillin/streptomycin. t(8;21) Kasumi-1 cells were cultured in RPMI with 15% FCS supplemented with glutamine and penicillin/streptomycin. HEK293T and HeLa cells were cultured in Dulbecco Modified Eagle Media (DMEM) with 10% FCS supplemented with glutamine and penicillin/streptomycin.

Purification of blood samples from patients with AML

Blood from t(3;21) patient 1 was diluted 1:1 with PBS and layered onto density gradient medium (Lymphoprep Stem Cell technology, USA). The blood-PBS-lymphoprep mix was subsequently centrifuged at 592xg (acceleration setting 4, no brakes). After centrifugation, the mixture had separated into two phases with the mononuclear cells separated into a layer in between. This middle layer was then isolated and incubated with CD34+ microbeads (Miltenyi-Biotech, USA), according to the manufacturer instructions. The CD34+ expression on this fraction was confirmed by flow cytometry before either immediately use for DNase I hypersensitivity site mapping or RNA extraction by Trizol, as described below.

Cells from t(3;21) patient 2 was previously isolated by density gradient medium and cryopreserved at the Erasmus University Medical Centre, Netherlands. CD34+ cells were thawed with pre-warmed RPMI-1640 +10%FCS. After centrifugation, the cell pellet was re-suspended in 750 µl MACS buffer (PBS with 0.5% BSA and 2 mM EDTA) and 35 µl CD34-PE, with a separate sample stained with IgG PE as an isotype control. CD34+ cells were isolated by FACS using a MoFlo Astrios (Beckman Coulter, USA) and were then directly used for DNase I hypersensitivity site mapping or underwent RNA extraction by Trizol, as described below.

Purification of CD34+ mobilized peripheral blood stem cells

CD34+ peripheral blood stem cells (PBSCs) from healthy adults identified by the NHS Blood and Transplant service (NHSBT) were mobilized into the peripheral circulation by administrating donors with pegylated G-CSF (trade name: Lenograstim, Chugai Pharmaceuticals, Japan). Cells were harvested from the patients by apheresis and stored by NHSBT in liquid nitrogen. Cryopreserved cells were thawed at 37ºC using a waterbath and eluted from storage bag with a PBS/Glucose/Citrate solution (0.09% glucose + 3.3% FCS + 1mM sodium citrate). After centrifugation at 300xg for 5 minutes, the cell pellet was treated with DNase I (Roche, Switzerland) at 0.6mg/ml concentration in PBS/Glucose/Magnesium/Calcium solution (PBS+0.5 mM $MgCl_2$ + 1.2 mM $CaCl_{2)}$ + 1 % FCS + 0.1 % glucose + 2 mM $MgCl_2$) for 5 minutes at room temperature. Following DNase I treatment the cells were once again diluted with PBS/Glucose/Citrate solution and the mononuclear isolated by density gradient medium and CD34+ beads separation by MACS columns, as described above.

siRNA mediated depletion of RUNX1-EVI1, RUNX1 and GATA2

$1x10^7$ Kasumi-1 or SKH1 cells were electroporated using a EPI 3500 (Fischer, Germany) electroporation at 350v, 10ms. siRNA sequences (Axolabs, Germany) specific for the translocation breakpoint of Runx1-EVI1 were 5'-GAACCUCGAAAUAAUGAGUGU-3' (sense) and 5'-ACUCAUUAUUUCGAGGUUCUC-3' (antisense). Control siRNA was 5'-CCUCGAAUUCGUUCUGAGAAG-3' (sense) with 5'-UC UCAGAACGAAUUCGAGGUU-3' (antisense). siRNA was used at 200 nM. GATA2 siRNAs (3) were: GATA2 ID 2624, Trilencer-27 Human siRNA, Origene Technologies, Inc. and RUNX1 siRNAs (4) were: ON-TARGETplus RUNX1 siRNA, Dharmacon. After electroporation, the cells remained in their cuvettes for 5 minutes before being directly added to RPMI-1640 with 10% FCS, supplemented with penicillin/streptomycin and glutamine at a concentration of 0.5×10^6 cells per ml and returned to an incubator kept at 37°C and 5% CO_2.

RNA extraction

Pelleted cells from primary patient material were lysed by adding 1ml Trizol ™ (Life Technologies, US). 200 µl of chloroform was added and the mixture was manually shaken for 15 seconds. The mixture was incubated at room temperature for 3 minutes. The mixture was centrifuged at 12000 x g for 15 minutes at 4°C. The top clear aqueous phase was removed and placed in a fresh tube. 0.5 ml of 100% isopropanol was added to the isolated aqueous phase and incubated at room temperature for 10 minutes which was then transferred to a RNeasy MinElute column (Qiagen, USA) and centrifuged for 15 s at 8000xg. 350 µl of RWI buffer from the RNeasy Kit was added to the column and centrifuged for 15 s at 8000xg. 10 µl DNase I and 70µl RDD buffer (Qiagen, USA) were mixed and added to the column and incubated for 15 minutes at room temperature. Afterwards, 350 µl of RWI buffer from RNeasy Kit (Qiagen, USA) was added to the column and centrifuged for 15s at 8000xg. Following this 500 µl of RPE buffer was added and centrifuged for 15 s at 8000xg. The column was washed with 500 µl 80% ethanol and centrifuged at 2 minutes at 8000xg. The column was dried by centrifuged at 5 minutes at 8000xg. RNA was eluted from the column by adding 12 µl of water to the column followed by centrifugation at 5 minutes at 8000xg.

RNA was isolated from SKH-1 cells by Trizol™ (Life Technologies, US) as by manufacturer's instructions. At the last step of the protocol RNA was resuspended in 17 µl of RNAse free water to which was added 2 µl of 10x buffer supplied with the Ambion Turbo DNase I (Thermos Scientific, USA), of which 1 µl was added. All of which was incubated at 37ºC for 30 minutes. The RNA solution was then purified using a Nucleospin RNA clean up column (Machery Nagel, France), according to their instructions. The quality of RNA from all methods was assessed using a spectrophotometer, by the ratio of the absorbance at 260 nM and 280 nM wavelengths. RNA has a greater absorbance in the 260 nM wavelength, Eukaryotic Total RNA PICO Bioanalyser chip (Agilent technologies, USA) allows visualisation of the size of the RNA molecules and thus, demonstrates whether the sample is degraded or not.

RNA Seq libraries

RNA-Seq libraries were prepared with a Total RNA Ribo-zero library preparation kit (with ribosomal RNA depletion) (Illumina, USA) according to manufacturer's instructions with the following alterations: 15 cycles of PCR was undertaken to amplify the library and adaptors for multiplexing were used at a 1:4 dilution. Library quality was checked by running the samples on a Bioanalyser and libraries were quantified using a Kapa library quantification kit

(Kapa Biosystems, USA) and run in a pool of eight indexed libraries in two lane of a HiSeq 2500 (Illumina, USA) using rapid run chemistry with 100bp paired end reads.

cDNA synthesis

1µg RNA was used to make cDNA with 0.5 µg OligoDT primer, Murine Moloney Virus reverse transcriptase and RNase Inhibitor (Promega, USA) according to the manufacturer's protocol.

Real-time polymerase chain reaction

RT-PCR was performed using Sybr Green mix (Applied Biosystems, UK), at 2x dilution. Primers were used at 100 nM final concentration. cDNA was diluted either 1:10 or 1:50 depending on expression levels of targets. A 7900HT system (Applied Biosystems, UK) was used to perform qPCR. Analyses were performed in technical duplicates using a standard curve derived from RNA purified from the untreated cell line (1:10 followed by 1:5 dilutions). Primer sequences are listed in the Appendix.

Dead cell removal and Annexin V/PI staining for flow cytometry

Dead cell removal was performed using negative selection on a MS column following incubation with Dead Cell Removal microbeads (Mitenyi Biotech, USA) as per manufacturer's instructions. Dead cell removal was performed on all samples prior to RNA extraction or DHSs mapping. Annexin V-APC/PI staining (Ebiosciences, USA) or Annexin V-FITC/PI staining (BD Biosciences, USA) was performed according to manufacturer's instructions. Annexin V-APC staining was used for cells that expressed GFP. FACS data were analyzed by Summit 4.3 software (Beckman Coulter).

DNaseI hypersensitivity site mapping

Prior to DNaseI digestion, apoptotic cells were removed using the Dead Cell Removal Kit (Miltenyl Biotech, UK) as per manufacturer's instructions. 3x 10^7 SKH-1 cells were suspended in 1 ml DNase I buffer (0.3M sucrose, 60 mM KCl, 15 mM NaCl, 5 mM MgCl2, 10 mM Tris pH7.4). Digestion on $4.5x10^6$ cells was performed with DNase I (Worthington, DPPF grade) at 80 units/ml in DNase I buffer with 0.4% NP-40 and 2 mM CaCl2 at 22ºC for 3 minutes. The reaction was stopped with cell lysis buffer (0.3M NaAcetate, 10mM EDTA pH 7.4, 1% SDS) with 1mg/ml Proteinase K and incubated at 45ºC overnight.

For DHS mapping in CD34+ purified t(3;21) patient cells and in SKH-1 transfected with siRNA, lower cell numbers were available and therefore the DNase I concentrations were reduced according to the cell numbers available.

The digested DNase I material was treated with RNAse A (Sigma Aldrich, Germany) at a final concentration of 100 µg/ml at 37ºC for 1 hr. Genomic DNA was extracted using phenol/chloroform method: an equal volume of phenol was added to the reaction and placed on a rotator wheel for 45 minutes. This was centrifuged for 5 minutes at 16000 x g at room temperature. The top layer was transferred to a new tube and the process was repeated sequentially with phenol/chloroform and chloroform. After purification by chloroform extraction, genomic DNA was precipitated with ethanol. This was pelleted by centrifugation for 5 minutes, at 16000 x g at 4°C. The pellet was resuspended with 70% ethanol and centrifugation for 5 minutes, at 16000 x g at 4°C. The pellet was air-dried and dissolved by Tris-EDTA (40 mM Tris Acetate 1 mM EDTA).

Digestion was checked visually by running the samples on a 0.7% agarose gel and by RT-PCR evaluating the ratio of open (TBP promoter) to closed regions of DNA (chromosome 18) and active gene body (beta-actin) to prevent selection of over digested samples (primers in Table S5B). Subsequently, between 2 to 10 µg of DNase I-digested DNA (depending on material available) were run on a 1.2% agarose gel for selection of shorter fragments to increase the fraction of fragments captured from DHSs. Prior to loading on gel, the purified DNA was treated again with RNAse A (Sigma Aldrich, USA) at a final concentration of 100 µg/ml at 37ºC for 1 hr. 50-300 bp fragments were isolated and purified from the gel using a MinElute gel extraction kit (Qiagen, USA) as per manufacturer's instructions and validated by qPCR as before (Figure 2-1 B-C). Following this, the size selected sample was validated again by RT-PCR, this time using shorter amplicons to enable detection of the shorter fragments enriched by the size selection process.

Library production of DNase I material for high throughput sequencing
After size selection, a library was prepared using Tru-seq DNA sample preparation kit (Illumina, USA) or MicroPlex library preparation kit v2 (Diagenode, Belgium) as per manufacturer's protocol. After PCR a final size selection step was performed by running the library on 1.5% TAE gel, followed by excision of 190-250 bp sized gel fragment. The library was purified from the gel using a MinElute gel extraction kit (Qiagen, USA).

The quality of the libraries was assessed on an Agilent 2100 Bioanalyser. Libraries were subsequently run on two lanes of an Illumina HiSeq 2500 flow-cell for transcription factor footprinting, or as part of 12 indexed libraries in one lane of a NextSeq500 (Illumina, USA) for DHS mapping alone.

ChIP-qPCR and ChIP-Seq library preparation

Double cross-linking
A double cross-linking technique was used to optimize the efficiency of transcription factor chromatin immunoprecipitation (ChIP). $2x10^7$ cells were washed thrice in PBS. Di(N-succinimidyl) glutarate (DSG) (Sigma-Aldrich, Germany) at 850 µg/ml was added to 2x106 cells per ml and were incubated for forty-five minutes. Cells were washed four times and fixed with 1% formaldehyde (Pierce, Thermos Scientific, USA) for ten minutes. Glycine to produce a final concentration of 100mM was added to stop the reaction. The pellet was washed again with PBS. Buffer A (HEPES pH 7.9 10 mM, EDTA 10 mM, EGTA 0.5 mM, Triton x100 0.25%, complete mini protease inhibitor cocktail (PIC) 1x (Sigma-Aldrich, Germany) was added for 10 mins at 4°C and removed by centrifugation at 500 g for 5 minutes. This was repeated with buffer B (HEPES pH 7.9 10 mM, EDTA 1 mM, EGTA 0.5 mM, Triton x100 0.01%, PIC 1x). The residual nuclei were then spun down at 16000 x g at 4°C for 5 minutes and aliquoted at $2x10^7$ cells for 4 immunoprecipitations.

Chromatin immunoprecipitation (ChIP)
Each aliquot of $2x10^7$ cells was re-suspended in 600 µl of sonication buffer (Tris-HCL pH 8 25 mM, NaCL 150 mM, EDTA 2 mM, Triton 100x 1%, SDS 0.25%, Protease inhibitor cocktail (PIC) 1x). 300 µl of nuclei in sonication buffer was placed in each polystyrene tube and sonicated at 75% amplitude, 26 cycles: 30s on and 30s off per cycle (Q800, Active Motif, USA). Subsequently, 1.2ml of dilution buffer (Tris-HCL pH8 25 mM, NaCL 150 mM, EDTA 2 mM, Triton 100x 1%, glycerol 7.5%, PIC 1x) was added to the pooled post sonication

material. This was divided equally between four immunoprecipitations (with 5% of input taken for validation).

15 µl protein G beads (Diagenode, Belgium) were washed twice with 500 µl of 50 mM citrate phosphate buffer and once with 100 mM sodium phosphate). 2 µg antibody (EVI1, C50E12, Cell Signalling, lot 3; or RUNX1, Ab23980, Abcam lot 144722) or 4µg antibody (C/EBPα, A2814 Santa Cruz) was added to 10 µl 100 mM sodium phosphate, 0.5% BSA and incubated with protein G beads at 4°C for 1 hour. Chromatin was then added to the protein G beads with antibody and returned to 4°C for 4 hours. Unbound chromatin was separated from the beads by magnet and the attached beads were washed by buffer 1 (Tris HCL 20 mM, NaCl 150 mM, EDTA 2 mM, Triton x100 1%, SDS 0.1%), twice with buffer 2 (Tris HCL 20 mM, NaCl 500 mM, EDTA 2 mM, Triton x100 1%, SDS 0.1%), LiCL buffer (Tris HCL 10 mM, LiCl 250 mM, EDTA 1 mM, NP40 0.5%, sodium deoxychlolate 0.5%) and finally twice with wash buffer 4 (Tris HCL pH8, 10 mM, NaCl 50 mM, EDTA 1mM). The column was eluted twice with 50 µl buffer (NaHCO3 100 mM and SDS 1%) and the eluant containing the chromatin was pooled. Crosslinks were reversed by incubating the samples at 65°C overnight in 500 mM NaCl, 500 µg/ml proteinase K. DNA was purified by Ampure beads (Beckman Coulter, USA), as above, with the DNA eluted with 50 µl water. Validation of the ChIP was performed by qPCR using a standard curve of genomic DNA from untreated SKH-1 cells (10ng/ µl followed by serial 1:5 dilutions). The input material was diluted 1:5 with water and qPCR was performed as above with primers listed in SI. Validation was analyzed as a ratio of the qPCR signal from the ChIP material over the input.

Library production of ChIP material for high throughput sequencing
Libraries for high throughput sequencing were prepared using the Tru-seq DNA sample preparation kit (Illumina, USA) or Kapa HyperPrep kit (Kapa Biosystems, USA), as per manufacturer's protocol. 18 cycles of PCR was performed and 200-350bp fragments were size selected by running the samples in an agarose gel. Libraries were purified from the gel using a MinElute Gel extraction kit (Qiagen, USA). Libraries were validated by qPCR, with an analysis of the ChIP signal of a positive control region (e.g. PU.1 3H enhancer) over a negative control region (e.g. *IVL*). Finally, libraries were quantified by Kapa library quantification kit (Kapa Biosystems, USA) and run in a pool of four indexed libraries in one lane of a HiSeq 2500 (Illumina, USA) or 12 indexed libraries in one lane of a NextSeq 500 (Illumina, USA) using 50 cycle single-end reads.

Retroviral production

Lenti- and retroviral transduction

pSIEW DNCEBP vector was generated by cloning the DNCEBP insert into the pHR-cppt-SIEW vector (Bomken et al., 2013). The DNCEBP insert was originally developed by Charles Vinson (NIH, USA) (Krylov et al., 1995). We used the pSIEW vector backbone (Empty and DNCEBP) to produce lentiviral particles. Packaging and envelope genes were on a separate plasmid to prevent further virus particle generation once transduced into the target cell.

Transfection of HEK293T cells for lentiviral production
HEK293T cells were re-plated 24 hour prior to transfection, so that at time of transfection they were 80-90% confluent. On the day of transfection TransIT-293T (Mirius, USA) was brought to room temperature. Trans-IT-293T forms a complex with DNA plasmids to enable

transfection into cells. A DNA mix was made from plasmids (Backbone vector containing transgene 30 µg, and the packaging vectors: Tat 1.2 µg, Rev 1.2 µg, Gag/Pol 1.2 µg, VSV-G 2.4 µg (gift from George Murphy, Boston, USA) (Sommer et al., 2009). For each 15 cm^2 2 ml of Optimem serum free media was mixed with 90 µl of TransIT-293 (Mirius, USA). This was allowed to rest at room temperature for 15 minutes. DNA mix was added to the TransIT-293 mixture and was left at room temperature for a further 15 minutes. Fresh DMEM with 10% FCS supplemented with glutamine and penicillin/streptomycin was exchanged with previous media on the HEK293T plates. The TransIT-293 – DNA mixture was then added drop-wise to the HEK293T plate. The viral supernatant was collected after 48 hours and subsequently every 12 hours for 36 hours.

Virus concentration

The virus concentration technique was the same for all viral particles. Viral supernatant was centrifuged at 1660xg 4°C 15 minutes to pellet cell debris. The supernatant was then filtered through a 0.45µm disc filter.

The viral supernatant was concentrated using a Centricon Plus 70 100 kDa filter (Millipore, USA), using the manufacturers instruction. The column was pre-rinsed with sterile water and the column centrifuged at 2000xg, 25 minutes at 4 degrees. The column was then inverted and the concentrate recovered by centrifugation at 1000xg for 2 minutes.

Lentiviral transduction of SKH-1

SKH-1 were transduced with viral concentrates with polybrene at 8 µg/ml by spinoculation at 1500xg for 2 hours at 32°C in non tissue culture treated plates. The plate was subsequently returned to the incubator overnight and at the next morning the viral media was removed and exchanged with fresh media. Viral transduction was estimated by eGFP percentage by flow cytometry 5 days after viral transduction. Cell sorting by FACS was undertaken by sorting for GFP positive cells.

Antibody staining for flow cytometry

$15x10^4$ were centrifuged at 300xg and washed with MACS buffer. The cell pellet was re-suspended in 50 µl MACS buffer and 2 µl of antibody was added and incubated for 15 minutes at 4ºC in the dark. After incubation, the cells was washed once with MACS buffer before resuspension in 300 µl MACS buffer and analyzed on Cyan ADP (Beckman Coulter, USA). Data was analyzed on Summit 4.3 (Beckman Coulter, USA). Antibodies used in this project are listed below.

Whole cell lysate preparation by RIPA buffer lysis

Whole cell lysate was made by lysing $5x10^6$ cells using RIPA buffer (50 mM Tris pH 7.4, 150 mM NaCl, 0.25% Na-deoxycholate, 1% NP40, 1 mM PMSF, complete mini protease inhibitor cocktail (PIC) 1x (Sigma-Aldrich, Germany)). After incubation on ice for 15 minutes, the sample was sonicated for 1 min using a Bioruptor (Diagenode, Belgium) at 4°C.

Nuclear extract

Nuclear extracts were prepared using a co-immunoprecipitation kit (Active Motif, USA). Protein extracts were quantified using Bradford protein reagent (Bio-Rad, USA) and 595nM absorbance quantified by spectrophotometry. Absolute concentrations were determined using a standard curve from a known concentration of BSA (Pierce, USA).

Western blotting

Cell lysate or nuclear extracts was run on an acrylamide gel and transferred to nitrocellulose membrane. This was probed with the antibodies described in the appendix. Enhanced chemiluminescence by SuperSignal PICO (Thermos Scientific, USA) was used to develop the membrane. Chemiluminescence was detected using either developer or Chemidoc XRS system (BioRad, USA).

Oligonucleotide sequences

Name	Forward	Reverse
**Quantitative PCR Primers used for DNA and RNA amplification**		
Actin (long)	GCAATGATCTGAGGAGGGAAGGG	GTGTCTTTCCTGCCTGAGCTGAC
Actin (short)	GCAATGATCTGAGGAGGGAAGGG	AGCTGTCACATCCAGGGTCCTCA
CEBPA	GAGGGACCGGAGTTATGACA	AGACGCGCACATTCACATT
CEBPB	GACAAGCACAGCGACGAGTA	CTCCAGGTTGCGCATCTT
Chromosome 18 (long)	ACTCCCCTTTCATGCTTCTG	AGGTCCCAGGACATATCCATT
Chromosome 18 (short)	AGGTCCCAGGACATATCCATT	GTTCAAATTGTGTTTTGTGGTTA
CSF1R	GCGGGACTATACCAATCTGC	AGCAGGTCAGGTGCTCACTA
CTSG for ChIP	AGACCGTGTAATCCAAGCCA	TCTCGGCACTGACTTAGCAG
CTSG for DNase I	GGTTTCATCACCCAAGGCTG	TGGCTTGGATTACACGGTCT
CTSG for mRNA	TCCTGGTGCGAGAAGACTTTG	GGTGTTTTCCCGTCTCTGGA
GAPDH	CCTGGCCAAGGTCATCCAT	AGGGGCCATCCACAGTCTT
GATA2	CAGACGAAGGCAACCATTTT	GCTCAGACCACCAAGTCTCC
HOXA9	GTGATGCCATTTGGGCTTAT	GGGGTGAGAGAAGGGAGAAG
IVL	GCCGTGCTTTGGAGTTCTTA	CCTCTGCTGCTGCCACTT
MEIS1	CAGAAAAAGCAGTTGGCACA	GGTCTATCATGGGCTGCACT
MPO for mRNA	CCAACAACATCGACATCTGG	GCTGAACACACCCTCGTTCT
MPO for ChIP	CAACACACTCACACCCCACT	TGGGAACTCTAAGTGGGCAG
PU.1 3H Enh	AACAGGAAGCGCCCAGTCA	TGTGCGGTGCCTGTGGTAAT
RUNX1	CCCTCAGCCTCAGAGTCAGAT	AGGCAATGGATCCCAGGTAT
RUNX1-EVI1	CCACAGAGCCATCAAAATCA	TCTGGCATTTCTTCCAAAGG
SIGLEC1	GTATCAGGGGCTGCTTCCTC	CTGGGTTGGACAGTAGAGCT
TBP promoter (long)	CTGGCGGAAGTGACATTATCAA	GCCAGCGGAAGCGAAGTTA
TBP promoter (short)	CTGGCGGAAGTGACATTATCAA	CCCGACCTCACTGAACCC
TREM1	ACAAGGCACCACAATGACCT	GGCCTCATATCCTGTTGTGC
**siRNA sequences**		
RUNX1-EVI1 (Axolabs, Germany)	GAACCUCGAAAUAAUGAGUGU	ACUCAUUAUUUCGAGGUUCUC
RUNX1 (Dharmacon, ON-TARGETplus)	not available	not available

GATA2 (Origene, SR301743)	not available	not available

Antibodies

Antibodies for probing Western blots		
**Antibody target**	**Company**	**Serial number**
EVI1	Cell Signalling	2593
RUNX1 (C-terminal epitope)	Abcam	23980
RUNX1 (N-terminal epitope)	Cell Signalling	4334
C/EBPα	Abcam	40761
FLAG epitope	Sigma	F3165
GAPDH	Abcam	8245
Anti-Rabbit HRP	Cell signalling	7074
Anti-Mouse HRP	Jackson	115 035 062
**Antibodies for ChIP**		
**Antibody target**	**Company**	**Serial number**
EVI1	Cell Signalling	2593
RUNX1 (C-terminal epitope)	Abcam	23980
C/EBPα	Santa Cruz	A2814
GATA2	R+D	AF2046
**Antibodies for flow cytometry**		
**Antibody target-flurochrome**	**Company**	**Serial number**
CD34-APC	Miltenyl-biotech	10098139
CD34-PE	Mitlenyl-biotech	130081002
CD117-APC (Clone A3C6E21)	Mitlenyl-biotech	130091733
CD11b-APC	Miltenyl-biotech	130091241
CD11b PE (Clone M1/70)	Ebiosciences	120011281
CD14-FITC	Miltenyl-biotech	130080701
CD14-PE (Clone MΦP9)	BD Biosciences	562691
Annexin V-FITC/PI kit	BD Pharmingen	556547
Annexin V-APC/PI kit	Ebiosciences	88-8005-74
IgG FITC	Miltenyl-biotech	130093192
IgG PE	Miltenyl-biotech	130093193
IgG APC	Miltenyl-biotech	130093194

DATA ANALYSIS

ChIP and DNase I sequencing data Analysis

Alignment

Sequences from all ChIP and DNase I sequencing experiments in fastq format were mapped onto the reference human genome version hg38, Genome Reference Consortium GRCh38. The quality control statistics for the samples were obtained using FastQC software (http://www.bioinformatics.babraham.ac.uk/projects/fastqc/). The raw reads were aligned to the reference genome using Bowtie2 (Langmead and Salzberg, 2012). Reads from ChIP-seq data that were uniquely aligned to chromosomal positions were retained and duplicate reads were removed from the aligned data using Picard tools (http://broadinstitute.github.io/picard/). The aligned reads were used to generate density profiles using "genomeCoverageBed" function from bedtools (http://bedtools.readthedocs.org/en/latest/). These tag densities were displayed using the UCSC Genome Browser (Kent et al., 2002). The numbers of aligned reads are listed in Table S5. RUNX/ETO ChIP is the combined of a publically available data downloaded from GEO with accession numbers GSM1113429**,** GSM1113430 (Ben-Ami, et. al, 2013) and GSM1082306 (Wang et. al, 2013).

Peak calling

Regions of enrichment (peaks) of ChIP and DNAse1 sequencing data were identified using DFilter software (Kumar et al., 2013) with recommended parameters (-bs=100 -ks=50 –refine). Peak overlaps, gene annotations were performed using in-house scripts. High confidence ChIP-Seq peaks were defined as those overlapping peaks in the DNase-Seq data. Overlaps between ChIP and DNase I sequencing were defined by requiring the summit of a peak in the ChIP dataset to lie between start and end coordinates of a peak in the DNase I data. Peaks were allocated to genes if located in either their promoters or within the region of 2000 bp downstream and 2000 bp upstream of the transcription start sites (TSS), as intragenic if not in the promoter but within the gene body region, or if intergenic, to the nearest gene located within 100 kb. Overlaps between ChIP-seq peaks were defined by requiring the summits of two peaks to lie within +/-200 bp.

Clustering of ChIP and DNAseI sequencing data

Hierarchical clustering with Euclidean distance and complete linkage clustering was used for clustering of transcription factors based on similar binding patterns of different ChIP-seq data, in SKH-1 cells. The high confidence peaks for all transcriptional factors were intersected and merged when overlapping. The read counts for all union peaks were normalized with regards to total reads depth counts and then Pearson's correlation coefficients were calculated between samples using log2 of the normalized read counts. A correlation matrix was generated and Pearson correlation coefficients are displayed after hierarchical clustering as a heatmap. Colors in the heatmap indicate the strength of association between each pair of transcription factors. Heat maps were generated using Mev

from TM4 microarray software suite (Saeed et al., 2006). Same way was used for DNase-Seq data clustering.

Average tag density profile and heatmap

The tag density and average profiles for Figure 1E were generated by calculating the tag density normalized as coverage per million within 4 kb of the DNAse1 peak summit. The read counts for all union peaks were computed. Coverages were calculated for all union peaks and ranked by log2 fold change. Heatmap images were generated via Java TreeView (http://jtreeview.sourceforge.net/) and average profiles were plotted using R (https://www.r-project.org/).

Motif identification and clustering

De novo motif analysis was performed on peaks using HOMER (Benner et.al 2010). Motif lengths of 6, 8, 10, and 12 bp were identified in within ± 200 bp from the peak summit. The top enriched motifs with a significant p value score were recorded. The annotatePeaks function in HOMER was used to find occurrences of motifs in peaks. In this case we used known motif position weight matrices (PWM) from HOMER database.

Motif clustering

Digital footprinting of t(3;21) AML patients 1 and 2 and t(8;21) AML patients 1 and 2 from DNase I high-depth sequencing data was performed using the Wellington algorithm (Piper et al., 2013) with FDR=0.01. For the heatmap that shows hierarchical clustering of motif occurrences within RUNX1/EVI1 footprints (Figure 3E), a motif positions search was done within peaks that are only footprinted in t(3;21) patients. The distance between the centers of each motif pairs was calculated and the motif frequency was counted if the first motif was within 50bps distance from the second motif. Z-scores were calculated from the mean and standard deviation of motif frequencies observed in random sets using bootstrap analysis. For bootstrapping, peak sets with a population equal to that of the footprinted peaks were randomly obtained from the union of t(3;21), t(8;21) and CD34+ DNase-Seq footprints. Motif search was repeated for each random set and then the mean and the standard deviation for the total motif frequencies of the random peak sets were calculated and compared with the actual motif frequencies to obtain the Z- scores. A matrix was generated and Z scores were displayed after hierarchical clustering as a heat map. Red color means that motifs are overrepresented and grey color indicates that motif is underrepresented. The same procedure was repeated with RUNX1/ETO and RUNX1 peaks (Figure S3 and 3F) that are only footprinted in t(8;21) patients and where motif search was done exactly within the footprint coordinates and the random sets were generated from the total patient’s footprints.

Motif enrichment

To identify motifs (identified by HOMER) that are relatively enriched in the distal transcription factors (TFs) sites of one cell type compared to another or one TF compared to another from same cell type we considered all possible comparisons (Figure 3F), these being TF sites in

(A) which are not shared with each of the other TFs (B). For a given set *j* of TFs, we defined a motif enrichment score (S_{ij}) for motif *i* in peak set *j* as

$$S_{ij}\frac{n_{ij}/M_j}{\sum_j n_{ij}/\sum_j M_j}$$

where n_{ij} is the number of peaks in each subset *j (j=1,2,…,12)* containing motif *i (i=1, 2,….,I), I* is the total number of motifs used in the test, and M_j the total number of peaks in each subset *j (j=1,2,…,30).* A matrix was generated and the motif enrichment scores were displayed as a heatmap after hierarchical clustering with Euclidean distance and complete linkage. The heatmap was generated using Mev from TM4 microarray software suite (Saeed et al., 2006).

RNA-Seq data Analysis

RNA-Seq reads were aligned to the hg38 human genome build using STAR. Separate density profiles for the positive and negative strand were generated for RNA-Seq data. Fragments per Kilobase of transcript per Million mapped reads (FPKM) values for each gene were extracted using Cufflinks and differentially expressed genes were extracted using the limma R package (Ritchie et al., 2015). All genes with p-value ≤ 0.01 were considered and at least 1.5-fold changes between before and after RUNX1/EVI1 knock down. The differentially expressed genes for the AML patients were considered with at least 2-fold changes using the CD34+ PBSC as a control. The numbers of aligned reads are listed in Table S5.

The correlation between any two samples was obtained as the Pearson correlation coefficient of expression values over all genes. A correlation matrix was thus generated for all the samples and hierarchically clustered.

Clustering of gene expression was carried out on signal intensity for all expressed genes and on fold-changes for genes associated with at least a 1.5-fold change. Hierarchical clustering was used with Euclidean distance and average linkage clustering. Heatmaps were generated using Mev (Saeed et al., 2006).

The GSEA software (Subramanian et al., 2005) was used to perform gene set enrichment analysis on group of genes. The normalized enrichment score (NES), the p-value and the FDR q-value are displayed on the enrichment plot.

Gene ontology (GO) analysis was performed using Bingo (Maere et al., 2005) and David online tool at david.abcc.ncifcrf.gov (Huang da et al., 2009) using Hypergeometric for overrepresentation and Benjamini and Hochberg (FDR) correction for multiple testing corrections. KEGG Pathway network analysis was performed using clueGO tools (Bindea et al., 2009) with kappa score = 0.3. The right-sided enrichment (depletion) test based on the hypergeometric distribution was used for terms and groups. Groups were created by iterative merging of initially defined groups based on the kappa score threshold. The relationship between the selected terms is defined based on their shared genes and the final groups are randomly colored where one, two colors or more represents that a gene/term is a member of

one, two or more groups respectively. The size of the nodes reflects the enrichment significance of the terms. The network is laid out using the layout algorithm supported by Cytoscape.

Supplemental References

1. Bindea, G., Mlecnik, B., Hackl, H., Charoentong, P., Tosolini, M., Kirilovsky, A., Fridman, W.H., Pages, F., Trajanoski, Z., and Galon, J. (2009). ClueGO: a Cytoscape plug-in to decipher functionally grouped gene ontology and pathway annotation networks. Bioinformatics *25*, 1091-1093.
2. Ben-Ami O, Friedman D, Leshkowitz D, Goldenberg D et al. Addiction of t(8;21) and inv(16) acute myeloid leukemia to native RUNX1. Cell Rep 2013 Sep 26;4(6):1131-43.
3. Bomken S, Buechler L, Rehe K, Ponthan F, Elder A, Blair H, Bacon CM, Vormoor J, Heidenreich O. Lentiviral marking of patient-derived acute lymphoblastic leukaemic cells allows in vivo tracking of disease progression. Leukemia. 2013 Mar;27(3):718-21.
4. Heinz S, Benner C, Spann N, Bertolino E et al. Simple Combinations of Lineage-Determining Transcription Factors Prime cis-Regulatory Elements Required for Macrophage and B Cell Identities. Mol Cell 2010 May 28;38(4):576-589.
5. Kent WJ, Sugnet CW, Furey TS, Roskin KM, Pringle TH, Zahler AM, Haussler D., The human genome browser at UCSC. Genome Res. 2002 Jun;12(6):996-1006.
6. Kumar V, Muratani M, Rayan NR, Kraus P, Lufkin T, Ng HH and Prabhakar S, Uniform, optimal signal processing of mapped deep-sequencing data, Nature biotechnology, 31, 615-622 (2013)
7. Langmead B, Salzberg S. Fast gapped-read alignment with Bowtie 2. Nature Methods.2012, 9:357-359.
8. Maere, S., Heymans, K., and Kuiper, M. (2005). BiNGO: a Cytoscape plugin to assess overrepresentation of gene ontology categories in biological networks. Bioinformatics *21*, 3448-3449.
9. Piper, J., Elze, M.C., Cauchy, P., Cockerill, P.N., Bonifer, C., and Ott, S. (2013). Wellington: a novel method for the accurate identification of digital genomic footprints from DNase-seq data. Nucleic Acids Res *41*, e201.
10. Ritchie et., al. *limma* powers differential expression analyses for RNA-sequencing and microarray studies. Nucl. Acids Res. *(2015)*
11. Saeed, A.I., Bhagabati, N.K., Braisted, J.C., Liang, W., Sharov, V., Howe, E.A., Li, J., Thiagarajan, M., White, J.A., and Quackenbush, J. (2006). TM4 microarray software suite. Methods Enzymol *411*, 134-193.
12. Subramanian, A., Tamayo, P., Mootha, V.K., Mukherjee, S., Ebert, B.L., Gillette, M.A., Paulovich, A., Pomeroy, S.L., Golub, T.R., Lander, E.S*., et al.* (2005). Gene set enrichment analysis: a knowledge-based approach for interpreting genome-wide expression profiles. Proc Natl Acad Sci U S A *102*, 15545-15550.
13. Sun XJ, Wang Z, Wang L, Jiang Y et al. A stable transcription factor complex nucleated by oligomeric AML1-ETO controls leukaemogenesis. Nature 2013 Aug 1;500(7460):93-7.

14. X Jiao, BT Sherman, R Stephens, MW Baseler, HC Lane, RA Lempicki. DAVID-WS: a stateful web service to facilitate gene/protein list analysis. Bioinformatics (2012) 28 (13): 1805-1806.

Supplemental tables

Table S1 Table S1 is an attached Excel file available via the website.

Title: Gene expression levels in patient cells and cell lines (RNA-Seq). Related to Figures 1 and 4

Sheet 1) RNA-Seq data from CD34+ cells purified from patients with either t(3;21) or t(8;21) AML, and from normal PBSCs. Related to Figure 1.
RNA-Seq values are expressed as FPKM.

Sheet 2) RNA-Seq data from SKH-1 cells following RUNX1-EVI1 siRNA transfection as compared to control siRNA transfection. Related to Figure 4
RNA was extracted after 2, 4 and 10 days of treatment. Values are expressed as FPKM values from RNA-Seq in control siRNA (MM) and RUNX1-EVI1 siRNA (KD) transfected cells. Gene expression fold change (FC) is given following either 2, 4 or 10 days of siRNA transfection.

Table S2 Patient details

Details of patient samples included in this study. t(8;21) patient 1 and 2 were processed before the commencement of this study.

ID	White cell count X 10^9/L	Age	Sex	Stage of treatment	Cytogenetics	Clinical notes	Mutations
t(3;21) # 1	23	44	F	Presentation	46,XX,t(3;21)(q26;q22), der(5)t(5;13)(q2;q3),der(7)t(1;7)(q3;q3[5]/47, idem,+12,+der(21), t(3;21)[4]	Therapy related AML (previous Myelofibrosis and T cell lymphoma)	KRAS
t(3;21) # 2	54	72	M	Presentation	46,XY,t(3;21)(q26;q22), del(12)(p12p13)[20]	RAEB-t	DNMT3, SRSF2
t(8;21) # 1	2.12	45	M	Presentation	46,XY,t(8;21)(q22;q22)	AML	None found
t(8;21) # 2	53	53	M	Presentation	46,XY,t(8;21)(q22;q22)	AML	CBL, FLT3-TKD
PBSC # 1	N/A	51	M	N/A	N/A	Mobilized PBSCs from sibling donor for allogeneic stem cell transplant	N/A
PBSC # 2	N/A	47	F	N/A	N/A	Mobilized PBSCs from autologous donor for stem cell transplant (CNS lymphoma)	N/A

Table S3 DNA Sequencing data and peak numbers of the experiments indicated in the table below (related to Figures 1,2,3, 6)

**DNase-Seq reads alignment and peaks detected in t(3;21) SKH-1 cell line, primary CD34+ AML blasts and normal CD34+ PBSC.**			
**DNase-Seq Dataset**	**Aligned reads**	**Total Peaks**	
CD34 J209 (PBSC 1)	173,261,382	36,041	
CD34 R299 (PBSC 2)	205,284,104	36,088	
t(3;21) SKH1 cell line	348,530,966	28,821	
GT027 (t(3;21) patient 1	407,335,267	31,532	
AML5354 (t(3;21) patient 2	237,196,453	35,666	
H12812 (t(8;21) patient 1	405,680,774	32,262	
H18901 (t(8;21) patient 2	387,658,545	35,052	
**ChIP-seq in untreated t(3;21) SKH-1, and normal CD34+ PBSC** (High confidence peaks are ChIP-seq peaks that overlap with a DHS.)			
**ChIP Dataset**		**Total peaks**	**High Confidence Peaks**
Anti-RUNX1 SKH1		15,609	10,922
Anti-EVI1 SKH1		14.992	8,947
Anti-RUNX1 CD34+PBSC		13,951	11,226
**DNase-Seq alignments from SKH-1 after 10 days treatment of control or RUNX1-EVI1 siRNA**			
**DNase-Seq Dataset**	**Aligned reads**	**Total peaks**	
Control siRNA Day 10	8,531,753	16,456	
RUNX1-EVI1 siRNA Day 10	19,976,087	18,672	
**ChIP-Seq read alignments from SKH-1 after 10 days (D10) treatment of control (MM) or RUNX1-EVI1 siRNA (KD)** (High confidence peaks are ChIP-seq peaks that also coincide with DHSs.)			
**ChIP-seq Dataset**	**Aligned reads**	**Peaks**	**High Confidence Peaks**
Anti-EVI1 D10 MM	20,350,942	12,842	7,252
Anti-EVI1 D10 KD	29,752,295	2,014	1,208
Anti-RUNX1 D10 MM	19,147,420	15,185	10,346
Anti-RUNX1 D10 KD	20,959,942	15,592	9,944
Anti- C/EBPα D10 MM	23,509,134	9,346	7,016
Anti- C/EBPα D10 KD	29,564,903	11,808	8,639
Anti- GATA2 MM	44,761,095	14,640	8,100
Anti- GATA2 KD	22,408,237	13,628	9,791

Table S4 RNA-Seq read alignments of the experiments indicated in the table below (related to Figures 1 and 4)

**RNA-Seq data in t(3;21) SKH-1 cell line, primary CD34+ AML blasts and normal CD34+ PBSC.**	
**RNA-Seq Dataset**	**Aligned reads**
t(3;21) SKH-1 cell line replicate 1	30,334,985
t(3;21) SKH-1 cell line replicate 2	11,898,204
CD34 R454 (PBSC # 1)	16,538,775
CD34 R423 (PBSC # 2)	12,402,996
H12812 (t(8;21) patient 1)	12,347,883
H18901 (t(8;21) patient 2)	11,331,201
GT027 (t(3;21) patient 1)	48,166,460
AML5354 (t(3;21) patient 2)	11,969,780
**RUNX1-EVI1 or control siRNA transfected SKH-1 RNA-Seq data**	
Control siRNA (siMM) or RUNX1-EVI1 siRNA (siREVI1) transfected cells. RNA -Seq in SKH-1 cells after either 2 (D2), 4 (D4) or 10 (D10) days of siRNA treatment. Two biological replicates performed (Rep1 and Rep2).	
**RNA-Seq Dataset**	**Aligned reads**
siMM_D2_Rep1	43,584,690
siMM_D2_Rep2	41,344,409
siMM_D4_Rep1	33,410,586
siMM_D4_Rep2	44,832,782
siMM_D10_Rep1	22,705,489
siMM_D10_Rep2	22,419,529
siREVI1_D2_Rep1	43,782,238
siREVI1_D2_Rep2	23,157,018
siREVI1_D4_Rep1	37,541,312
siREVI1_D4_Rep2	53,187,360
siREVI1_D10_Rep1	17,656,510
siREVI1_D10_Rep2	30,380,957
